# Responses to auxin signals: an operating principle for dynamical sensitivity yet high resilience

**DOI:** 10.1098/rsos.172098

**Published:** 2018-01-24

**Authors:** S. Grigolon, B. Bravi, O. C. Martin

**Affiliations:** 1 LPTMS, Université Paris-Sud XI-Université Paris-Saclay, 15, Rue Georges Clémenceau, 91405 Orsay, France; 2 Department of Mathematics, King’s College London, The Strand, London WC2R 2LS, UK; 3 GQE-Le Moulon, INRA, Université Paris-Sud, CNRS, AgroParisTech, Université Paris-Saclay, 91190 Gif-sur-Yvette, France

**Keywords:** signalling networks, operating principles, linear response function

## Abstract

Plants depend on the signalling of the phytohormone auxin for their development and for responding to environmental perturbations. The associated biomolecular signalling network involves a negative feedback on Aux/IAA proteins which mediate the influence of auxin (the *signal*) on the auxin response factor (ARF) transcription factors (the *drivers* of the response). To probe the role of this feedback, we consider alternative *in silico* signalling networks implementing different operating principles. By a comparative analysis, we find that the presence of a negative feedback allows the system to have a far larger sensitivity in its dynamical response to auxin and that this sensitivity does *not* prevent the system from being highly resilient. Given this insight, we build a new biomolecular signalling model for quantitatively describing such Aux/IAA and ARF responses.

## 1. Introduction

Plants are sessile, so they are subject to stronger environmental constraints than animals. Unable to change their location, they respond to changing conditions by modifying their physiology, leading to protection against abiotic stress (cold, drought, wind, etc.) as well as biotic stress (fungi, insects, etc.). The underlying molecular mechanisms of signalling, that is for going from sensing environmental conditions to changing physiology, largely rely on metabolites that act as hormones: auxin, gibberellins, cytokinins, jasmonates, etc. Auxin is perhaps the most ubiquitous of plant hormones, playing its role of signalling in such diverse physiological phenomena as phototropism, gravitropism, wound response and leaf abscission [1]. Its scientific name is indole-3-acetic acid, and in fact the term ‘auxin’ covers a *family* of related metabolites containing an aromatic ring and a carboxylic acid group.

Our focus here is on the molecular workings within cells which take the auxin signal and exploit it. A central actor in initiating downstream changes is a family of transcription factors, the *auxin response factors* (hereafter denoted as ARFs) [2–4]. Their responses to auxin are to transcriptionally activate downstream genes which either drive developmental processes (such as cell elongation or differentiation) or change physiology (e.g. opening of stomata). Depending on the cellular context, different ARFs will respond to the auxin signal and different downstream target genes will be affected. The detailed function of each ARF is still to be understood, but qualitatively the mechanism at work is shared across all ARFs as follows.

First, in the absence of an auxin signal, ARF proteins are present but they are sequestered by heterodimerization with another family of proteins, the Aux/IAAs [2,5]. The different Aux/IAAs bind with various specificities to the different ARFs, a feature which is probably key to the many possible cellular responses generated upon auxin signalling. Hereafter, we shall refer to Aux/IAA as IAA protein or simply ‘IAA’ to lighten the notation, in particular in our equations. Second, when auxin enters the cell, it binds to IAA protein; the formation of this complex allows the rapid ubiquitination of IAA which leads to its degradation by the proteosome [6,7]. The concomitant decrease in the amount of IAA protein leads to the unsequestering of ARF which thus becomes available for transcriptional activation of its target genes. As auxin leads to the degradation of IAA protein, in effect it acts negatively on IAA. Similarly, because IAA sequesters ARF, IAA in effect acts negatively on ARF. An interesting characteristic of this system is that ARF is only sequestered, it is not degraded. As a result, the driver of the downstream responses (ARF) can trigger its targets very quickly upon arrival of the signal; there are no delays coming from having to transcribe and translate the driver. Such fast responses contribute to the survival of the plant. But there is another characteristic shared across these auxin-signalling systems: IAA effectively acts negatively on its own transcription. At the molecular level, this occurs via inhibition of IAA transcription, for instance by ARF–IAA heterodimers [8,9]. What is the ‘logic’ of such a negative feedback? Feedbacks are found in many systems, be they natural or man-made. Negative feedbacks in genetic networks have been shown to be important both for steady-state and for dynamical behaviours [10]. For instance, a system having an unstable steady state might run away (diverge) unless there is a (nonlinear) negative feedback to prevent that. Although negative feedbacks may maintain homeostasis and reduce the noise of a network’s output, they can also generate oscillatory behaviour. It is thus no surprise that negative feedbacks are found in the genetic networks driving circadian rhythms [11] or segmentation clocks during development [12]. In our particular case, might the negative feedback allow for a fast response to auxin signalling [13], or could it increase the robustness of the system [14]? In the different models that have been proposed for auxin signalling [15–17], this feedback is included but the question of its role has not been considered. Our goal here is to determine whether such a negative feedback can be justified through its consequences on the *dynamical* properties of the signalling, thereby revealing an operating principle of these systems.

To investigate the role of the IAA negative feedback, we first consider a particularly minimal model in §1, allowing us to demonstrate the generic advantage for dynamical responses of having such feedback. This example is remarkable because the steady states can be imposed to be exactly the same whether there is feedback or not, while the dynamical behaviour is very sensitive to the feedback as it allows for much greater sensitivity and resilience. Then in §§2 and 3, we reconsider previously published models of auxin signalling [15–17]. As in our minimal model, these models involve a single generic ARF and a single generic IAA, and thus they also do not address the subtle points associated with having multiple members of those protein families [18]. Nevertheless, they include key actors and regulatory processes. The model in [16] has in fact been calibrated, so we focus on it to perform our in-depth analyses (§2). The complexity of that model pushes us towards both mathematical and computational tools, from which we determine numerically the steady states and different dynamical responses. Our results confirm the operating principle behind the negative feedback: it allows great dynamical sensitivity over a broad range of signal intensities while ensuring high resilience. Lastly, given the conclusions obtained by these analyses, we propose in §3 a new quantitative model to describe the dynamics of auxin signalling. After calibrating this model, we illustrate its properties and consider in §4 consequences at the level of downstream genes that are targets of ARFs.

## 2. Results from a minimal model

**Figure 1.**
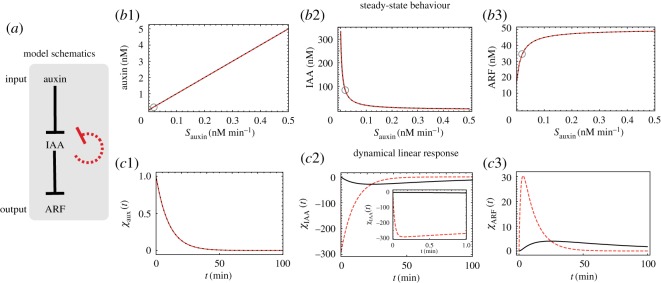
Properties of the minimal model. (*a*) The molecular actors in our minimal model are auxin (the signal), IAA protein (the mediator) and ARF (the driver of the downstream effect). Also shown are the (oriented) interactions via a blunt arrow to indicate feed-forward inhibition (auxin on IAA on the one hand and IAA on ARF on the other) as well as the negative feedback (in dashed red) whereby an increase in concentration of IAA inhibits further production of IAA. (*b*) The steady-state concentrations as a function of *S*_*auxin*_ (the incoming flux of auxin); *b*1: case of the signalling molecule auxin; *b*2: case of the protein IAA; *b*3: case of the transcription factor ARF. By construction, the regulated (dashed red lines) and unregulated (solid black lines) cases have identical input–output relation in the steady state (see text). Parameter values: $\tau_{\mathrm{auxin}}=10\;\min$, $\tau_{\mathrm{IAA}}=333\;\min$, $\tau_{\mathrm{ARF}}=2\;\min$, $\alpha^{(\mathrm{no}\text{-}reg)}=0.05\;{\left(\mathrm{nM}\;\min\right)}^{-1}$, *α*^(*reg*)^=150 (*nM* *min*)^−1^, $S_{\mathrm{IAA}}=1\;\mathrm{nM}\;\min^{-1}$, $S_{\mathrm{ARF}}=25\;\mathrm{nM}\;\min^{-1}$ and $\beta =0.003\;{\left(\mathrm{nM}\;\min\right)}^{-1}$. (*c*) The dynamical linear response functions for auxin (*c*1), IAA (*c*2) and ARF (*c*3) in the unregulated and regulated minimal models. The inset of *c*2 is to show that the response function, though very steep, is continuous. Parameter values and line type/colour are as in *b*. $S_{\mathrm{auxin}}=0.02\;\mathrm{nM}\;\min^{-1}$ and the corresponding steady-state concentrations of the three species are marked with a circle in *b*.

We first consider a minimal model in which the lower number of molecular actors and processes reduces the system’s complexity. Its mathematical analysis will provide the hoped-for insights quite directly. The molecular species of our minimal model are auxin, IAA (no distinction between messenger RNA and protein) and ARF (see also electronic supplementary material, §SI). Given the characteristics of the processes discussed in the introduction, we take (i) auxin to act negatively on IAA, a proxy for the degradation of IAA induced by auxin; (ii) IAA to act negatively on ARF, a proxy for the sequestering of ARF by IAA; and (iii) IAA to inhibit its own expression, a proxy for the negative feedback of interest. This system is a two-step feed-forward cascade [10]. Note that each feed-forward interaction is negative and as a result, auxin acts positively on ARF. This very simple network is represented in figure 1*a*. We shall be interested both in the model with feedback and in the model obtained by omitting such feedback, hereafter referred to as the ‘unregulated’ model. We denote by [auxin], [IAA] and [ARF] the concentrations of the three corresponding molecular species. The ‘action’ of one molecular species on another is modelled by simple kinetics with rates being linear in the respective concentrations. The minimal model is then defined by three ordinary differential equations, one for each of the three species:

2.1 $$\begin{array}{rl} \frac{d[\mathrm{auxin}]}{dt} & =S_{\mathrm{auxin}}-\frac{\left[\mathrm{auxin}\right]}{\tau_{\mathrm{auxin}}}, \end{array}$$

2.2 $$\begin{array}{rl} \frac{d[\mathrm{IAA}]}{dt} & =S_{\mathrm{IAA}}-\frac{\left[\mathrm{IAA}\right]}{\tau_{\mathrm{IAA}}}-\alpha [\mathrm{auxin}][\mathrm{IAA}] \end{array}$$

2.3 $$\begin{array}{rl} \;\text{and}\;\frac{d[\mathrm{ARF}]}{dt} & =S_{\mathrm{ARF}}-\frac{\left[\mathrm{ARF}\right]}{\tau_{\mathrm{ARF}}}-\beta [\mathrm{IAA}][\mathrm{ARF}]. \end{array}$$

In these equations, *τ*_*auxin*_, *τ*_*IAA*_ and *τ*_*ARF*_ are the lifetimes of, respectively, auxin, IAA and ARF due to spontaneous decay of those molecular species. To compensate these finite lifetimes, each molecular species must be either produced or imported. For instance, auxin is imported into the cell, thus the presence of the source term *S*_*auxin*_, whereas IAA and ARF are synthesized within the cell. Note that although we take *S*_*ARF*_ to be fixed, *S*_*IAA*_ will depend on the concentration of IAA when allowing IAA to be self-inhibitory. Specifically, in our ‘regulated’ model, *S*_*IAA*_ is taken to be a decreasing function of [IAA] to implement the self-inhibition (negative feedback loop). Is it possible that the negative regulation, as found across plants today, is present because it leads to ‘better’ signalling than if there is no regulation? Of course, it is difficult to be sure what ‘better’ corresponds to. One guess is that the regulation allows one to postpone the saturation of the output, pushing that regime to larger values of the input flux *S*_*auxin*_. Interestingly, this guess is incorrect because the system without regulation can do that also. By decreasing *α*, the range in *S*_*auxin*_ over which the steady-state values of [IAA] and [ARF] avoid saturation can be increased at will, no regulatory feedback is necessary. Since a small value of *α* corresponds to a weak interaction strength between auxin and IAA, it is always possible to have *α* as small as desired. Nevertheless, there is a real drawback if *α* is small: the stability of the steady state will be low, and thus the return to the steady state after a perturbation will take a long time.

The minimal model not only shows how the negative feedback on IAA makes such contradictory features coexist, but it also lets one focus on the dynamical properties of this system. Indeed, the simplicity of the equations allows one to impose that the regulated and non-regulated cases have *exactly* the same steady-state curves. As explained in the electronic supplementary material (cf. electronic supplementary material, eq. S5), the use of a specific functional form for *S*^(*reg*)^_*IAA*_ ensures the independence of the steady states on the presence/absence of the transcriptional regulation and thereby provides a particularly elegant framework for probing the role of the feedback. This situation is illustrated in figure 1*b*1–*b*3.

In the mathematical study of this minimal model, it is straightforward to determine the steady-state concentrations as a function of *S*_*auxin*_. Then, given this steady state, we proceed to analyse the linearized dynamics to understand the behaviour of the system when it is slightly perturbed about its steady state. It is easy to see that the steady state is stable for all values of auxin incoming flux. In addition, by studying the associated eigenvalues of the stability matrix as we have done using Mathematica (cf. Mathematica notebook provided in the electronic supplementary material), we find that regulation leads to *enhanced* stability. From this result, one may say that the regulated system is more *resilient* than the unregulated one. The term resilience refers to the ability of a system to come back to its natural state after having been deformed or perturbed. Elastic systems are resilient, while plastic ones are not. A system can be robust (resist change) without being resilient (it will break under too much stress), while a resilient system will possibly deform significantly but once no longer subject to the stress, it will quickly come back to its initial state. A quantitative measure of resilience of use here is the time for a perturbation to relax away. The shorter this time, the more resilient the system is. Based on the values of the eigenvalues found in the presence and absence of regulation, we see that the negative feedback enhances resilience in our minimal model.

Such notions of sensitivity and resilience can be further investigated via the time-dependent response of the system to a perturbation. For our purposes here, we take the perturbation to be an instantaneous incoming pulse of auxin. When the pulse intensity is small, the linearized dynamics determines the system’s corresponding *linear dynamical response function* for each of the molecular species. These responses are displayed for the models with and without regulation in figure 1*c*2,*c*3 and are hereafter denoted as *χ*_*aux*_(*t*), *χ*_*IAA*_(*t*) and *χ*_*ARF*_(*t*). We see that the effects of regulation are to allow for an enhancement of the *amplitude* of IAA and ARF responses by a factor of about *α*^(*reg*)^/*α*^(*no*-*reg*)^ (depending on the details of the relative relaxation rates) while at the same time preserving their total (integrated over time) response. The qualitative insight obtained by considering this minimal model is thus that regulation ‘compresses’ the total response (a conserved quantity in the linearized framework; cf. mathematical proof in electronic supplementary material, §SI) into a shorter period, and by doing so it necessarily increases the amplitude of the dynamical response. These insights will now be transferred to the more realistic models.

## 3. Results for statics versus dynamics in the model of Vernoux *et al.*

In 2011, Vernoux *et al.* [16] proposed a quantitative and fully calibrated model of auxin signalling. Their model is available in the repository BioModels [19] along with all the values of the associated parameters. To our knowledge, this is the only completely parametrized and publicly available model of auxin signalling. The aim of this section is to assess the impact of transcriptional regulation in that model, building on the insights coming from our minimal model.

### 3.1. Model components, processes and equations

In the Vernoux model, the molecular species are auxin, IAA messenger RNA denoted by IAA_*m*_, IAA protein, ARF, the IAA homodimer denoted by IAA_2_, the ARF homodimer denoted by ARF_2_ and finally, the ARF–IAA heterodimer. The concentration of auxin is taken as a control parameter to be set externally rather than as a dynamical variable. The other species follow dynamics given by ordinary differential equations motivated by the different processes involved. Except for the transcription of IAA mRNA, all rates are given by mass-action-type ordinary differential equations as follows:

3.1 $$\begin{array}{rl} \frac{d[\mathrm{IAA}]}{dt} & =\pi_{I}[{\mathrm{IAA}}_{m}]+2k_{\mathrm{II}}^{\prime }[{\mathrm{IAA}}_{2}]-2k_{\mathrm{II}}{\left[\mathrm{IAA}\right]}^{2}+k_{\mathrm{IA}}^{\prime }[\mathrm{ARF}–\mathrm{IAA}]-k_{\mathrm{IA}}[\mathrm{IAA}][\mathrm{ARF}]\\ & \;+\delta_{\mathrm{II}}(x)[{\mathrm{IAA}}_{2}]-\delta_{I}(x)[\mathrm{IAA}], \end{array}$$

3.2 $$\begin{array}{r} \frac{d[\mathrm{ARF}]}{dt}=\pi_{A}+k_{\mathrm{IA}}^{\prime }[\mathrm{ARF}–\mathrm{IAA}]-k_{\mathrm{IA}}[\mathrm{IAA}][\mathrm{ARF}]+\delta_{\mathrm{IA}}(x)[\mathrm{ARF}–\mathrm{IAA}]-\delta_{A}[\mathrm{ARF}], \end{array}$$

3.3 $$\begin{array}{r} \frac{d[{\mathrm{IAA}}_{2}]}{dt}=k_{\mathrm{II}}{\left[\mathrm{IAA}\right]}^{2}-(k_{\mathrm{II}}^{\prime }+\delta_{\mathrm{II}}^{*}+\delta_{\mathrm{II}}(x))[{\mathrm{IAA}}_{2}], \end{array}$$

3.4 $$\begin{array}{r} \frac{d[\mathrm{ARF}-\mathrm{IAA}]}{dt}=k_{\mathrm{IA}}[\mathrm{IAA}][\mathrm{ARF}]-(k_{\mathrm{IA}}^{\prime }+\delta_{\mathrm{IA}}^{*}+\delta_{\mathrm{IA}}(x))[\mathrm{ARF}–\mathrm{IAA}] \end{array}$$

3.5 $$\begin{array}{r} \;\text{and}\;\frac{d[{\mathrm{IAA}}_{m}]}{dt}=h([\mathrm{IAA}],[\mathrm{ARF}],[\mathrm{ARF}–\mathrm{IAA}])-\delta_{R}[{\mathrm{IAA}}_{m}]. \end{array}$$

In these equations, we have used the same symbols for the parameters as in [16], but use our notation for the different molecular species. Furthermore, *π*_*I*_ is the (constant) rate of translation of IAA messenger RNAs, *π*_*A*_ is the production rate of ARF (directly for the protein, englobing both transcription and translation), and *k*_*IX*_ and *k*′_*IX*_ (with *X*=(*I*,*A*)) are the association and dissociation rates of the dimerizations, respectively.

Note that transcription of IAA is explicitly included via the function *h*, thereby allowing for modelling transcriptional regulation. Indeed, Vernoux *et al.* [16] take the rate of production of IAA mRNA to depend on the concentration of IAA, ARF and of the heterodimer ARF–IAA, implementing the negative feedback in the system. (See electronic supplementary material for the explicit form of this function *h*.)

Lastly, let us briefly discuss the parameters of the type *δ*_*X*_ which indicate degradation rates. The degradation rate of ARF is a constant, *δ*_*A*_. By contrast, IAA is taken to be degraded whatever its form (free or bound) at a rate that depends on auxin concentration (cf. *δ*_*I*_(*x*), *δ*_*II*_(*x*), *δ*_*IA*_(*x*), where *x*=[*auxin*]). In addition, a spontaneous decay rate δ*_IX_, *X*=(*I*,*A*) for the dimers has also been taken into account. What is the form of the auxin dependence of degradation rates? It is known that the auxin-dependent degradation pathway is based on IAA and auxin jointly binding to a complex involving the auxin receptor TIR1, followed by ubiquitination of IAA and ending by IAA degradation in the proteosome. In the Vernoux model that whole pathway is summarized by one effective reaction which degrades IAA at a rate *δ*_*I*_(x) times the concentration of IAA. Specifically, Vernoux *et al.* take *δ*_*I*_(x) to be given by a Michaelis–Menten-like law:

3.6 $$\delta_{I}(x)=\gamma_{I}\delta_{I}\frac{Kx}{1+Kx}.$$

Those authors justified this form by using a quasi-steady-state approximation in the ‘perception module’ (the sub-network of molecular species directly coupled to auxin) based on the assumption that ubiquitination and degradation of IAA occur on slower timescales than the formation of the auxin–IAA–TIR1 complex which catalyses ubiquitination. They also neglected the concentration of the triple complex auxin–TIR1–IAA compared with that of auxin–TIR1. (In §4, we shall rediscuss this last approximation because it will turn out to be unjustified.) The Vernoux model furthermore takes the auxin-dependent decay of IAA molecules to arise whatever the form of IAA. As a result, IAA_2_ (respectively, ARF–IAA) is degraded at a rate *δ*_*II*_(*x*) [IAA_2_] (respectively, *δ*_*IA*_(*x*) [ARF–IAA]). In their default parametrization as given in BioModels, Vernoux *et al.* [16] set *δ*_*I*_(*x*), *δ*_*IA*_(*x*) and *δ*_*II*_(*x*) to have the same functional form. In all cases, *δ*_*I*_ (or *δ*_*II*_ or *δ*_*IA*_) can be thought of as the basal decay rate of IAA in the considered form, while *γ*_*I*_ sets the maximum fold increase in decay rate that can be induced by auxin (the model’s default parametrization has all these fold increases at identical values). Finally, *K* is the affinity of auxin to the TIR1 receptor.

**Figure 2.**
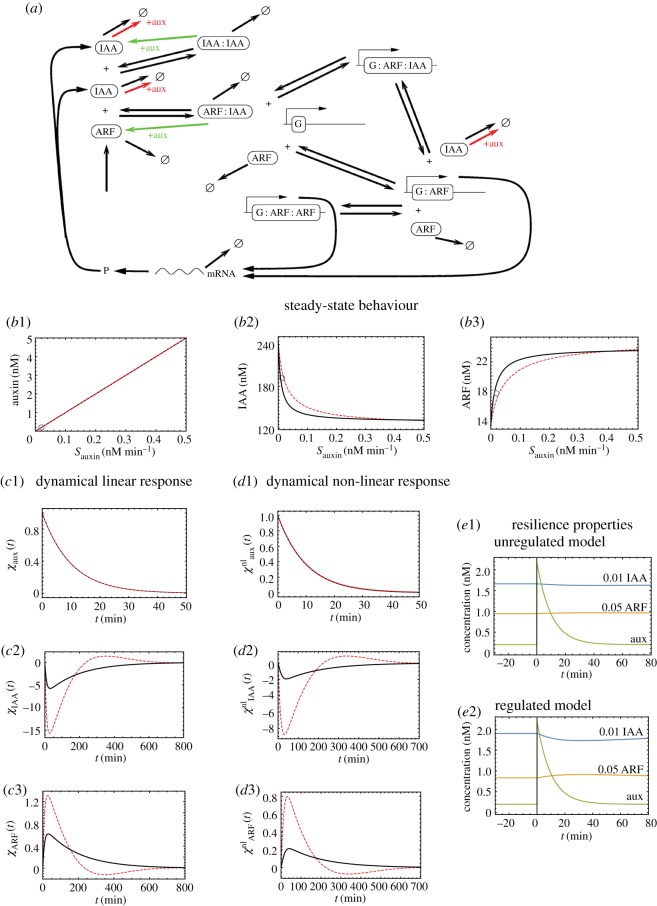
Analysis of the Vernoux model: steady-state behaviour, dynamical linear response and resilience. (*a*) Molecular species and processes in the Vernoux model (picture taken from SM of Vernoux *et al.* [16]). Symmetric arrows represent the reversible reactions between the different species; asymmetric arrows represent irreversible processes. *G* stands for a pool of genes responding to auxin, specifically here IAA. The active degradation of IAA in all of its forms by auxin is highlighted in red and green. (*b*1–*b*3) Steady-state concentrations as a function of the auxin influx *S*_*auxin*_ for auxin (*b*1), IAA (*b*2) and ARF (*b*3) in the regulated (dashed red) and unregulated (full black) cases. Parameters for the regulated model are taken from [16], namely: $k_{\mathrm{IX}}=1\;{\left(\mathrm{nM}\;\min\right)}^{-1}$, *K*_*IX*_=10 *nM*, $\delta_{I}=0.05\;\min^{-1}$, $\delta_{A}=0.003\;\min^{-1}$, $\delta_{R}=0.007\;\min^{-1}$, $\delta_{\mathrm{IX}}=0.003\;\min^{-1}$, $\delta_{\mathrm{IX}}^{*}=0.003\;\min^{-1}$ (with *X*=I,A), $\pi_{I}=1\;\min^{-1}$, $\pi_{A}=1\;\mathrm{nM}\;\min^{-1}$, *f*, *fA*=10, *ω*_*A*_, *ω*_*I*_, *ω*_*D*_=10, *γ*_*I*_=10, *K*=1 *nM*^−1^, *B*_*d*_=100 *nM*, *K*^−^_*A*_=10, $k_{\mathrm{IX}}^{\prime }=K_{\mathrm{IX}}k_{\mathrm{IX}}=10\;\min^{-1}$. In our extension, $\tau_{\mathrm{auxin}}=10\;\min$. For the unregulated version of the model, we have set *δ*_*I*_=2.1, *K*=5.5 *nM*^−1^ and set the IAA transcription rate to its value at very low auxin. (*c*1–*c*3) Dynamical linear response functions for auxin (*c*1), IAA (*c*2) and ARF (*c*3) in the regulated (red) and unregulated (black) cases. Parameters are as in panel *b*1–*b*3 and we have set $S_{\mathrm{auxin}}=0.02\;\mathrm{nM}\;\min^{-1}$. The associated steady-state values for auxin, IAA and ARF concentrations are marked with a circle in panels *b*1–*b*3. (*d*1–*d*3) The dynamical nonlinear response functions for auxin (*d*1), IAA (*d*2) and ARF (*d*3) in the unregulated and regulated versions of the Vernoux model, with parameters and *S*_*auxin*_ as above. These describe the response of the system to an additive perturbation in auxin equivalent to 10 times its steady-state value. The corresponding time courses are plotted in *e*1 (unregulated model) and *e*2 (regulated model); we have multiplied IAA and ARF by prefactors (respectively, 0.01 and 0.05) to display all curves using the same scale.

Figure 2*a* shows the network structure in the Vernoux *et al.* model [16]. At a qualitative level, the behaviour of the molecular network can be understood as follows. In the absence of auxin, ARF is sequestered in the ARF–IAA heterodimer. When auxin levels are increased, IAA is rapidly degraded, freeing up ARF which can then drive downstream targets (these are not part of the model). Our aim here is to revisit this model to reveal the role of the negative feedback affecting IAA transcription following the same logic as for our minimal model. To do so, we first added a reaction to make auxin concentration a dynamical quantity:

3.7 $$\frac{d[\mathrm{auxin}]}{dt}=S_{\mathrm{auxin}}-\frac{\left[\mathrm{auxin}\right]}{\tau_{\mathrm{auxin}}}.$$

With these additional dynamics, the auxin influx *S*_*auxin*_ becomes our control parameter; we set the auxin lifetime *τ*_*auxin*_ to 10 min based on the interval of values estimated by Band *et al.* [20]. Note that equation (3.7) extends the Vernoux model but in practice this extension is purely notational if one focuses on the steady-state regime. Indeed, in that situation the Vernoux parameter [auxin] is the same as the product *S*_*auxin*_*τ*_*auxin*_ of our extension. Furthermore, in the time-dependent regime, the shapes of the response curves are insensitive to the detailed value of *τ*_*auxin*_ because it is much smaller than the other characteristic times. In view of these properties, from here on the term ‘Vernoux model’ will refer to our extension using equation (3.7). Figure 2*b*1–*b*3 shows the steady-state concentrations of auxin, IAA and ARF as a function of *S*_*auxin*_. The three curves are monotonic as expected. Note that the values of the steady states of [IAA] and of [ARF] lie in a range such that the ratio of the maximum to the minimum is small (see caption of figure 2). Such a surprisingly small ratio can be traced to the default settings in the Vernoux model and specifically to the Michaelis–Menten equations describing the ubiquitination processes which saturate early, preventing further degradation of IAA.

### 3.2. Role of the negative feedback

In this subsection, we compare the behaviour of the system with and without regulation. The main change with respect to what was accomplished in §2 is that, in the presence of *arbitrary* regulatory dynamics, it is generally not possible to enforce *identical* steady states when regulation is removed. Fortunately, that is not a major problem here because it is possible to have the steady states in the two cases be nevertheless quite similar. Specifically, to define an unregulated version of the Vernoux model, we have (i) set the rate of IAA transcription to its basal value (arising when there is very low auxin) and (ii) compensated this low rate of IAA production by lowering the rate of IAA degradation (cf. caption of figure 2). With this choice, the steady-state curves are very similar with and without regulation as shown in figure 2*b*1–*b*3. Note that by construction they coincide for *S*_*auxin*_≃0 and that their relative differences throughout the whole range of *S*_*auxin*_ are less than 10%.

Given that these two models have very similar steady-state behaviours, we are in a position to test whether regulation is important for the dynamical responses, using the same logic as was used in the minimal model. The Jacobians giving the linearized dynamics about the steady state in each case are easily computed. (The mathematics follows directly the steps used in the minimal model; see electronic supplementary material, §SII). As expected, all eigenvalues have negative real parts, demonstrating that the steady states are stable. In figure 2*c*1–*c*3, we display the linear dynamical response functions for auxin, IAA and ARF. We see that the case with regulation has significantly larger responses to auxin perturbations. The negative feedback loop thus allows the system to be far more sensitive than in the absence of this regulation. This conclusion extends to the nonlinear regime as illustrated in figure 2*d*1–*d*3. We defined the nonlinear response function as the response divided by the size of the auxin impulse so as to have the (nonlinear) response per unit of extra auxin. The nonlinear response for auxin is the same as the linear one because the equations for auxin are linear. However, this is not the case for IAA and ARF. For these panels, we added into the system auxin in an amount 10 times its steady-state value. The response per unit of auxin is lower than in the linear regime, but we still clearly see the enhancement when comparing presence versus absence of regulation. For these plots, we use the default parameters of the Vernoux model and for that choice the responses, e.g. at the level of ARF, are particularly small. Larger and thus more realistic responses can be obtained, for instance, by increasing the auxin-dependent decay rates while still keeping the conclusion that the negative feedback allows for enhanced responses compared with the case without feedback.

Lastly, we can ask whether the much higher sensitivity of the regulated model might be associated with low *resilience*. The answer is no: there are large responses to perturbations but nevertheless the system returns *faster* to the steady state as illustrated in figure 2*e*1,*e*2. To provide a quantitative measure of this, we determined the time *δ*T required for the linear response function to come back down to 10% of its maximum value for IAA or ARF. We find that these times are systematically shorter with regulation than without. Such resilience also characterizes the system beyond the linear regime (cf. electronic supplementary material, tab. S1 for comparing the linear and nonlinear cases).

## 4. Results when building a new calibrated model of auxin signalling

The study of mathematical models of auxin signalling is useful for characterizing how various actors in the network contribute to auxin responses, clarifying in particular the role of different molecular species. To our knowledge, the work of Vernoux *et al.* [16] is the only one to provide an associated *calibrated* model including all the processes previously mentioned. Other models of auxin signalling have been published [15,17] but the corresponding studies have focused on qualitative aspects, to a large extent because the numerous parameters arising in these mathematical models are largely unknown. In retrospect, one may then ask what is to be gained by modifying the Vernoux model. Our answer is that improvements can be obtained at the following levels: (i) the rather complicated form of the transcription rate in the Vernoux model (cf. electronic supplementary material, eq. S25) has its origin in the assumption that two ARF molecules may bind cooperatively to the regulatory region upstream of the IAA gene. But we find no evidence in favour of this assumption in *Arabidopsis*, as will be explained in the next subsection. Thus, dropping this assumption both simplifies the model and we believe provides for greater realism. (ii) We mentioned in §3 that equation (3.6) was derived by neglecting the concentration of the triple complex auxin–TIR1–IAA relative to that of auxin-TIR1 (cf. SM of Vernoux *et al.* [16]). Vernoux *et al.* justified this simplification by assuming that [IAA] was low. However, when examining the Vernoux model *a posteriori* with its calibration, we can see that [IAA] never goes to small values (cf. figure 2*b*2). Furthermore, experimental work shows that auxin does not bind to TIR1 without the presence of IAA, suggesting that the triple complex auxin–TIR1–IAA occurs in concentrations much larger than that of the double complex auxin–TIR1. (iii) We mentioned that the dynamical range of ARF and IAA in the Vernoux model is modest. Indeed, a factor 2 range seems biologically unrealistic, and furthermore the responses to strong auxin perturbations (cf. figure 2*e*) are small.

Given such limitations in the Vernoux model, we were compelled to build a new model via the following steps: (i) we consider all the processes included in the previous mathematical models [15–17]; (ii) we selectively discard some of the processes after appropriate justifications; (iii) we calibrate the resulting model using previous work [16,20] and physico-chemical constraints, ensuring that the previously mentioned limitations of the Vernoux model are overcome.

### 4.1. Model building and model choices

We begin with the union of the molecular species and processes proposed in the previous models as given in [15–17]. These models do not distinguish the different members of the ARF or IAA families. We stay within that simplified framework as in the previous sections we showed it was sufficient to reveal a role for the negative feedback. The framework of these models of auxin signalling introduces two main modules. The first senses the presence of auxin and leads to the degradation of IAA. The corresponding molecular species are auxin, TIR1 and IAA. The processes are the influx of auxin, the formation and dissociation of the complexes auxin–TIR1 and auxin–TIR1–IAA, the ubiquitination of IAA in that triple complex and the degradation of that ubiquitinated IAA [15]. The concentration of auxin is increased by an incoming (extracellular) flux and is decreased by a spontaneous degradation; by contrast, TIR1 is taken as neither produced nor degraded. The second module is centred on IAA and ARF. The corresponding molecular actors are IAA in its monomer form, IAA in its homodimer form, ARF in its monomer form, ARF in its homodimer form and the ARF–IAA heterodimer. The main processes are the formation and dissociation of these dimers. To control transcription of IAA messengers, there is binding and unbinding of transcription factors to the dedicated DNA-binding sites upstream of the IAA gene, sites called auxin response elements, abbreviated hereafter as AuxREs. The binding and unbinding to AuxREs are taken to be fast so that one may apply equilibrium thermodynamics (using the instantaneous concentrations) to obtain the probabilities of occupation of the regulatory region by the different transcription factors [15]. For each possibility for the state of this regulatory region (free, bound to ARF, bound to ARF-IAA, etc.), there is an associated transcription rate. The other processes tied to IAA are the degradation of IAA messenger RNA (a simple decay due to a finite lifetime) and the translation of the IAA messenger RNA which produces IAA protein.

**Figure 3.**
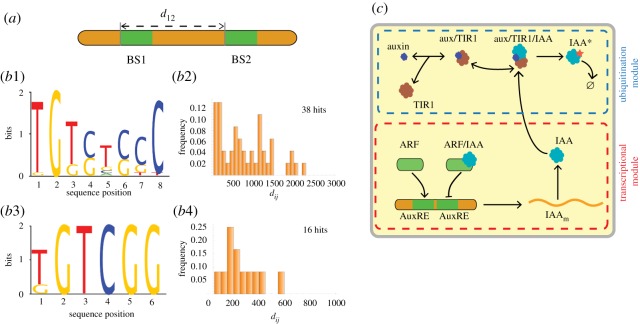
Lack of evidence for a transcriptional control by ARF homodimers and schematics of the FLAIR model. (*a*) Schematics of the search for pairs of AuxRE sequences. (*b*1,*b*2) The position weight matrix given in [22] for ARF1 and ARF5 binding sites and its corresponding frequencies of distances *d*_*ij*_ between adjacent hits in the genomic regions upstream of the 21 IAA genes of *Arabidopsis* (see electronic supplementary material for further details). (*b*3,*b*4) Same as in *b*1,*b*2 but using the position weight matrix given in [23]. (*c*) Representation of the molecular network and processes in our new model FLAIR. For pedagogical reasons, we display two AuxREs rather than just one so that the reader can understand the logic of the panels (*a*) and (*b*). Arrows represent reactions between the different species. The signalling (ubiquitination) and transcriptional modules are also highlighted. The question is whether the ARF homodimer might affect transcription by binding to two close-by transcription-factor binding sites (AuxREs).

Let us now discuss our model choices in relation to the assumptions of previous works. Both of the works [15,17] take ARF to be neither produced nor degraded. The Vernoux model [16] allows for spontaneous decay of ARF but for our model we will take ARF to be conserved as in [15,17]. It is necessary to justify a bit the imposition of constant total amounts of TIR1 and ARF. This type of constraint is appropriate if two conditions are met. First, these proteins should be long-lived, that is they should not be much degraded on the timescale on which one will investigate the properties of the model. Under such conditions, the total amount of either TIR1 or ARF will not have time to change significantly during the timescale of the experiment. Second, it is necessary that this total amount of protein (either TIR1 or ARF) not depend on the intensity of the influx of auxin. As little is known about such putative effects, we shall accept to keep TIR1 and ARF at fixed total concentrations hereafter. A second choice imposed by the authors of [15,17] is for IAA to be degraded solely by the ubiquitination pathway (no spontaneous decay in the absence of auxin). That property, namely an infinite lifetime of IAA in the absence of auxin, together with a non-zero transcription rate of IAA, leads to the undesirable behaviour that IAA concentrations become huge as auxin influx is tuned down. For the model to have a sensible behaviour even when auxin influx is very low, it is necessary to include a lifetime for IAA protein and this then leads to one process in our model which was absent in [15,17]. A third choice made specifically in [16,17] was to allow IAA to homodimerize. This seems justified given that IAA homodimerization has been demonstrated *in vitro*. However, the dissociation constant of IAA homodimer is higher than that of ARF homodimer [21]. Furthermore, allowing IAA homodimerization will reduce the network’s response to auxin signalling, as we show in electronic supplementary material, §SII, so we have decided to discard that process from our model. Our fourth modification to the models [15–17] concerns the control of IAA transcription. Authors of those previous models took the transcription rate to be affected by the binding to the regulatory region of an ARF–IAA heterodimer (a choice we shall keep) but also by the binding of ARF in both its monomer and homodimer form. If transcription were modulated by ARF in its homodimer form, we would expect the AuxREs to arise in close-by pairs. We have performed a search for such pairs in the regulatory regions upstream of 21 IAA genes of *Arabidopsis thaliana* (see electronic supplementary material, §SIII). These bioinformatic scans were based on the position weight matrices published in two previous works characterizing AuxREs [22,23] and illustrated in figure 3*a*,*b*1–*b*3. Also shown there are the histograms constructed from the distances of the adjacent AuxREs found from our scans (cf. electronic supplementary material, tab. S2 for our raw results) obtained with the aid of the software MOODS [24]. These histograms have no sign of enrichment at very short distances, suggesting that AuxREs do not come in close-by pairs in the case of IAA regulatory regions (cf. figure 3*b*2–*b*4). Given this conclusion, we modified the transcriptional regulation proposed in [15–17], removing the terms associated with ARF acting as a homodimer.

Based on the changes just exposed, the molecular actors and set of processes kept in our model are represented in figure 3*c*. For pedagogical reasons, to go with figure 3*a* and figure 3*b*1–*b*4,*c*, figure 3*c* is represented with two AuxREs even though our new calibrated model assumes only one such binding site. Lastly, the forms of the differential equations specifying the dynamics of each species in our model are determined quite directly from the use of mass action for all elementary reactions and the choices specified above. These equations are given in electronic supplementary material, §SIII and can be retrieved electronically from the Biomodels repository (model FLAIR for ‘Feedback Loop in ARF and IAA Response’, no. 1706070000).

### 4.2. Calibrating the model

The study of the behaviour of our model requires defining the values of all parameters in the equations. Ensuring that these values are realistic is difficult because of the dearth of quantitative experimental measurements. To calibrate our model, we thus combined different strategies and bibliographic sources allowing *in fine* to set the 17 parameters. First, the degradation rates of the IAA protein via ubiquitination and the lifetimes of IAA mRNA have been estimated experimentally (cf. [25] and references in [17]). Second, we exploited the fact that, in this kind of system, transcription factor concentrations are expected to be in the range of 1–100 nM. Concerning the association and dissociation rates for the different dimers, we relied on measurements of the dissociation constants *K*_*D*_ of homo- and heterodimers in the cases of IAA17 and ARF5 [21]. Note that such dissociation constants are expressed in terms of the on and off rates, *K*_*D*_=*k*_*off*_/*k*_*on*_. To determine *k*_*on*_, we made use of physico-chemical theory. Specifically, two diffusing monomers need to collide for the dimerization reaction to proceed. Such a situation is usually addressed as a *diffusion limited reaction* and it puts an upper bound on the *k*_*on*_ coefficient (see electronic supplementary material, §SIII). We have done so for the different mass action reactions in our network, leading to bounds on all the corresponding *k*_*on*_ parameters. Once a value has been decided for *k*_*on*_, knowing *K*_*D*_ determines *k*_*off*_. Lastly, we exploited parameter estimates in [20] where IAA ubiquitination kinetics was studied using fluorescent reporters. That work provides plausibility intervals for its parameter values, allowing us to specify some of our values using their midpoints in the absence of other information. Owing to these combined strategies, we set the values of all 17 parameters of our model; the results are reported in electronic supplementary material, tab. S3.

### 4.3. Steady-state and dynamical properties within the model itself

**Figure 4.**
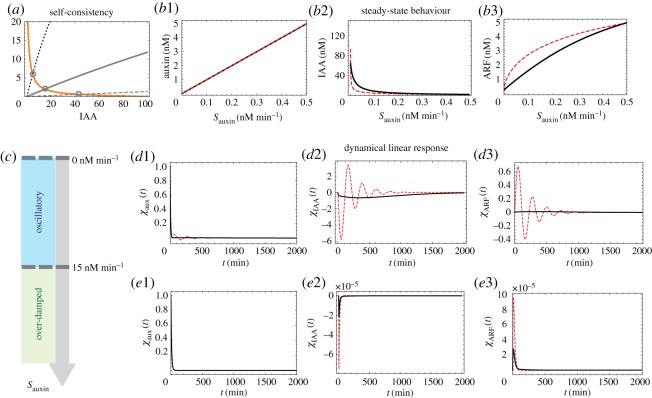
Steady states and dynamical response functions in the FLAIR model. All parameters are fixed as in electronic supplementary material, tab. S3. (*a*) Graphical representation of the self-consistent equation determining IAA concentration in the steady state (here *S*_*auxin*_=0.001, 0.01 and $0.1\;\mathrm{nM}\;\min^{-1}$). (*b*1–*b*3) Steady-state concentrations as a function of *S*_*auxin*_ for auxin, IAA and ARF for the regulated (red dashed line) and the unregulated model (black thick lines). (*c*) The two regimes for the system’s dynamical behaviour as a function of increasing auxin influx, *S*_*auxin*_: for low auxin influx, the behaviour is strongly oscillatory; at high auxin flux, it is over-damped. (*d*1–*d*3) Dynamical linear response functions for the three species, auxin, IAA and ARF, in the regime of small auxin influx where the negative feedback leads to oscillations (red dashed lines). Here, $S_{\mathrm{auxin}}=0.02\;\mathrm{nM}\;\min^{-1}$. (*e*1–*e*3) Same as *d*1–*d*3 but for $S_{\mathrm{auxin}}=50\;\mathrm{nM}\;\min^{-1}$ in which case the behaviour is relaxational (no imaginary parts to the eigenvalues of the Jacobian), but nevertheless exhibits overshoots.

Our analysis of the FLAIR model follows the same logic as used in the previous models. The determination of the steady states in the network proceeds by deriving a self-consistent equation for the concentration of free IAA protein. The solution of this equation reduces to finding the intersection of two curves as illustrated in figure 4*a*. Once this steady-state concentration is determined, that of all the other molecular species follows (cf. details in electronic supplementary material, §SIII). Then, auxin, IAA and ARF steady-state concentrations as a function of *S*_*auxin*_ (cf. figure 4*b*1–*b*3) are seen to be qualitatively the same as in the previous models.

Consider now the dynamical behaviour of the network after subjecting its steady state to a perturbation in auxin concentration. As before, one can compute the Jacobian of the system, here a 10×10 matrix. Its eigenvalues give the stability of the steady states as well as their behaviour when subject to an instantaneous auxin perturbation. The steady-state solutions of the network are stable for any auxin influx. More interestingly, in the regime of small auxin influx, an oscillatory behaviour can be observed due to the presence of complex eigenvalues just as occurred in the Vernoux model. In the FLAIR model, we observe that as auxin influx is ramped up, the oscillations are more and more damped until they altogether disappear. At that point, the Jacobian *J* has all of its eigenvalues real and negative. As expected, the amplitudes of the linear response functions decrease with increasing *S*_*auxin*_. There are thus two regimes. In the first, arising at low auxin influx, one has a high sensitivity to auxin perturbations and the relaxation back to the steady state occurs via damped oscillatory behaviour (figure 4*c*,*d*1–*d*3). In the second, arising at high auxin influx, the system is relatively unresponsive to auxin perturbations (small amplitudes of the response function) and relaxation back to the steady state occurs without oscillations (figure 4*c*,*e*1–*e*3). Nevertheless, this over-damped relaxation allows for the presence of overshoots. For completeness, we have also provided the system’s behaviour when removing regulation as we did for the Vernoux model. As for that case, a rescaling of the rate for ubiquitination allows to compensate most of the effects of regulation when considering only the steady-state behaviour. Within that framework, when removing the negative feedback on IAA transcription, we see from figure 4*d*1–*d*3,*e*1–*e*3 that (i) the dynamical response is much reduced and (ii) there are no more oscillations.

## 5. Results for downstream targets in our FLAIR model

Our results so far show that the presence of a negative feedback enhances the dynamical responses in auxin signalling. Furthermore, the negative feedback increases the resilience of the network, which is able to recover its initial state faster in the presence of a negative feedback. Given these findings, one might wonder what biological benefits these features could have beyond the core network considered so far. To answer this question, we considered two different kinds of ARF targets for which these ARF response amplifications might turn out to be beneficial: (i) a stress–response gene and (ii) a developmental-switch gene. We study these questions using our FLAIR model.

### 5.1. Dynamics of downstream targets: case of a stress–response gene

Our first focus is on a stress–response gene that operates downstream from the signalling network. Call this ‘effector’ *E* and assume it is driven (activated) by ARF monomer or ARF homodimer. For simplicity, we consider that the influence is unidirectional so that the abundance of *E* has no influence on the state of the auxin signalling network and in particular on ARF. Assuming one starts the system in the steady state, let signalling be implemented by having *S*_*auxin*_ be increased *n*-fold during a time window of duration t_*c*_. In the framework of our new calibrated model, this perturbation will lead to a time dependence of all the molecular species. The behaviour in time of [*E*] is then determined by the time-dependent functions [ARF](*t*) and [ARF_2_](*t*). Is there any importance in having the effector be driven by concentrations of ARF monomer versus of ARF homodimer? To investigate this, for specificity we lump together transcription and translation of *E* and then use dynamical equations for its abundance. In this subsection, we take *E* to be a stress–response gene so that its actions are transient if the stimulus (auxin) is transient. If *E* is controlled by ARF monomer, we thus set

5.1 $$\frac{d[E]}{dt}=\alpha \frac{\left[\mathrm{ARF}\right]}{\beta +[\mathrm{ARF}]}-\delta [E],$$

while if it is controlled by ARF homodimer, we set

5.2 $$\frac{d[E]}{dt}=\alpha \frac{\left[{\mathrm{ARF}}_{2}\right]}{\beta^{2}+[{\mathrm{ARF}}_{2}]}-\delta [E].$$

**Figure 5.**
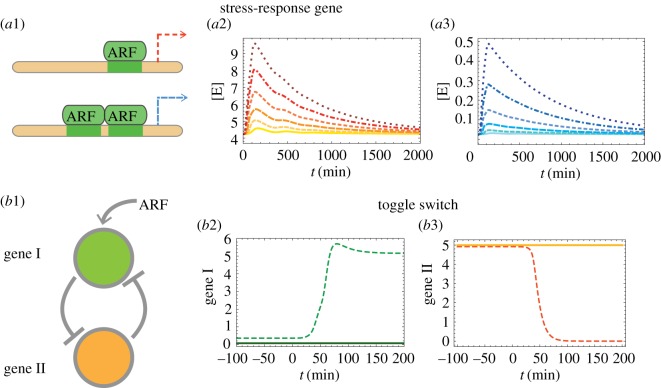
Responses to auxin signalling based on the FLAIR model: cases of genes downstream from the main network that are driven by ARF. (*a*1) Case of a stress–response gene, with synthesis induced by ARF monomer (cf. equation (5.1)) or ARF homodimer (cf. equation (5.2)), (*a*2,*a*3) in the FLAIR model (with the negative feedback). Depicted are the time-dependent responses of the downstream gene after perturbing auxin influx within a time window, increasing the amplitude of this influx by factors 2, 4, 8, 16, 32 and 64 (shown in the panels using lighter to darker colours). In the monomer case, the increase of the response with perturbation intensity is linear only for very small perturbations; for the regime shown it is roughly logarithmic. By contrast, for the homodimer case, the increase with perturbation intensity is superlinear, with saturation effects setting in much later than in the monomer case. (Basal rate $S_{\mathrm{auxin}}=0.02\;\mathrm{nM}\;\min^{-1}$ with perturbation applied for $t_{c}=100\;\min$, $\alpha =1\;\mathrm{nM}\;\min^{-1}$, *β*=15 *nM* and $\delta =0.0013\;\min^{-1}$.) (*b*) Case of a developmental-switch gene pair where each gene inhibits the other and gene I is in addition driven by ARF monomer (cf. equations (5.3) and (5.4)). We show the time-dependent responses of the two genes (panels *b*2 and *b*3) when auxin influx is instantly increased by a factor 2. Each panel displays the behaviour when using our new calibrated model with and without regulation (respectively, dashed and solid lines), showing that switching is facilitated by the negative feedback in the auxin signalling network. (Basal rate $S_{\mathrm{auxin}}=0.02\;\mathrm{nM}\;\min^{-1}$ and stepwise window perturbation doubling *S*_*auxin*_ for $t_{c}=50\;\min$.) Toggle parameters are given in electronic supplementary material, tab. S3.

Qualitatively, the auxin stimulus will lead to a temporary excess synthesis of *E*. The lifetime of *E* will determine the characteristic time during which the enhanced concentration of *E* will last. This time thus has little to do with the time of the auxin perturbation. Our interest lies more in the nonlinearity of the system, especially when comparing the two types of control (monomer versus dimer) on *E*. In figure 5*a*1, we have sketched the two cases, one where the ARF monomer controls synthesis and one where it is the ARF homodimer. Figure 5*a*2 shows the time-dependent concentration of *E* based on equation (5.1). To examine the dependence on the strength of the perturbation, we have instantaneously increased auxin influx by the factors 2, 4, 8, 16, 32 and 64. From the curves, we see that *E* rises and then decays (on the scale of the lifetime of *E*) and that the curve heights grow with the perturbation size. However, this growth is far from linear: it is much slower, close to logarithmic, indicating that the linear regime occurs only for quite small perturbations. Analogously, figure 5*a*3 shows the time-dependent concentration of *E* based on equation (5.2). Using the same perturbations, one can see by examining the amplitudes reached by the curves that for modest perturbations the response is definitely nonlinear. The reason is that the driver itself ([ARF_2_]) is nonlinear. Another point of interest is that the fold increases are far larger than when the driver is ARF. This behaviour indicates that the *cooperative* effects associated with exploiting ARF in its dimer form can lead to responses downstream which are closer to that of the go–no-go type. This qualitative behaviour could be further enhanced by allowing for multiple dimers to bind.

### 5.2. Dynamics of downstream targets: case of a developmental-switch gene

Auxin signals also induce changes in developmental programs *via* the dynamics of ARFs. Examples include the organogenesis of flowers or of roots (see, for instance, the review [26]). ARFs are also known to drive many growth processes such as elongation or gravitropism. Because the field is quite new, only a few genes have been shown to be direct targets of ARF [27,28]. As developmental programs are generally thought of as near-irreversible switches, we shall consider here the putative case in which ARF drives a downstream target belonging to a genetic switch. For specificity, we will assume that the (temporary) enhancement of ARF has the consequence of turning the switch or ‘toggle’ from off to on.

Toggle switches have been studied within many systems but quantitative modelling has been limited essentially to unicellular organisms. To stay close to such a quantitative case and avoid introducing arbitrary dynamics, we use the mathematical framework given in [29] which involves two mutually inhibitory genes as indicated in figure 5*b*1. In the reference state, gene I is off, while gene II is on and maintains gene I off. But with a high enough stimulus (concentration of ARF monomer), gene I can be induced to sufficiently high levels so that there is a switch. For simplicity, we again consider that there is no feedback from these genes back onto ARF or the auxin signalling network. The toggle switch in [29] specifies the dynamics of the concentrations of the genes I and II after lumping together transcription and translation. Allowing for gene I to be directly induced by ARF, one then has the following differential equations:

5.3 $$\frac{d[g_{1}]}{dt}=t_{\mathrm{ARF}}\frac{\left[\mathrm{ARF}\right]}{K_{\mathrm{ARF}}+[\mathrm{ARF}]}+\frac{\alpha_{1}}{1+{\left[g_{2}\right]}^{\beta }}-\delta_{1}[g_{1}]$$

and

5.4 $$\frac{d[g_{2}]}{dt}=\frac{\alpha_{2}}{1+{\left[g_{1}\right]}^{\gamma }}-\delta_{2}[g_{2}].$$

These Hill equations involve exponents *β* and *γ* [29], *α*_1_ and *α*_2_ are base production rates, *δ*_1_ and *δ*_2_ are spontaneous decay rates and *K*_*ARF*_ is a Michaelis–Menten constant. Given this toggle module, figure 5*b*2,*b*3 show the time-dependent concentration of the two genes based on equations (5.3) and (5.4) when an auxin pulse is injected into the system using the same protocol as in figure 5*a*2,*a*3 (dotted curves). For comparison, we also show the case where the negative feedback in our model has been removed (solid curves). Because the negative feedback of IAA allows a significantly larger dynamical response to auxin, the downstream toggle indeed performs its switch when it is driven with the feedback, but in the absence of feedback the ARF signal is weak and so the toggle just returns to its initial state after a brief time.

## 6. Conclusion

Fast responses to stress are crucial for plant survival. It is, therefore, no surprise that the auxin signalling systems in plants involve biochemical processes which release ARFs almost immediately upon receipt of a signal, bypassing the need for synthesis of ARFs via transcription or translation. Operationally, when there is little auxin, the molecular network sequesters ARF in the form of ARF–IAA heterodimers. By contrast, at high concentrations of auxin, the ubiquitination-dependent degradation of IAA (which is much faster than ARF synthesis) frees up ARF that can then drive its downstream targets. Naturally, it is necessary to regenerate IAA but this requires longer timescales because it involves transcription and translation. In plants where it has been studied, this regeneration involves a feedback wherein IAA acts negatively on its own transcription. As a result, an auxin stimulus rapidly leads to both IAA depletion and enhanced transcription of IAA messengers. Owing to modelling and computational analysis, we can examine which qualitative and quantitative features are rendered possible by such a negative feedback. We find that two quite striking properties emerge at the level of the system’s responses to auxin perturbations: (i) responses have much greater amplitudes, and (ii) the system is more resilient, returning to its native state more quickly after the transient auxin signal is gone.

The first of these two properties is not particularly intuitive, but our strategy was to introduce a comparison between networks in which the steady-state behaviours are imposed to be similar or identical whether there is or not the negative feedback (cf. for instance figures 1*b*1–*b*3 and 2*b*1–*b*3). Then, by using in particular the *dynamical linear response function*, we find that the key factors driving a large response are the rates within the ubiquitination pathway. The second of the two properties is more intuitive. If a system is perturbed, then *reacting against* that disturbance will generally lead to greater resilience. In our specific auxin signalling system, the negative feedback does just that, sometimes leading to an overshoot (cf. figures 2*c*,*d* and 4*e*). What is appealing is that this enhanced resilience occurs in spite of having an initial response which drives the system much further away from the steady state (cf. figure 2*e*1,*e*2). Put together, one may say that the auxin signalling network has an architecture or is governed by an operating principle which ensures the two *a priori* antagonistic properties: (i) great dynamical sensitivity to signals and (ii) high resilience.

It is tempting to extrapolate and expect such an operating principle to be at work in other signalling systems. Interestingly, negative feedbacks are in fact omnipresent in plant signalling pathways [30]. A good example is gibberellin-dependent signalling in which gibberellin is analogous to auxin for our system. In that gibberellin pathway, DELLA is analogous to IAA: it is degraded via the (gibberellin-dependent) ubiquitination pathway and it inhibits its own transcription. Similarly, PIF3 and PIF4 are transcription factors analogous to ARF, they are released by degradation of DELLA and they drive downstream effectors [31]. This gibberellin system in fact has *multiple* feedback loops, making it difficult to display a single operating principle. Certainly, the identification of ‘the role’ of one feedback does not necessarily reflect reality when dealing with complex networks where assigning a function to a process or module is an anthropomorphic projection. Nevertheless, having a particular component (process, feedback loop, etc.) in a network can enrich the possible behaviours beyond what is possible without it, allowing one to assign at least partly a role to that component. The difficulty is to show that these additional properties are unlikely to be achieved instead by simple parameter tuning of the other components. In our auxin signalling network, that difficulty was overcome by constraining the steady-state properties to be nearly feedback-independent. Given that constraint on the statics, it is natural to then infer a role for feedback at the level of the system’s dynamical behaviour as we do.

Given our insights into possible roles of components in the Vernoux signalling network model, be they feedback control or dimerization, we propose a new fully calibrated model called FLAIR (for Feedback Loop in ARF and IAA Response) with some qualitative (in addition to quantitative) changes compared to the Vernoux model. For instance, this new model does not include the IAA homodimer, and its transcriptional control of IAA does not involve ARF homodimer. Furthermore, it takes into account the dynamics of auxin and TIR1 for the ubiquitination of IAA. This extension should allow for using the model in situations where TIR1 is mutated or has its expression level modified. In building this FLAIR model, we also enforce that the ubiquitination machinery does not become saturated easily, leading to a much larger dynamic range for the abundance of free ARF in the system compared to what arises in [16]. We also saw that this FLAIR model could have quite large oscillations upon being stimulated by auxin (see, for instance, figure 4*d*2,*d*3). Interestingly, oscillatory behaviour in the expression of IAA has been seen experimentally in the case of the tip of roots [5] and arises also in the model of Middleton *et al.* [15]. Whether such oscillatory behaviour has a biological function or is simply the signature of high signalling sensitivity as happens in our FLAIR model remains to be seen.

Lastly, the auxin signalling pathway (with the components as listed in our new model, including the ubiquitination pathway) can be implemented in cellular systems such as yeast [32,33]. The possibility of using gene editing to introduce whichever IAA, ARF or TIR1 genes one wants from their respective families, along with reporters responding to the different ARFs, opens up the perspective of revealing new operating principles in these complex biomolecular networks. Will redundancy, cross-talk or cooperativity play central roles? The goal of understanding the operating principles behind plants’ incredibly diverse responses to auxin—dependent on the many members of the IAA, ARF and TIR1 families—seems to be within reach.

## Supplementary Material

Supplementary Text

## Data accessibility

All data used in this paper are available on online repositories as described in the electronic supplementary material, S1.
