# Supplementary material for: Responses to auxin signals: an operating principle for dynamical sensitivity yet high resilience
Source: R Soc Open Sci. 2018 Jan 24;5(1):172098. doi: 10.1098/rsos.172098 (PMC5792956; doi:10.1098/rsos.172098)

Supplementary Material to:
Responses to auxin signals: an operating principle for dynamical sensitivity yet high resilience

S. Grigolon*
The Francis Crick Institute, 1, Midland Road, Kings Cross, London NW1 1AT, United Kingdom

B. Bravi
Department of Mathematics, King's College London,
The Strand, London WC2R 2LS, United Kingdom,
Current affiliation: Institute of Theoretical Physics,
École Polytechnique Fédérale de Lausanne (EPFL), CH-1015 Lausanne, Switzerland

O.C. Martin†
GQE-Le Moulon, INRA, Univ. Paris-Sud, CNRS, AgroParisTech,
Université Paris-Saclay, 91190 Gif-sur-Yvette, France

I. PROPERTIES OF THE MINIMAL MODEL

The species and the dynamical equations of the minimal model were specified in the Main part of the paper (see Sect. 2). The interactions between the species in this model are based on simplified mass-action reactions. Specifically, the kinetic parameter α (respectively β) quantifies how the presence of auxin (respectively IAA) "degrades" IAA (respectively ARF) but this is an effective reaction which does not consume the active agent, here auxin (respectively IAA). This simplification, which enforces the feed-forward nature of the system, facilitates the mathematical analyses that we now present. Nevertheless, in the last sub-section of this part we shall show that our conclusions hold even if this simplification is dropped.

A. Steady states

In the steady state, the right hand sides of Eqs.1-3 are set to 0, leading to the following conditions on the steady-state concentrations:

$$[auxin]_{ss} = S_{auxin}\tau_{auxin}, \tag{S1}$$

$$[IAA]_{ss} = S_{IAA}/(\alpha\,[auxin]_{ss} + 1/\tau_{IAA}), \tag{S2}$$

$$[ARF]_{ss} = S_{ARF}/(\beta\,[IAA]_{ss} + 1/\tau_{ARF}), \tag{S3}$$

where S_{auxin}, S_{IAA} and S_{ARF} are necessarily time-independent and the "ss" subscript on the concentration of each species denotes that it is taken in the steady-state.

In the last equation, one may substitute $[IAA]_{ss}$ by its expression in terms of S_{auxin} or $[auxin]_{ss}$ if desired. If S_{IAA} is fixed (unregulated case), all the equations are explicit, showing that there is a unique steady-state solution. In the presence of regulation, S_{IAA} is not *a priori* known and must also be determined *via* the steady-state conditions.

B. Ensuring identical steady states for the regulated and unregulated minimal models

In the auxin signaling networks found in various organisms, there is a regulatory feedback [1], *i.e.*, S_{IAA} depends on $[IAA]$, $[ARF]$ and even on other molecular species. A hypothetical justification could be that feedback allows for

* silvia.grigolon@gmail.com
† olivier.martin@moulon.inra.fr

multi-stationarity, so Eqs. S1,S2,S3 could have more than one steady-state level of IAA and ARF for a given value of S_{auxin}. However that would require a *positive* feedback, whereas the feedbacks seen in plants correspond to negative retroactions, *i.e.*, when IAA is increased it *lowers* the value of S_{IAA}. As a result IAA is considered to act as a self-inhibitor. As mentioned in the main part of the article, even in the absence of regulation, it is possible to push the saturation curves in the steady state to large values of the input flux S_{auxin}. This can be seen by considering Eqs. S1-S3 in the unregulated model: IAA will become small (compared to its maximum value occurring when $S_{auxin} = 0$) when the incoming auxin flux becomes much larger than $S^*_{auxin} = 1/(\alpha\tau_{auxin}\tau_{IAA})$. By decreasing α, the range in S_{auxin} over which the steady state values of $[IAA]$ and $[ARF]$ avoid saturation can be increased at will, no regulatory feedback is necessary. But there is a drawback: any dynamical response will be weak whenever the coupling (α) is small.

What form of regulation will ensure both large α and that the steady-state curves saturate only for large S_{auxin}? To make the comparison between the two models (without and with regulation) as fair as possible, we shall impose their input-output relation curves (*cf.* Figures 1:B1-B3) to be the exactly the same. Consequently, they will have identical static properties, and in particular the static response functions will coincide for the two models. To mathematically define the model *with* regulation, we take its differential equations to be those of the model without regulation but introduce two exceptions. First, α in the case of regulation has a larger value, $\alpha^{(reg)} \gg \alpha^{(no-reg)}$. Second, the source term S_{IAA} for production of IAA is modified to compensate that change in α, enforcing the whole steady-state input-output relation to be the same in the regulated and unregulated models. Such a modification can be thought of as introducing a regulation in the production of IAA, for instance at the transcriptional level. There is still a lot of freedom for how to make such a regulatory change depend on different molecular species while maintaining exactly the same steady-state values. For both biological and mathematical reasons, we shall take S_{IAA} to be a function of only IAA, making IAA a (negative) self-regulator. Comparing the equations with and without regulation in the steady state, we set the *change* in IAA production to be equal to the *change* in IAA consumption, *i.e.*,

$$S_{IAA}^{(reg)} - S_{IAA}^{(no-reg)} = (\alpha^{(reg)} - \alpha^{(no-reg)})[auxin]_{ss}[IAA]_{ss}. \tag{S4}$$

This choice then ensures that Eq. S2 is satisfied and thus that the steady-state curves of Figures 1:B1-B3 are the same with and without regulation. However Eq. S4 gives $S_{IAA}^{(reg)}$ as a function of both $[auxin]_{ss}$ and $[IAA]_{ss}$. To make it only a function of $[IAA]_{ss}$, we replace $[auxin]_{ss}$ by its steady-state value when expressed in terms of the steady-state value of $[IAA]$ (*cf.* Eq. S2). We take this expression to be valid outside of the steady state, leading to:

$$S_{IAA}^{(reg)}([IAA]) = (\alpha^{(reg)}/\alpha^{(no-reg)})S_{IAA}^{(no-reg)} - (\alpha^{(reg)}/\alpha^{(no-reg)} - 1)[IAA]/\tau_{IAA}. \tag{S5}$$

This form indicates that at low $[IAA]$ (high auxin-influx rate) the IAA production rate in this regulated model is enhanced by a factor $\alpha^{(reg)}/\alpha^{(no-reg)}$ compared to the unregulated case. Inversely, when auxin influx vanishes, the production rate of IAA is the same in the regulated and unregulated cases; this could have been anticipated since without such influx, α plays no role so one must then have $S_{IAA}^{(reg)} = S_{IAA}^{(no-reg)}$. In addition, it is easy to see that because the production rate *decreases* as $[IAA]$ increases (as expected since IAA acts as a self-inhibitor), there is a unique solution to Eq. S2, and so the regulated system does not exhibit any multi-stationarity either. Lastly, plugging Eq. S5 into Eq. 2.2, we see that the regulated model is obtained from the unregulated model solely by multiplying the expression for $d[IAA]/dt$ by the overall factor $\alpha^{(reg)}/\alpha^{(no-reg)}$.

C. Stability of the steady states and effect of regulation

For a given value of S_{auxin}, the (unique) steady-state concentrations are determined by Eqs. S1-S3. Let us define $\overrightarrow{\Delta C}$ as the vector $\overrightarrow{\Delta C} = \{\Delta[auxin], \Delta[IAA], \Delta[ARF]\}$ whose components give the (time-dependent) deviations of the concentrations from their steady-state values. The linearization of the dynamics (Eqs.1-3) can be specified in matrix form:

$$\frac{d\overrightarrow{\Delta C(t)}}{dt} = \mathbf{J}\overrightarrow{\Delta C(t)}. \tag{S6}$$

In the absence of regulation, this Jacobian $\mathbf{J}$ is:

$$\mathbf{J}^{(non-reg)} = \begin{pmatrix} -\frac{1}{\tau_{auxin}} & 0 & 0 \\ -\alpha^{(no-reg)}[IAA]_{ss} & -\frac{1}{\tau_{IAA}} - \alpha^{(no-reg)}[auxin]_{ss} & 0 \\ 0 & -\beta[ARF]_{ss} & -\frac{1}{\tau_{ARF}} - \beta[IAA]_{ss} \end{pmatrix}. \tag{S7}$$

In these expressions, all concentrations are taken at their steady-state values. In the presence of regulation, the only change is that the second line is multiplied by the factor $\alpha^{(reg)}/\alpha^{(no-reg)}$:

$$\mathbf{J}^{(reg)} = \begin{pmatrix} -\frac{1}{\tau_{auxin}} & 0 & 0 \\ -\alpha^{(reg)}[IAA]_{ss} & -\frac{\alpha^{(reg)}}{\alpha^{(no-reg)}\tau_{IAA}} - \alpha^{(reg)}[auxin]_{ss} & 0 \\ 0 & -\beta[ARF]_{ss} & -\frac{1}{\tau_{ARF}} - \beta[IAA]_{ss} \end{pmatrix}. \tag{S8}$$

Because the Jacobian is tridiagonal, its eigenvalues are given by the diagonal entries. These are all negative, showing that the steady state is always linearly stable. Furthermore, the steady state is all the more stable that these eigenvalues are large in absolute value. (Note that the associated characteristic times, generally referred to as the relaxation times, are given by minus the inverse of these eigenvalues.) We then see from the second eigenvalue that regulation with $\alpha^{(reg)} > \alpha^{(no-reg)}$ leads to *enhanced* stability.

D. Regulation's role in the linear dynamical response functions

The linearized dynamics determine not only the time-dependent behaviour of *infinitesimal* deviations from the steady state but also their *response* to infinitesimal perturbations of the input. Using the same notation as before, suppose that the auxin source term is allowed to have infinitesimal variations in time:

$$S_{auxin}(t) = S_{auxin} + \Delta S_{auxin}(t). \tag{S9}$$

The system will respond to these variations in the input *via*

$$\frac{d\overrightarrow{\Delta C(t)}}{dt} = \overrightarrow{\Delta S}(t) + \mathbf{J}\overrightarrow{\Delta C(t)}, \tag{S10}$$

where for what follows we shall take $\overrightarrow{\Delta S}(t) = \{\Delta S_{auxin}(t), 0, 0\}$ *i.e.*, we focus on perturbations corresponding to injecting auxin into the system according to an arbitrary time-dependent flux $\Delta S_{auxin}(t)$. Assuming the system is in its steady state before the perturbation is applied, these equations lead to:

$$\overrightarrow{\Delta C}(t) = \int_{-\infty}^{t} \chi(t-t')\overrightarrow{\Delta S}(t')dt', \tag{S11}$$

where the 3×3 matrix

$$\chi(t-t') = \exp[\mathbf{J}(t-t')], \tag{S12}$$

for $t \geq t'$ is called the linear dynamical response function. (By definition, $\chi(t-t')$ is taken to be 0 for $t < t'$.) Taking $t \geq t'$, the (ij)'th entry of this matrix, $\chi_{ij}(t-t')$, can be interpreted as the value of $\overrightarrow{\Delta C}_i(t)$ arising if one applies to the system in its steady state a delta function pulse for the source $\overrightarrow{\Delta S}_j$ at time t'. This dynamical response function (a matrix function of time) is to be distinguished from the *static* response function $\vec{R}$ which is a time-independent vector whose components R_1, R_2, R_3 are simply the derivatives of the steady-state concentrations with respect to the intensity of the steady-state input:

$$\vec{R} = \left\{ \frac{d[auxin]_{ss}}{dS_{auxin}}, \frac{d[IAA]_{ss}}{dS_{auxin}}, \frac{d[ARF]_{ss}}{dS_{auxin}} \right\}, \tag{S13}$$

It is not difficult to show that:

$$R_i = \int_0^{+\infty} \chi_{i1}(t-t')dt'. \tag{S14}$$

In Figures 1:C2-C3 we display the components $\chi_{i1}(t-t')$ ($i = 1, 2, 3$ and $t' = 0$) hereafter denoted $\chi_{auxin}(t)$, $\chi_{IAA}(t)$ and $\chi_{ARF}(t)$, for the models with and without regulation. The case $i = 1$ provides the time-dependent response of auxin (which is not affected by regulation in this model). Examining Eq. 2.1, we see that $\Delta[auxin]$ rises instantaneously by one unit when a perturbation is applied in the form of an instantaneous (delta function at $t' = 0$) pulse of unit integral, and thereafter it simply decays exponentially at rate $-\mathbf{J}_{11} = 1/\tau_{auxin}$. If one integrates over time to have what is called the *total* response to the pulse, we see that the total response is given by the ratio: total

input (here equal to 1) divided by the decay rate of the species (auxin); thus the total response of the auxin molecular species is τ_{auxin}.

Consider now the response displayed by the second molecular species, IAA ($i = 2$). Because the input pulse increases auxin concentration instantaneously, $\Delta[IAA]$ initially decreases linearly with time with a slope $-\alpha[IAA]_{ss}$. After reaching a minimum value, it then relaxes back to 0. If the relaxational dynamics of auxin is fast compared to that of IAA, one can neglect the spontaneous decay of $\Delta[IAA]$ for the short time during which the excess auxin is present. In this approximation, $\Delta[IAA]$ will then reach a minimum given by $-\alpha[IAA]_{ss}$ times the total response of auxin (τ_{auxin}). We thus see that $-\alpha[IAA]_{ss}$ plays the role of an amplification factor. As previously stated, $[IAA]_{ss}$ and similar expressions refer to their steady-state values.

After reaching its minimum, $\Delta[IAA]$ will slowly recover, returning to zero roughly exponentially at the rate given by $\mathbf{J}_{22}$. Interestingly, the total response, defined as for the case of auxin (*cf.* previous paragraph) via integration of the response over all times, can be calculated here directly also. Indeed, the equation $d\Delta[IAA](t)/dt = \mathbf{J}_{21}\Delta[auxin](t) + \mathbf{J}_{22}\Delta[IAA](t)$ can be integrated, leading to:

$$\int_{-\infty}^{+\infty} \Delta[IAA](t)dt = \frac{\mathbf{J}_{21}}{\mathbf{J}_{22}} \int_{-\infty}^{+\infty} \Delta[auxin](t)dt. \tag{S15}$$

As a result, we see that whether there is regulation or not, the integral over time of the dynamical response function is the same. (Recall that regulation rescales $\mathbf{J}_{21}$ and $\mathbf{J}_{22}$ by the same factor $\alpha^{(reg)}/\alpha^{(no-reg)}$, see expressions S7 and S8). Interestingly, the generality of this result follows from the fact that this total response is given by Eq. S14 which itself depends only on the steady-state concentrations (*cf.* Eq. S13). Since by construction the input-output curves are the same with and without regulation, we see that the total response is also the same.

The case of the response displayed by ARF (the third molecular species) can be treated similarly. After an influx pulse of auxin, $\Delta[ARF]$ will initially rise quadratically in time and then it will reach a maximum. If the relaxation time of ARF is large compared to the time during which the IAA signal (the perturbation of IAA concentration) is significant, then this maximum value is approximately $-\beta[ARF]_{ss}$ times the total response of $\Delta[IAA]$, itself given by Eq. S15. As a result, this maximum will be insensitive to regulation. If on the contrary the relaxation time of ARF is comparable to that of IAA or smaller, then the maximum value with regulation will be amplified approximately by a factor $\alpha^{(reg)}/\alpha^{(no-reg)}$, just as it is for IAA. After reaching its maximum, $\Delta[ARF]$ will decay back to 0. And just as for $\Delta[IAA]$, the *total* ARF response is the same whether there is regulation or not.

E. Modifying the minimal model by using true mass action kinetics

In the minimal model considered so far, auxin degrades IAA but this degradation has no consequence on auxin. In the same vein, that model lets IAA degrade ARF but without the IAA dynamics being affected. In reality, the actions arise through the formation of complexes which lead to changing the dynamics of all the molecular actors involved. It is thus natural to ask whether the conclusions reached in that minimal (and feed-forward) model still hold if one uses a more realistic description of the dynamics that correctly includes mass-action in the reactions. That is the purpose of this section. For this extended model to be specified, we have auxin act on IAA by first forming an effective auxin-IAA complex which mimics the auxin-TIR1-IAA complex. This effective auxin-IAA complex then degrades IAA and releases auxin, in direct analogy with what happens biochemically. Similarly, to mimic the role of IAA on ARF, we have them form heterodimers according to mass action kinetics. These heterodimers act in effect to sequester ARF, just as in the non-minimal models. These changes make a bit more complex the minimal model but for the most part one can nevertheless study this modified model analytically.

The equations of the minimal model have to be modified to take into account the formation of the auxin-IAA and ARF-IAA complexes. Furthermore, in the spirit of our new model, we consider that ARF is long lived so that its total concentration (free plus in the ARF-IAA complex) is fixed. The use of mass action for the formation of complexes then changes Eqs. 1 - 3:

$$\frac{d[auxin]}{dt} = S_{auxin} - [auxin]/\tau_{auxin} - \alpha[auxin][IAA] + \gamma[auxin - IAA], \tag{S16}$$

$$\frac{d[IAA]}{dt} = S_{IAA} - [IAA]/\tau_{IAA} - \alpha[auxin][IAA] - \beta[IAA][ARF] + \delta[ARF - IAA], \tag{S17}$$

$$\frac{d[ARF]}{dt} = -\beta[IAA][ARF] + \delta[ARF - IAA], \tag{S18}$$

where we have also taken into account the choice of no production or degradation of ARF. The new parameters γ and δ are the dissociation rates of the auxin-IAA and ARF-IAA complexes. The dynamics of the concentration of auxin-IAA and ARF-IAA complexes adds one additional differential equation:

$$\frac{d[auxin - IAA]}{dt} = \alpha[auxin][IAA] - \gamma[auxin - IAA], \tag{S19}$$

and also one constraint:

$$[ARF - IAA] = ARF_T - [ARF]\,, \tag{S20}$$

where ARF_T is the total concentration of ARF (free or in the ARF-IAA complex). Because of this constraint, we do not need to follow explicitly the dynamics of [ARF-IAA] in this extended model as it is not an independent quantity.

The steady-state concentrations of the four independent molecular species are readily obtained:

$$[auxin]_{ss} = S_{auxin}\tau_{auxin}, \tag{S21}$$

$$[IAA]_{ss} = S_{IAA}/(\alpha\,[auxin]_{ss} + 1/\tau_{IAA}), \tag{S22}$$

$$[ARF]_{ss} = ARF_T/(1 + \beta\,[IAA]_{ss}/\delta), \tag{S23}$$

$$[auxin - IAA]_{ss} = \alpha\,[auxin]_{ss}\,[IAA]_{ss}/\gamma. \tag{S24}$$

Note that each equation involves only terms determined from previous equations so the whole system can be solved by using these equations in the order of appearance.

So far we have implicitly considered no regulation. To include regulation, just as in the feed-forward minimal model, we rescale α and adjust S_{IAA} so that the steady states remain the same. The procedure used in the feed-forward model works exactly in the same way, and in fact the expressions for S_{IAA} in the two minimal models are identical and given in Eq. S4.

Having now defined the sequestrating minimal model with and without regulation, let us consider the steady-state behaviour for increasing S_{auxin}. Supplementary Figures S1:A1-A3 shows that the inclusion of the mass action dynamics (and associated sequestration) does not qualitatively change the dependence of concentrations of auxin, IAA or ARF on S_{auxin} (compare these curves to the ones in Figure 1:B1-B3).

What about the linear dynamical response function? To investigate that, we compute the Jacobian matrix giving the linearized dynamics about the steady state. In contrast to the feed-forward minimal model where the Jacobian was a 3×3 matrix, here there are 4 molecular species to consider as dynamical variables so the Jacobian is a 4×4 matrix. Except for that greater complexity, the framework for computing the response functions is the same. In Supplementary Figures S1:B1-B3 we show these functions giving the time dependence of the variations in concentrations of auxin, IAA and ARF when one introduces an infinitesimal pulse of auxin into the system at $t = 0$.

The conclusion is that again the minimal model with full mass action behaves very much like the feed-forward minimal model, the negative feedback showing an amplification in the dynamical response compared to the unregulated case.

From a biological point of view, note that the release from sequestration allows for a fast response to an auxin signal, bypassing any (long) times associated with transcription or translation. This feature is shared with the Vernoux model and also our new model of Sect. 4 in the Main text.

II. MATHEMATICAL ANALYSIS OF THE MODEL OF VERNOUX *ET AL.*

In the last sub-section, we show how the dynamics of the model provide a decomposition of the network into modules, without the need for any other information.

A. Rate of transcription

The rate equation of transcription for producing the IAA mRNAs (Eq. 3.5 in the main text) follows from the thermodynamical framework of Bintu *et al.* [3]. It involves multiple rates of transcription because Vernoux *et al.* assume that there are two AuxREs (auxin response elements) in the regulatory region of the gene coding for IAA. They allow for a first (basal) level of transcription when that region is free, another one when it is occupied by one ARF, and a last one when it is occupied by two ARFs. The binding of the heterodimer ARF-IAA to either of the AuxREs is assumed to shut off transcription, in line with the idea that this heterodimer acts as an inhibitor of transcription. The final expression used by Vernoux *et al.* for Eq. 3.5 is:

$$h([IAA],[ARF],[ARF-IAA]) = \frac{1+\frac{f}{B_d}[ARF]\left(1+\frac{\omega_A f_A}{B_d}[ARF]\right)}{1+\frac{[ARF]}{B_d}\left(1+\frac{\omega_A}{B_d}[ARF]\right)+\frac{\omega_I}{K_d B_d}[IAA][ARF]+\frac{\omega_D}{B_d}[ARF-IAA]+\kappa_A^-}. \quad \text{(S25)}$$

The term κ_A^- is motivated by the presence of additional species competing for the binding to the regulatory region but not leading to any transcription. These could be for instance “repressor” ARFs which bind to the AuxRE but do not recruit the RNA polymerase (ARF repressors are known to exist). Furthermore, f and f_A quantify the transcriptional amplification due to, respectively, one ARF activator and two ARF activators being bound to the regulatory region. The coefficients ω_A, ω_I and ω_D indicate cooperativity effects stemming from the binding to the DNA of two ARF activators (ω_A) or the formation of dimers (ω_I and ω_D); K_d and B_d are the dissociation constants for the ARF-IAA dimerization and the ARF binding to DNA reaction.

B. Steady States

To solve for the steady state(s), we set to 0 the left hand side of Eqs. (4)-(8) and (10) of the main text. A convenient strategy to solve the resulting equations is to first express all quantities in terms of the concentration of IAA protein. Based on Eqs. (6) and (7), at steady state one has:

$$[IAA_2]_{ss} = \frac{k_{II}([IAA]_{ss})^2}{k'_{II}+\delta^*_{II}+\delta_{II}(x)}, \quad \text{(S26)}$$

and

$$[ARF-IAA]_{ss} = \frac{k_{IA}[IAA]_{ss}[ARF]_{ss}}{k'_{IA}+\delta^*_{IA}+\delta_{IA}(x)}. \quad \text{(S27)}$$

It is useful to rewrite these relations as:

$$k'_{II}[IAA_2]_{ss} - k_{II}([IAA]_{ss})^2 + \delta_{II}(x)[IAA_2]_{ss} = -\alpha_{II}([IAA]_{ss})^2, \quad \text{(S28)}$$

$$k'_{IA}[ARF-IAA]_{ss} - k_{IA}[IAA]_{ss}[ARF]_{ss} + \delta_{IA}(x)[ARF-IAA]_{ss} = -\alpha_{IA}[IAA]_{ss}[ARF]_{ss}, \quad \text{(S29)}$$

with $\alpha_{II} = \frac{k_{II}\delta^*_{II}}{k'_{II}+\delta^*_{II}+\delta_{II}(x)}$ and $\alpha_{IA} = \frac{k_{IA}\delta^*_{IA}}{k'_{IA}+\delta^*_{IA}+\delta_{IA}(x)}$. Then, by plugging Eq. S29 into Eq. 3.2, one obtains at steady state:

$$[ARF]_{ss} = \frac{\pi_A}{\delta_A+\alpha_{IA}[IAA]_{ss}}. \quad \text{(S30)}$$

Thus the steady-state concentrations of ARF, ARF-IAA, and IAA_2 are all known in terms of $[IAA]_{ss}$. As a result, the rate of production of IAA messenger RNA is also known:

$$h([IAA]_{ss},[ARF]_{ss},[ARF-IAA]_{ss}) = h\left([IAA]_{ss}, \frac{\pi_A}{\delta_A+\alpha_{IA}[IAA]_{ss}}, \frac{\pi_A\alpha_{IA}([IAA]_{ss})^2}{\delta^*_{IA}(\delta_A+\alpha_{IA}[IAA]_{ss})}\right). \quad \text{(S31)}$$

Eq. 3.5 then provides the concentration of IAA messenger RNA. As a result, knowing the concentration of IAA protein determines the steady-state concentrations of *all* the other species, as promised.

To obtain now the steady-state concentration of IAA protein, we enforce equality of IAA production rate (given by $\pi_I [IAA_m]_{ss}$ where $[IAA_m]_{ss}$ is known in terms of $[IAA]_{ss}$) and IAA total decay rate. This total decay rate is the sum of the degradation rates of IAA in all of its different forms. Using Eq. 3.1 and the previously derived equations, this total decay rate is:

$$2\alpha_{II}([IAA]_{ss})^2 + \delta_I(x)[IAA]_{ss} + \delta_{II}(x)[IAA_2]_{ss} + \alpha_{IA}[IAA]_{ss}[ARF]_{ss} + \delta_{IA}(x)[ARF - IAA]_{ss}, \quad \text{(S32)}$$

where again all quantities are to be re-expressed in terms of $[IAA]_{ss}$. The associated self-consistent equation can be described graphically by having $[IAA]_{ss}$ on the x-axis and by putting on the y-axis (i) the rate of production of IAA protein and (ii) the total decay rate of IAA protein. Where these two curves cross gives the value of $[IAA]$ in the steady state. The solution is unique in the Vernoux model because the first curve is strictly decreasing (the feedback loop is negative) while the second curve is strictly increasing (the more IAA is present, the more it gets degraded).

C. The dynamics provide an a posteriori decomposition of the network into modules

To define their dynamical equations, the authors of the Vernoux model [2] used the quasi-steady-state approximation to replace the ubiquitination pathway by effective rates for the degradation of IAA (*cf.* in particular Eqs. 4 and **??**). Might it be possible to use this kind of approximation to further reduce their model? The linearized dynamics provides a systematic way to do so since each eigenmode of the Jacobian has a characteristic relaxation time. If we focus on the fastest relaxing mode in the Vernoux model, we find that it mainly involves ARF and ARF-IAA. This means that the kinetic coefficients for the formation and dissociation of ARF-IAA are large so one may use the quasi-equilibrium approximation for that reaction. One could thus replace the Vernoux model by a simpler one where ARF and ARF-IAA are no longer dynamical quantities but are given by Eqs. S30 and S27. To reduce still further the complexity of the model, we can consider the next mode, associated with the second shortest relaxation time. This eigenmode involves mainly IAA and IAA_2. Thus using the same approach one may decide to have IAA_2 be in quasi-equilibrium with IAA and remove it as a dynamical variable. This leads us to ask what is the role of IAA_2 in the Vernoux model in the next sub-section.

D. Role of IAA homodimer

A sensible guess for the role of the IAA homodimer is that it may act as a kind of reservoir to buffer or delay the response to changes in auxin concentrations. To investigate this question, we have taken the Vernoux model and modified it so that there is no longer any formation of IAA_2 (this can be done for instance by simply setting $k_{II} = 0$). Just as when we compared the Vernoux model with and without regulation, it is necessary here to adjust some of the rates for the steady states to be nearly the same with and without IAA homodimer. It is possible to do so as shown in the Supplementary Figure S2. Given that property of the steady-states, we can of course look at the dynamical properties. Supplementary Figure S2 shows that one consequence of *removing* the formation of IAA_2 is to *quicken* the response to an auxin perturbation. A second consequence is to increase the amplitude of the associated dynamical response. Both of these features seem desirable in a signaling network, justifying why in our new model, we have opted for no homodimerization of IAA.

E. Resilience in the Vernoux model

Characteristic resilience times in the Vernoux model were obtained by applying specific perturbations to the system and seeing when the amplitudes of the responses were down by a given factor. We did these measurements in the Vernoux model, both with and without the negative feedback. Illustrative results are given in Supplemental Table S1.

III. QUALITATIVE AND QUANTITATIVE ASPECTS OF OUR NEW CALIBRATED MODEL OF AUXIN SIGNALING

As mentioned in the Main part of the paper, the model is available on the BioModels repository. The first sub-section explains the bioinformatic search for AuxREs motifs in *Arabidopsis* IAA regulatory regions. The second and third sub-section cover the mathematical aspects of our model. The forth provides details on the calibration of the resulting model while the actual numerical values of the 17 parameters are given in Supplemental Table S3.

A. Scanning the *Arabidopsis* IAA regulatory regions for AuxREs and analysis of their clustering

Auxin Response Factors are transcription factors and as such they regulate other genes. Interestingly, they also are involved in IAA transcriptional regulation. In *Arabidopsis*, 23 different ARFs have been identified in the past few decades among which 5 seem to act as activators of IAA transcription (ARF5-8 and 19) while others are believed to act as repressors [4]. As previously discussed, ARFs can directly interact with IAA to form a hetero-dimer; that hetero-dimer probably competes with ARF in binding to AuxRE, and may act as an inhibitor of transcription. The putative static network underlying ARF and IAA interactions is given in ref. [2].

In this system, activators normally initiate transcription by binding to the Auxin Response Elements (AuxRE) upstream of the region coding for IAA. ARFs generally consist of a DNA Binding Domain (DBD) that recognises the consensus sequence TGTCTC along the DNA [5]. This binding motif has been further refined during the past years by both directed mutation experiments [6] and bioinformatic searches [7], leading to the construction of specific Position Weight Matrices (PWMs). ARF23 is the only exception and it is made of a *truncated* DBD [4]. All the other ARFs show furthermore a domain associated with their function as activators or repressors of transcription [8]. In ARF 1-12, 14-15 and 18-22, that domain is connected to two other ones, called III and IV, which allow binding to IAA and some other ARFs [4]; those two domains are the key domains for ARFs to form hetero- and homo-dimers. As we previously discussed, IAA binding ARFs prevents transcriptional activation. Activator ARFs can initiate transcription and are expected to bind DNA as monomers or dimers [6]. Binding by the dimer should be preferred to binding by the monomer if the AuxRE allows it [6]. However, a scan of the upstream regions of the 21 *Arabidopsis* IAA genes, searching for the AuxRE consensus sequences, did not show the existence of any pairs of positions allowing for binding via ARF homo-dimerization, i.e., consensus motifs were not found to be separated by about 10 nucleotides (one helix turn), other than in one case (Table S2). A refinement of such an analysis by using PWMs instead of the consensus sequence overcomes the constraint of requiring the presence of the exact consensus sequence. This can be done, e.g., via available open source programs which, given a PWM, allow one to score sequences and return a p-value for each position detected as significant [9]. In our specific case, we wrote a shell script to scan PWMs against the 21 *Arabidopsis* IAA upstream sequences. For completeness, we compared the obtained scores with those generated by scanning the same PWMs against reshuffled sequences: the program selects then as “true AuxRE” all those positions whose scores were higher than the highest random one. This allows one to get a set of positions labeled as AuxREs and potentially corresponding to true binding sites. In order to probe the possibility of binding by homo-dimers, we determined whether the distance between two subsequent motifs is close to 10 nucleotides (corresponding to a full turn of the DNA). Motivated by what is seen from the crystal structures [6], one can also expect the homodimers to have mirror symmetry in which case the paired AuxREs should be oppositely oriented (Figure 3:B). In our analysis of the sequences, we used two different PWMs, respectively from [6] and [7], from which we identified 16 and 38 hits (Figures 3:C1-C2 and C3-C4 respectively). A study of the distances between hits did not show any signal indicating presence of adjacent binding sites and thus we found no evidence in favour of homodimerization. This is quite in agreement with the results previously found by [10]. Such a divergence between that work and the indications from crystal structures leads to a puzzle in ARFs homo-dimer vs monomer DNA binding that might be sorted out with further experiments in the next few years. For the moment, in our model, we shall take into account both choices, where binding arises via a monomer or a dimer of ARF.

It remains an open question still which is the way ARFs bind to the AuxREs of the regulatory regions of IAA genes. In this regard, we shall consider in our modeling both possibilities, having transcription activation via ARF monomers or dimers.

Along with homo-dimerization, it has been recently proposed that ARFs may be able to form higher order complexes, e.g. in the form of *oligomers* [11]. In this scenario, both activators and repressors might participate together in binding the DNA; it would be interesting to study which are the consequences of oligomerization on the functioning of the system, either focusing on the ARF and IAA species or more generally within a full network including auxin signaling components.

B. The dynamical equations for each molecular species

The dynamical equations of our model are as follows. IAA transcription is described via the reaction:

$$\emptyset \xrightarrow{\lambda_1 F_1} mRNA, \tag{S33}$$

where F_1 is a function of $[ARF]$ and $[ARF-IAA]$ (the two species which can bind to the AuxRE) and λ_1 is the rate of IAA mRNA production when the AuxRE is bound by an ARF (monomer) molecule. F_1 is in fact the probability that ahe AuxRE is bound by an ARF transcription factor; its functional form reflects an underlying thermodynamic equilibrium. Explicitly, we take the choice made in [12] and adapt it to our setting so that:

$$F_1([ARF],[ARF-IAA],[ARF_2]) = \frac{\frac{[ARF]}{\theta_{ARF}}}{1+\frac{[ARF]}{\theta_{ARF}}+\frac{[ARF-IAA]}{\theta_{ARF-IAA}}+\frac{[ARF_2]}{\theta_{ARF2}}}. \tag{S34}$$

Transcription requires that the AuxRE be bound by ARF. Note that ARF-IAA acts as a competitive binder and that when it is bound there is no transcription. These IAA messenger RNAs have a finite lifetime:

$$IAA_m \xrightarrow{\mu_{IAA_m}} \emptyset. \tag{S35}$$

This reaction proceeds at the rate μ_{IAA_m} times the concentration of the IAA messengers. The messengers are also used as templates for translation, leading to the production of IAA proteins. For the purpose of the modeling, the multiple steps involved in translation are simply coarse grained into one bulk reaction, *i.e.*:

$$IAA_m \xrightarrow{\delta} IAA_m + IAA, \tag{S36}$$

with a rate δ times the concentration of the IAA messenger. IAA degradation can arise via two routes: spontaneous, corresponding to a natural lifetime of the protein, or active, catalyzed by auxin-dependent biochemical processes. The spontaneous route is relevant mainly for very low concentrations of auxin and the corresponding rate parameter is μ_{IAA}:

$$IAA \xrightarrow{\mu_{IAA}} \emptyset. \tag{S37}$$

The *active* degradation process depends on the formation of complexes containing auxin and its receptor TIR1. Auxin can bind to TIR1 proteins to form an auxin-TIR1 complex, which in turn may bind IAA. In this complex, IAA is then ubiquitinated, that is some of the protein's residues become tagged, changing IAA into IAA*. The set of these reactions reads as in ref. [12]:

$$\begin{aligned} auxin + TIR1 &\underset{k_d}{\overset{k_a}{\rightleftharpoons}} auxin - TIR1, \\ auxin - TIR1 + IAA &\underset{l_d}{\overset{l_a}{\rightleftharpoons}} auxin - TIR1 - IAA, \\ auxin - TIR1 - IAA &\xrightarrow{l_m} IAA^* + [auxin - TIR1], \\ IAA^* &\xrightarrow{\mu_{IAA^*}} \emptyset. \end{aligned} \tag{S38}$$

In the spirit of keeping the model relatively simple, we use mass action to describe all the kinetics of formation and dissociation of complexes. The other complexes in our model are the ARF-IAA and ARF$_2$ dimers, corresponding to the processes:

$$ARF + IAA \underset{p_d}{\overset{p_a}{\rightleftharpoons}} ARF - IAA, \tag{S39}$$

where p_a and p_d are respectively the association and the dissociation rates of this heterodimer. Similarly, we have:

$$ARF + ARF \underset{q_d}{\overset{q_a}{\rightleftharpoons}} ARF_2, \tag{S40}$$

where q_a and q_d are respectively the association and the dissociation rates of the ARF homodimer.

As mentioned in the previous section, the total concentration of ARF is considered to be fixed to some given amount $ARF_T = [ARF] + 2[ARF_2] + [ARF - IAA]$. The same holds for TIR1 molecules, with $TIR_T = [TIR1] + [auxin - TIR1] + [auxin - TIR1 - IAA]$.

Lastly, we assume that auxin is pumped into the system at a rate S_{auxin}:

$$\emptyset \xrightarrow{S_{auxin}} auxin. \tag{S41}$$

Since auxin is introduced into the system, it must also be either evacuated or degraded. We choose the latter option, introducing an effective lifetime (independent of the cell's state):

$$auxin \xrightarrow{\mu_{auxin}} \emptyset, \tag{S42}$$

where $\mu_{auxin} = 1/\tau_{auxin}$. The union of all these processes allows us now to completely specify the model mathematically based on a set of ordinary differential equations for the time dependence of the concentration of all 10 molecular species:

$$\begin{aligned}
\frac{d[IAA_m]}{dt} &= \lambda_1 F_1 - \mu_{IAA_m}[IAA_m] \\
\frac{d[IAA]}{dt} &= \delta[IAA_m] - \mu_{IAA}[IAA] - l_a[IAA][auxin - TIR1] \\
&\quad + l_d[auxin - TIR1 - IAA] - p_a[ARF][IAA] \\
&\quad + p_d[ARF - IAA] \\
\frac{d[TIR1]}{dt} &= -k_a[auxin][TIR1] + k_d[auxin - TIR1] \\
\frac{d[auxin - TIR1]}{dt} &= k_a[auxin][TIR1] - k_d[auxin - TIR1] \\
&\quad + (l_d + l_m)[auxin - TIR1 - IAA] - l_a[auxin - TIR1][IAA] \\
\frac{d[auxin - TIR1 - IAA]}{dt} &= l_a[IAA][auxin - TIR1] - (l_d + l_m)[auxin - TIR1 - IAA] \\
\frac{d[IAA^*]}{dt} &= l_m[auxin - TIR1 - IAA] - \mu_{IAA^*}[IAA^*] \\
\frac{d[ARF]}{dt} &= -2q_a[ARF]^2 + 2q_d[ARF_2] - p_a[ARF - IAA] + p_d[ARF][IAA] \\
\frac{d[ARF - IAA]}{dt} &= p_a[ARF][IAA] - p_d[ARF - IAA] \\
\frac{d[ARF_2]}{dt} &= q_a[ARF]^2 - q_d[ARF_2] \\
\frac{d[auxin]}{dt} &= S_{auxin} + k_d[auxin - TIR1] - k_a[auxin][TIR1] - \mu_{auxin}[auxin]
\end{aligned}, \tag{S43}$$

Our goal is to study how the concentration of IAA and of the driving factor ARF are affected by changes in auxin influx. Having specified the model in a formal way, it still has to be calibrated, i.e., its 17 parameters must be set, 15 being associated with rates, the two remaining being respectively the total amount of ARF and the total amount of TIR1.

C. Determining the steady states

We follow the strategy previously used to compute the steady states in the simpler models: we first express all concentrations in terms of that of IAA and then we derive the self-consistent equation for $[IAA]_{ss}$. Although the expressions of all the different quantities in terms of $[IAA]_{ss}$ are complicated, at the end one obtains one self-consistent equation for $[IAA]_{ss}$ which is easily solved numerically. To illustrate the successive steps, begin by imposing the steady-state condition on the complexes involving ARF. This leads to:

$$\begin{aligned}
[ARF - IAA]_{ss} &= P[ARF]_{ss}[IAA]_{ss} \\
[ARF_2]_{ss} &= Q[ARF]_{ss}^2
\end{aligned}, \tag{S44}$$

where $P = p_a/p_d$ and $Q = q_a/q_d$. By using the conservation law for total amount of ARF protein, one gets:

$$ARF_T = P[ARF]_{ss}[IAA]_{ss} + [ARF]_{ss} + 2Q([ARF]_{ss})^2, \quad \text{(S45)}$$

which has a unique positive solution for $[ARF]_{ss}$ as a function of $[IAA]_{ss}$. Furthermore, since $[ARF]_{ss}$ and $[ARF - IAA]_{ss}$ are known in terms of $[IAA]_{ss}$, the same holds for $[IAA_m]_{ss}$.

Consider now the ubiquitination module. The steady-state conditions on the concentrations $[auxin - TIR1]_{ss}$ and $[auxin - TIR1 - IAA]_{ss}$ lead to:

$$\begin{aligned} [auxin - TIR1]_{ss} &= K\frac{S_{auxin}}{\mu_{aux}}[TIR1]_{ss} \\ [auxin - TIR1 - IAA]_{ss} &= L[IAA]_{ss}[auxin - TIR1]_{ss} = \frac{LKS_{auxin}}{\mu_{aux}}[IAA]_{ss}[TIR1]_{ss} \end{aligned}, \quad \text{(S46)}$$

where we use the notation of ref. [12]: $K = k_a/k_d$ and $L = l_a/(l_d + l_m)$. Note that we also used the steady-state expression for auxin concentration, $[auxin]_{ss} = S_{auxin}/\mu_{aux}$. As TIR1 total concentration is fixed too, one can use the same logic as before, leading to:

$$TIR1_T = [TIR1]_{ss} + \frac{KS_{auxin}}{\mu_{aux}}[TIR1]_{ss} + \frac{LKS_{auxin}}{\mu_{aux}}[IAA]_{ss}[TIR1]_{ss}, \quad \text{(S47)}$$

whose solution for TIR1 can be expressed in terms of the parameters and $[IAA]_{ss}$. We will denote this relation by $[TIR1]_{ss} = g([IAA]_{ss})$.

Now that the concentrations of all species have been determined in terms of that of IAA, the self-consistent equation for $[IAA]_{ss}$ is obtained by imposing that the rate of production of IAA protein (translation) is equal to its (passive and active) decay rate. This gives immediately:

$$\frac{\lambda_1 \delta}{\mu_{IAA_m}} F_1([IAA]_{ss}) = \mu_{IAA}[IAA]_{ss} + (l_a - l_d L)\frac{KS_{auxin}}{\mu_{aux}}[IAA]_{ss} g([IAA]_{ss}). \quad \text{(S48)}$$

The graphical representation of this self-consistent equation is provided in Figure 4:A. Just as for the Vernoux model, by monotonicity of the two curves there exist a unique solution for the steady-state concentration of IAA. We can compute it numerically for any given set of parameters and auxin influx rate S_{auxin}. Then, by plugging the solution obtained for $[IAA]_{ss}$ into the other equations, one obtains all steady-state concentrations in the network (Figures 4:B1-B3). The behaviors of auxin, IAA and ARF concentrations as a function of S_{auxin} are qualitatively the same as in the models analyzed in the previous sections.

D. Model calibration and use of diffusion limited reactions

The values of all 17 parameters of our new calibrated model are provided in Table S3. They are also given in the model definition in the BioModels repository. The setting of these values rested on estimates from published works such as [13, 14], constraints coming from measurements of equilibrium constants, and theoretical bounds for “on” rates. This last type of constraint is not commonly used so we now explain in detail how it arises and can be used.

The rate k_{on} in any reaction cannot be arbitrarily large because it is necessarily bounded from above by the so called “diffusion limit”. To understand where this limit comes from, consider a reaction A + B which produces C. Motivated by the case where B is an enzyme, for pedagogical purposes consider that B is at a given point and that the A molecules diffuse throughout the cell volume. A necessary condition for forming a C molecule is the encounter or “collision” of an A molecule with B. The number of collisions per unit time felt by B, k_S, can be computed [15] and it is referred to as the *Smoluchowski encounter rate*:

$$k_S = 4\pi D_A R_t \rho_A, \quad \text{(S49)}$$

where D_A is the diffusion constant of the A molecules, R_t is the radius of the target zone “offered” by B for the reaction and ρ_A is the density of A molecules in the cell volume. Note that k_S does not have the same dimensions as k_{on}: indeed k_S gives the number of collision per unit of time while k_{on} has dimensions $1/(M \cdot s)$. To relate k_S to k_{on}, one must first use the definition of k_{on}:

$$\frac{d[C]}{dt} = k_{on}[A][B] - k_{off}[C], \quad \text{(S50)}$$

and then convert from numbers of molecules per unit volume to concentrations in moles per liter, giving $[A] = n_A/N_A V_{cell}$, where n_A is the number of molecules of A in the cell, N_A is Avogadro's number and V_{cell} is the cell volume expressed in litres.

Analogously, since we took one molecule of B in the cell, one obtains $[B] = 1/N_A V_{cell}$. Plugging these expressions into the differential equation for $[C]$ and using k_S one finally gets:

$$k_{on} = 1000 N_A 4\pi D_A R_t. \tag{S51}$$

The Einstein-Smoluchowski relation and Stokes' equation for mobilities of spheres in viscous liquids allow one to give an estimate of the diffusion constant, $D_A = k_B T/6\pi\eta R_A$, where η is the viscosity of the liquid and R_A is the radius of the molecule. Inserting this relation in the previous one for k_{on} one gets:

$$k_{on} = 1000 N_A \frac{2}{3} \frac{k_B T}{\eta} \frac{R_t}{R_A}. \tag{S52}$$

Recall that R_t is the radius of the contact region offered by B for the reaction. For most reactions it is believed to be typically $R_t \simeq 1 - 2$ nm. For our derivation, we assumed B did not diffuse. If instead both A and B molecules diffuse, the relation is modified by considering as the diffusion constant the sum of both diffusion constants, D_A and D_B. This leads to a final expression for k_{on} given by:

$$k_{on} = 1000 N_A \frac{4}{3} \frac{k_B T R_t}{\eta} \Big[\frac{1}{R_A} + \frac{1}{R_B} \Big]. \tag{S53}$$

For our purposes, we considered $T = 298.15K$ (as in [16]), $\eta = 1.5 \cdot 10^{-2} P$ for the viscosity of the cytoplasm [17], and the other known constants also being given by the literature. To determine the characteristic sizes R_A and R_B when A and B are ARF and IAA, we first studied their domains using published crystal structures, in particular the DBD and the III/IV domains for ARFs and the III/IV domains for IAA. We visualized the structures of these domains using UCSF Chimera [18] to obtain the characteristic size of these molecules. Since each protein is multi-domain, the estimates of R_{ARF} and R_{IAA} require some care. For ARFs, the crystal structures do not furnish any information about the junction domain between the DBD and the III/IV. We thus assumed this domain to have the same dimensions as the smallest one, *i.e.*, III/IV. Our approach was to consider the multi-domain molecules as rods formed by aligning the domains, and to use the result for diffusion of rigid rods obtained computationally in ref. [19]. This approach leads to an effective radius of the protein using Stokes' law, leading to $R_{ARF} = 8.75 \quad nm$. In the case of IAA, we assumed the four domains to be all similar to the III/IV ones. The reasoning then led to an equivalent radius for IAA equal to $R_{IAA} \simeq 3.2$nm. Plugging these values and $R_t = 2$nm into the equation for k_{on} and using the measurements of the dissociation constants, K_D, in ref. [16] allowed us to determine k_{off}. The corresponding estimates of q_a, q_d, p_a and p_d are given in Tab. S3. Setting the other parameters in our new model described in Sect. 4 was possible using estimates from the literature (see in particular ref. [13]) so for instance the value of λ_1 was typical of genes constitutively expressed. Furthermore we used theoretical reasonings, for instance based on the bounds from the diffusion limit. With all this information it was possible to obtain values for all 17 parameters of this new model.

E. Complexity reduction via time-scale separation

In this subsection, we ask whether a decomposition of our new model into modules can be obtained *automatically* without any *a priori* biases. To do so we use the separation of time scales approach along with Principal Component Analysis [20]. Principal Component Analysis is generally used on sets of points in high dimensional spaces to obtain a lower dimensional projection. In effect, it first builds the multi-Gaussian distribution having the same mean and covariance matrix as the considered set of points and then it projects the high-dimensional set of points onto the main principal axes, that is the directions corresponding to the leading eigenvectors of that covariance matrix so as to maintain the maximum amount of variance. In the method of time-scale separation of a dynamical system, one can analyze the Jacobian matrix $\mathbf{J}$ describing the linearized dynamics about a steady state. When treating the dynamics of the deviations from the steady-state concentrations, the most general form of these linearized dynamics can be written as:

$$\frac{d\overrightarrow{\Delta C(t)}}{dt} = \mathbf{J}\overrightarrow{\Delta C(t)}, \tag{S54}$$

where $\overrightarrow{\Delta C(t)}$ is the variation with respect to the steady-state of the different concentrations in the system. Whenever the Jacobian matrix is invertible, the previous equation can be integrated in terms of its eigenvalues and eigenvectors,

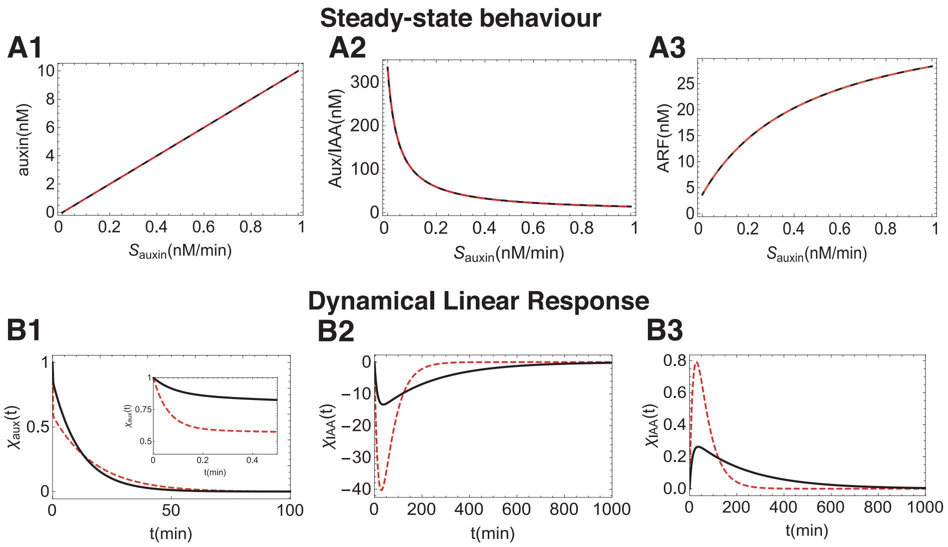

FIG. S1: **Mass-action extended minimal model.** A1-A3) The steady-state input-output relations for auxin, IAA and ARF in the mass-action extended minimal model as a function of S_{auxin} (the incoming flux of auxin), with and without the regulatory feedback (dashed red lines and black thick lines respectively). By construction, the regulated and unregulated cases have the same input-output relation in the steady state. Parameter values: τ_{auxin}=10 min, τ_{IAA}= 333 min, $\alpha^{(no-reg)}$=0.007 (nM min)$^{-1}$, $\alpha^{(reg)}$=0.03 (nM min)$^{-1}$, β=0.3 (nM min) $^{-1}$, γ=10 min^{-1}, δ=10 min^{-1} and ARF$_T$=40 nM. B1-B3) The linear response functions for auxin, IAA and ARF in the unregulated and regulated cases (black thick lines and dashed red lines and respectively). Parameter values taken as in panel A other than S_{auxin}=0.02 nM min^{-1}.

namely λ_k and $|w_k\rangle$, where k goes from 1 to the total number of independent molecular species. In this space of eigenvectors the Jacobian is diagonal and so each eigenvector simply decays with time as $\exp(\lambda_k t)$.

In general λ_k can be complex, but for the decay rate of that eigenvector what matters is the real part of λ_k (by stability of our system, this value is negative). One then defines the relaxation time of that eigenvector as the inverse of the opposite of that real part. The higher the rate of relaxation, the shorter the corresponding relaxation time. In the separation of time-scales approach, one wants to identify the fast parts of the dynamics. Formally, the quasi-steady state approximation can be obtained by taking to infinity the eigenvalues of the fast eigenvectors. If one does that, the amplitude of those fast eigenmodes are in effect set to 0, corresponding to enforcing a (quasi-equilibrium) constraint between the involved molecular species. To interpret the meaning of those fast modes, one can use Principal Component Analysis. For illustration, consider the two fastest modes of our model which was defined in Sect. 4. We represent in Supplementary Figure S3, for each species, its components on those two eigenvectors based on the steady state when $S_{auxin} = 0.02$ nM min^{-1}. The separation of time scales is seen to be quite simple: (1) the fastest mode (x-axis of the figure) consists mainly of auxin-TIR1, IAA and auxin-TIR1-IAA, meaning again that the association and dissociation rates of the triple complex are high so one has quasi-equilibrium between those species; (2) the second fastest mode (y-axis of the figure) consists mainly of ARF, IAA and ARF-IAA, meaning that the association and dissociation rates are high so one has quasi-equilibrium between those species. Were one to take the on and off rates of these two reactions to infinity, then formally the species ARF-IAA and auxin-TIR1-IAA could be removed from the dynamics and their dynamical equation replaced by a constraint. (Note that in practice such a change is not particularly helpful from a numerical point of view.)

In Supplementary Figure S3 the “slow” species occur near the origin, e.g., IAA$_m$ and ARF$_2$. The first is easy to justify: the relaxation time of IAA_m is intrinsically long because the lifetime of the messenger RNAs are on the order of an hour, which is much longer than the time scale of any enzymatic biochemical reaction. The slowness of ARF$_2$ on the other hand was not necessarily expected. Indeed, since the association and dissociation rates of the heterodimer ARF-IAA are high, one might have anticipated them to be high also for the ARF homodimer. We expect that the separation of scales between these dimers reflects the role of ARF-IAA in the sequestration while the function of ARF$_2$ is associated with ARF's downstream targets (see last sub-sections).

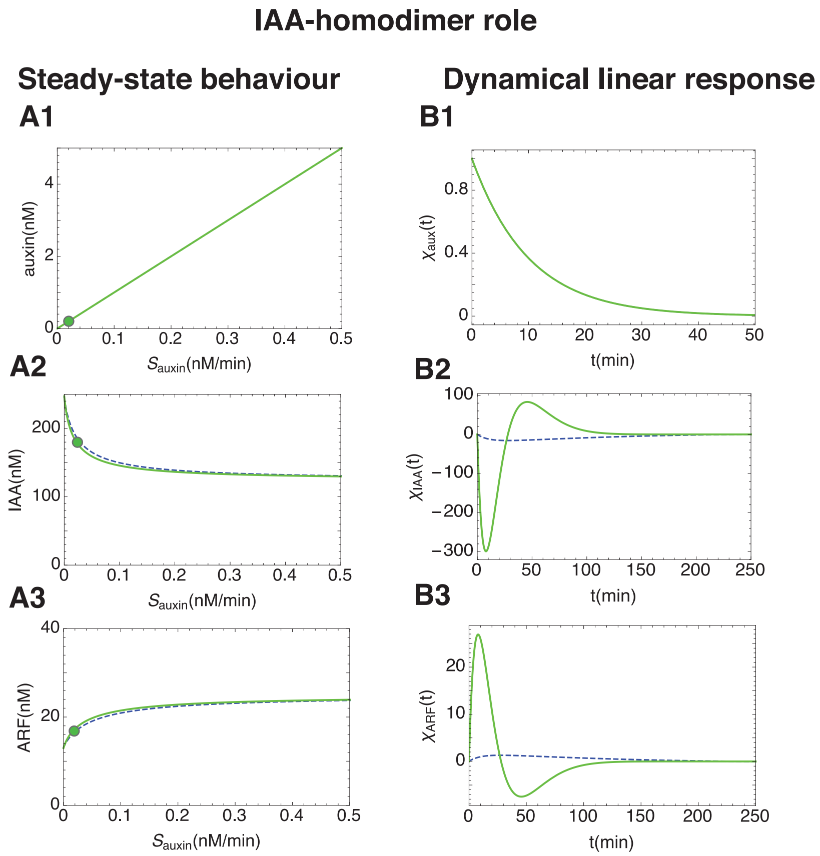

FIG. S2: **Influence of IAA$_2$, the IAA homodimer, in the Vernoux model.** A1-A3: the adjustment of decay rates allows for nearly identical steady-states when comparing the original model (blue dashed curves) and the model without any homodimer (green thick curves). More precisely we have multiplied both δ_I and K by a factor 1.3. B1-B3: Allowing for the formation of IAA$_2$ both slows the response and decreases its amplitude.

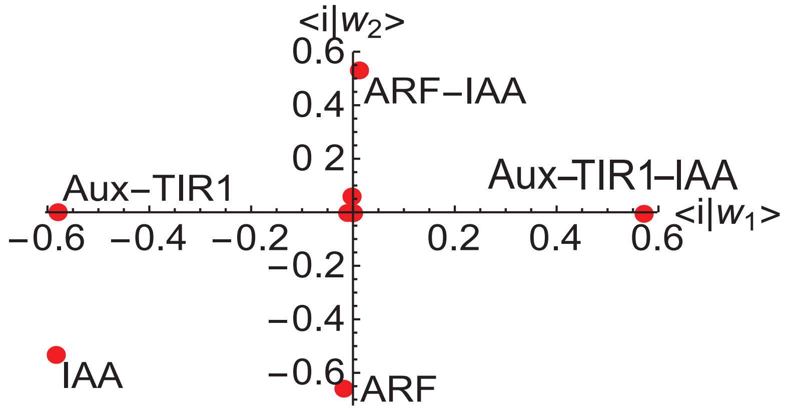

FIG. S3: **Result of Principal Component Analysis applied on the Jacobian matrix of our new model.** For each molecular species, we represent its coordinate in the space of the two eigenvectors of the Jacobian matrix that have eigenvalues with the most negative real part. The coordinates for species i are scalar products, respectively $\langle i|w_1\rangle$ and $\langle i|w_2\rangle$. The points away from the origin of the plane and thus contributing to the eigenvectors have been labeled according to the associated molecular species.

S_{auxin} (nM/min)	$\delta TIAA$ Regulated (min)	$\delta TIAA$ Unregulated (min)	δT ARF Regulated (min)	δT ARF Unregulated (min)
0.02	155.7	435.3	153.3	431.6
0.2	68.1	316.2	67.6	315.5
S_{auxin} (nM/min)	$\delta TIAA$ Regulated (min)	$\delta TIAA$ Unregulated (min)	δT ARF Regulated (min)	δT ARF Unregulated (min)
0.02	167.8	437.0	168.0	437.0
0.2	72.1	315.9	72.2	315.8

TABLE S1: **Resilience in the Vernoux model.** Given are the characteristic resilience times δT defined as the times for responses to decrease from their maximum value to 10% of that value. Both for IAA and for ARF, these times are significantly shorter with regulation (the IAA negative feedback) than without regulation. This is illustrated here for the non-linear regime (top) and the linear (bottom) regimes, but the results hardly differ, regulation (presence of the negative feedback loop on IAA transcription) leading to systematically shorter time scales. For each, the results are given for S_{auxin}=0.02 nM min^{-1} and for S_{auxin}=0.2 nM min^{-1}. The shorter δT, the greater the system's resilience to perturbations. Here the perturbations correspond to instantaneously adding to the system 10 times its steady-state concentration of auxin.

IAA	LOCUS	BS ARF1	D(1-2)	D(2-3)	D(3-4)	D(4-5)	CLUSTERS
17	AT1G04250	0	0	0	0	0	0
5	AT1G15580	1	0	0	0	0	0
18	AT1G51950	1	0	0	0	0	0
8	AT2G22670	5	127	159	258	453	0
13	AT2G33310	1	0	0	0	0	0
20	AT2G46990	3	566	1324	0	0	0
19	AT3G15540	4	1702	33	11	0	1
2	AT3G23030	1	0	0	0	0	0
1	AT4G14560	2	661	0	0	0	0
28	AT5G25890	2	587	0	0	0	0
14	AT4G14550	1	0	0	0	0	0
9	AT5G65670	1	0	0	0	0	0
12	AT1G04550	0	0	0	0	0	0
31	AT3G17600	3	15	134	0	0	0
16	AT3G04730	0	0	0	0	0	0
30	AT3G62100	2	528	0	0	0	0
11	AT4G28640	1	0	0	0	0	0
29	AT4G32280	5	41	100	1319	268	0
4	AT5G43700	2	2	0	0	0	0
33	AT5G57420	0	0	0	0	0	0
15	AT1G80390	1	0	0	0	0	0

TABLE S2: **Results of the consensus sequence scan performed along the 21 IAA-AuxREs chosen for ARF1 binding.** The consensus sequence used is TGTCTC. We defined as *clusters* the groups of motifs whose distance is compatible with one turn of the DNA helix.

REFERENCES

[1] E. Zažímalová, J. Petrášek, and E. Benková, *Auxin and its role in plant development*. Springer, 2014.

[2] T. Vernoux, G. Brunoud, E. Farcot, V. Morin, H. Van den Daele, J. Legrand, M. Oliva, P. Das, A. Larrieu, D. Wells, Y. Guédon, L. Armitage, F. Picard, S. Guyomarc'h, C. Cellier, G. Parry, R. Koumproglou, J. H. Doonan, M. Estelle, C. Godin, S. Kepinski, M. Bennett, L. De Veylder, and J. Traas, "The auxin signalling network translates dynamic input into robust patterning at the shoot apex," *Molecular Systems Biology*, vol. 7, no. 1, 2011.

[3] L. Bintu, N. E. Buchler, H. G. Garcia, U. Gerland, T. Hwa, J. Kondev, and R. Phillips, "Transcriptional regulation by the numbers: models," *Current Opinion in Genetics & Development*, vol. 15, no. 2, pp. 116 – 124, 2005. Chromosomes and expression mechanisms.

[4] T. J. Guilfoyle and G. Hagen, "Auxin response factors," *Current Opinion in Plant Biology*, vol. 10, no. 5, pp. 453 – 460, 2007. Cell Signalling and Gene RegulationEdited by Jian-Kang Zhu and Ko Shimamoto.

[5] M. Del Bianco and S. Kepinski, "Context, specificity, and self-organization in auxin response," *Cold Spring Harbor perspec-*

Parameter	Value
**New Calibrated Model**	
μ_{IAA_m}	0.003 min^{-1}
μ_{IAA}	0.003 min^{-1}
δ	4 min^{-1}
μ_{aux}	0.1 min^{-1}
μ_{IAA^*}	0.1 min^{-1}
λ_1	0.48 nM min^{-1}
k_a	8.2 10^{-4} (nM min)$^{-1}$
k_d	0.33 min^{-1}
p_a	1 (nM min)$^{-1}$
p_d	0.072 min^{-1}
q_a	0.5 (nM min)$^{-1}$
q_d	0.44 min^{-1}
$l_m^{non-reg}$	0.009 min^{-1}
l_m^{reg}	0.9 min^{-1}
l_d	0.045 min^{-1}
$l_a^{non-reg}$	0.575 (nM min)$^{-1}$
l_a^{reg}	5.75 (nM min)$^{-1}$
θ_{ARF}	100 nM
$\theta_{ARF-IAA}$	100 nM
θ_{ARF2}	100 nM
ARF_T	200 nM
$TIR1_T$	100 nM
**Toggle Switch**	
t_{ARF}	2 min^{-1}
K_{ARF}	4.5 nM
α_1	0.5 nM min^{-1}
α_2	0.5 nM min^{-1}
δ_1	0.1 min^{-1}
δ_2	0.1 min^{-1}
β	4
γ	4

TABLE S3: **List of parameter values in our new calibrated model FLAIR and in the toggle switch.** These have been chosen in particular based on estimates provided in refs. [2, 13] and the insights obtained from our dynamical analyses in this paper. For the toggle switch, we used both results from FLAIR and the analysis reported in ref. [21].

tives in biology, vol. 3, no. 1, p. a001578, 2011.

[6] D. R. Boer, A. Freire-Rios, W. A. van den Berg, T. Saaki, I. W. Manfield, S. Kepinski, I. López-Vidrieo, J. M. Franco-Zorrilla, S. C. de Vries, R. Solano, *et al.*, “Structural basis for dna binding specificity by the auxin-dependent arf transcription factors,” *Cell*, vol. 156, no. 3, pp. 577–589, 2014.

[7] J. Keilwagen, J. Grau, I. A. Paponov, S. Posch, M. Strickert, and I. Grosse, “De-novo discovery of differentially abundant transcription factor binding sites including their positional preference,” *PLoS Comput Biol*, vol. 7, pp. 1–13, 02 2011.

[8] S. B. Tiwari, G. Hagen, and T. Guilfoyle, “The roles of auxin response factor domains in auxin-responsive transcription,” *The Plant Cell*, vol. 15, no. 2, pp. 533–543, 2003.

[9] J. Korhonen, P. Martinmki, C. Pizzi, P. Rastas, and E. Ukkonen, “Moods: fast search for position weight matrix matches in dna sequences,” *Bioinformatics*, vol. 25, no. 23, p. 3181, 2009.

[10] V. V. Mironova, N. A. Omelyanchuk, D. S. Wiebe, and V. G. Levitsky, “Computational analysis of auxin responsive elements in the arabidopsis thaliana l. genome,” *BMC genomics*, vol. 15, no. 12, p. S4, 2014.

[11] M. H. Nanao, T. Vinos-Poyo, G. Brunoud, E. Thévenon, M. Mazzoleni, D. Mast, S. Lainé, S. Wang, G. Hagen, H. Li, *et al.*, “Structural basis for oligomerization of auxin transcriptional regulators,” *Nature communications*, vol. 5, 2014.

[12] A. M. Middleton, J. R. King, M. J. Bennett, and M. R. Owen, “Mathematical modelling of the aux/iaa negative feedback loop,” *Bulletin of Mathematical Biology*, vol. 72, no. 6, pp. 1383–1407, 2010.

[13] L. R. Band, D. M. Wells, A. Larrieu, J. Sun, A. M. Middleton, A. P. French, G. Brunoud, E. M. Sato, M. H. Wilson, B. Péret, M. Oliva, R. Swarup, I. Sairanen, G. Parry, K. Ljung, T. Beeckman, J. M. Garibaldi, M. Estelle, M. R. Owen, K. Vissenberg, T. C. Hodgman, T. P. Pridmore, J. R. King, T. Vernoux, and M. J. Bennett, “Root gravitropism is regulated by a transient lateral auxin gradient controlled by a tipping-point mechanism,” *Proceedings of the National Academy of Sciences*, vol. 109, no. 12, pp. 4668–4673, 2012.

[14] K. A. Dreher, J. Brown, R. E. Saw, and J. Callis, “The arabidopsis aux/iaa protein family has diversified in degradation and

auxin responsiveness," *The Plant Cell*, vol. 18, no. 3, pp. 699–714, 2006.
[15] N. Dorsaz, C. De Michele, F. Piazza, P. De Los Rios, and G. Foffi, "Diffusion-limited reactions in crowded environments," *Physical review letters*, vol. 105, no. 12, p. 120601, 2010.
[16] M. Han, Y. Park, I. Kim, E.-H. Kim, T.-K. Yu, S. Rhee, and J.-Y. Suh, "Structural basis for the auxin-induced transcriptional regulation by aux/iaa17," *Proceedings of the National Academy of Sciences*, vol. 111, no. 52, pp. 18613–18618, 2014.
[17] R. Milo, P. Jorgensen, U. Moran, G. Weber, and M. Springer, "Bionumbers—the database of key numbers in molecular and cell biology," *Nucleic Acids Research*, vol. 38, no. suppl 1, pp. D750–D753, 2010.
[18] E. F. Pettersen, T. D. Goddard, C. C. Huang, G. S. Couch, D. M. Greenblatt, E. C. Meng, and T. E. Ferrin, "Ucsf chimera: a visualization system for exploratory research and analysis," *Journal of computational chemistry*, vol. 25, no. 13, pp. 1605–1612, 2004.
[19] A. Ortega and J. Garcıa de la Torre, "Hydrodynamic properties of rodlike and disklike particles in dilute solution," *The Journal of chemical physics*, vol. 119, no. 18, pp. 9914–9919, 2003.
[20] V. Plerou, P. Gopikrishnan, B. Rosenow, L. A. N. Amaral, and H. E. Stanley, "Universal and nonuniversal properties of cross correlations in financial time series," *Physical Review Letters*, vol. 83, no. 7, p. 1471, 1999.
[21] T. S. Gardner, C. R. Cantor, and J. J. Collins, "Construction of a genetic toggle switch in escherichia coli," *Nature*, vol. 403, no. 6767, pp. 339–342, 2000.

Toy Models

```
ClearAll["Global`*"]
SetOptions[ListLinePlot, BaseStyle → FontSize → 20];
SetOptions[Plot, BaseStyle → FontSize → 20];
```

Toy model without mass-action law

Here we define the toy model without mass-action law: we define the variables’ list, their derivatives and the steady-state variables. We fix the parameters and define the ODEs for the regulated and unregulated case.

```
vt = {aux[t], iaa[t], arf[t]};
vdt = {aux'[t], iaa'[t], arf'[t]};
vss = {auxss, iaass, arfss};
param =
  {τaux → 10, τiaa → 333.33, τarf → 2., Sarf → 25, β → 0.003, α0 → 0.05, α1 → 150};
Siaa = 1. /. param;
Siaareg = (1 / τiaa iaa[t] (1 - α1 / α0) + α1 / α0);
M = {{-1 / τaux, 0, 0}, {0, -1 / τiaa, 0}, {0, 0, -1 / τarf}};
b = {{Saux}, {Siaa - α0 aux[t] * iaa[t]}, {Sarf - β iaa[t] * arf[t]}};
breg = {{Saux}, {Siaareg - α1 aux[t] * iaa[t]}, {Sarf - β iaa[t] * arf[t]}};
rhs = M.vt + b /. param;
rhsss = M.vt + b /. {aux[t] → auxss, iaa[t] → iaass, arf[t] → arfss} /. param;
rhsreg = M.vt + breg /. param;
rhsssreg = M.vt + breg /. {aux[t] → auxss, iaa[t] → iaass, arf[t] → arfss} /. param;

eqn = Table[vdt[[n]] == rhs[[n]][[1]], {n, 1, 3}];
eqnreg = Table[vdt[[n]] == rhsreg[[n]][[1]], {n, 1, 3}];
```

We solve the equations for the steady state as a function of auxin influx.

```
ss = vss /. Flatten@Solve[Table[rhsss[[n]][[1]] == 0., {n, 1, 3}], vss] //
    FullSimplify // Quiet;

ssreg = vss /. Flatten@Solve[Table[rhsssreg[[n]][[1]] == 0., {n, 1, 3}], vss] //
    FullSimplify // Quiet;
```

Steady-state plots

```
ListLinePlot[{Table[{n, ss[[1]] /. {Saux → n}}, {n, 0, 0.5, 0.1}],
   Table[{n, ssreg[[1]] /. {Saux → n}}, {n, 0, 0.5, 0.1}]},
  Frame → True, FrameLabel → {"Sauxin (nM/min)", "auxin (nM)"},
  PlotRange → {{0., 0.5}, All}, PlotStyle → {{Black, Thick}, {Red, Dashed}}]
```

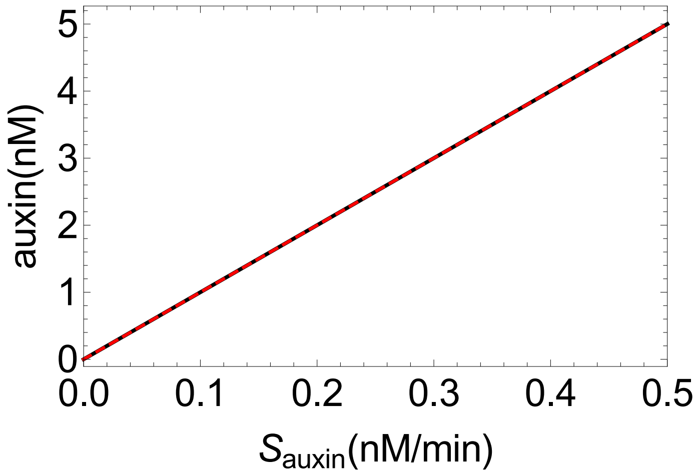


```
auxiaassplot = ListLinePlot[{Table[{n, ss[[2]] /. {Saux → n}}, {n, 0., 0.5, 0.001}],
    Table[{n, ssreg[[2]] /. {Saux → n}}, {n, 0., 0.5, 0.001}]},
   Frame → True, FrameLabel → {"Sauxin (nM/min)", "Aux/IAA (nM)"},
   PlotRange → {All, All}, PlotStyle → {{Black, Thick}, {Red, Dashed}}]
```

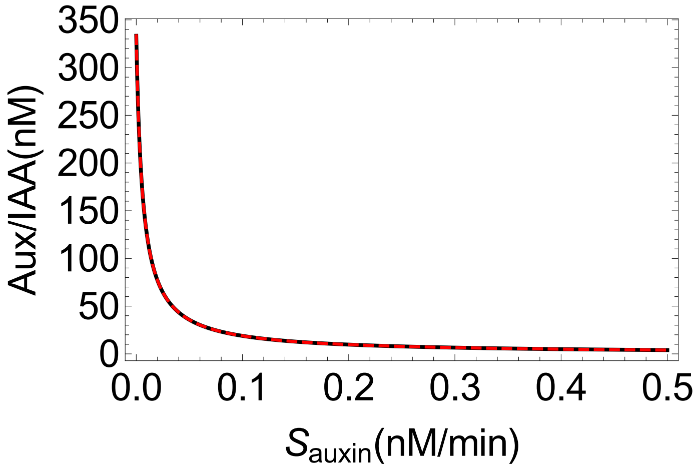

```
arfssplot = ListLinePlot[{Table[{n, (ss[[3]]) /. {Saux → n}}, {n, 0., 0.5, 0.01}],
    Table[{n, (ssreg[[3]]) /. {Saux → n}}, {n, 0., 0.5, 0.01}]},
  Frame → True, FrameLabel → {"Sauxin (nM/min)", "ARF (nM)"},
  PlotRange → {{0., 0.5}, All}, PlotStyle → {{Black, Thick}, {Red, Dashed}}]
```

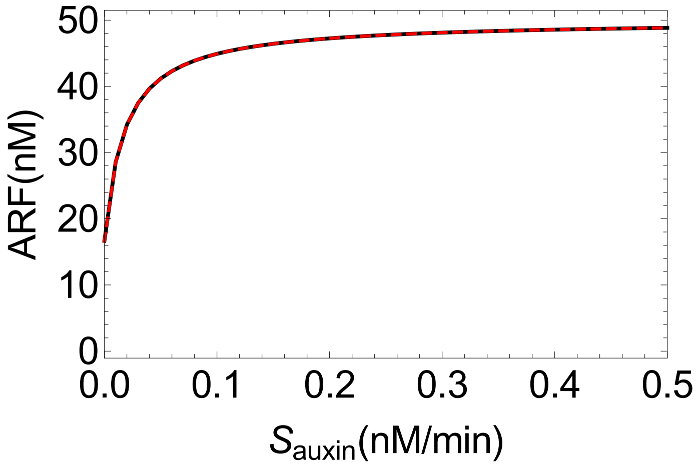

Linear Response Analysis

We hereby implement the Linear Response analysis. We first define the jacobian matrices for the unregulated and regulated case respectively and we then plot the components of the Jacobian matrix's exponential for ARF, IAA and auxin describing their relaxation with respect to auxin perturbations.

```
jacobian = Table[Flatten[Table[D[rhs[[n]], vt[[m]]], {m, 1, 3}], 1], {n, 1, 3}] /.
     {aux[t] → ss[[1]], iaa[t] → ss[[2]], arf[t] → ss[[3]]} /. param;

jacobianreg = Table[Flatten[Table[D[rhsreg[[n]], vt[[m]]], {m, 1, 3}], 1], {n, 1, 3}] /.
    {aux[t] → ssreg[[1]], iaa[t] → ssreg[[2]], arf[t] → ssreg[[3]]};

chiarf = MatrixExp[jacobian * t][[3, 1]] /.
    {auxss → ss[[1]], iaass → ss[[2]], arfss → ss[[3]]};
chiarfreg = MatrixExp[jacobianreg * t][[3, 1]] /.
    {auxss → ssreg[[1]], iaass → ssreg[[2]], arfss → ssreg[[3]]};
chiiaa = MatrixExp[jacobian * t][[2, 1]] /.
    {auxss → ss[[1]], iaass → ss[[2]], arfss → ss[[3]]};
chiiaareg = MatrixExp[jacobianreg * t][[2, 1]] /.
    {auxss → ssreg[[1]], iaass → ssreg[[2]], arfss → ssreg[[3]]};
chiaux = MatrixExp[jacobian * t][[1, 1]] /.
    {auxss → ss[[1]], iaass → ss[[2]], arfss → ss[[3]]};
chiauxreg = MatrixExp[jacobianreg * t][[1, 1]] /.
    {auxss → ssreg[[1]], iaass → ssreg[[2]], arfss → ssreg[[3]]};
```

Linear-response analysis plots

```
Plot[{chiarfreg /. {Saux → 0.02}, chiarf /. {Saux → 0.02}},
 {t, 0, 100}, PlotRange → All, PlotStyle → {{Red, Dashed}, {Black, Thick}},
 Frame → True, FrameLabel → {"t(min)", "χARF(t)"},
 FrameTicks → {{Automatic, Automatic}, {{0, 50, 100}, Automatic}}, AxesOrigin → {0, 0}]
```

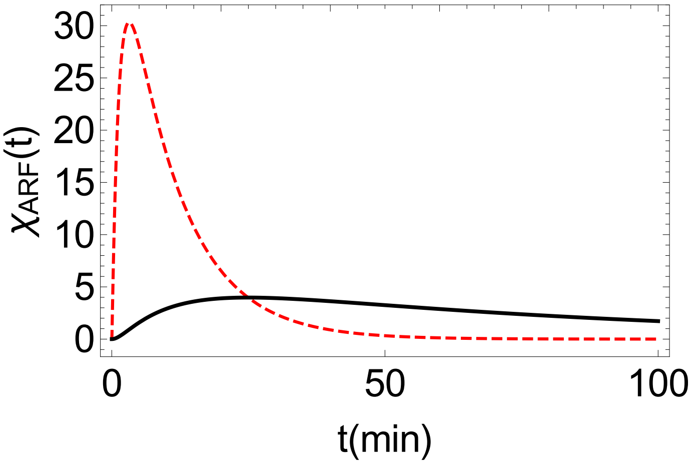


```
Plot[{chiiaareg /. {Saux → 0.02}, chiiaa /. {Saux → 0.02}}, {t, 0., 100.},
 PlotRange → {{0., 100.}, All}, PlotStyle → {{Red, Dashed}, {Black, Thick}},
 Frame → True, FrameLabel → {"t(min)", "χIAA(t)"},
 FrameTicks → {{Automatic, Automatic}, {{0, 50, 100}, Automatic}}, AxesOrigin → {0, 0}]
```

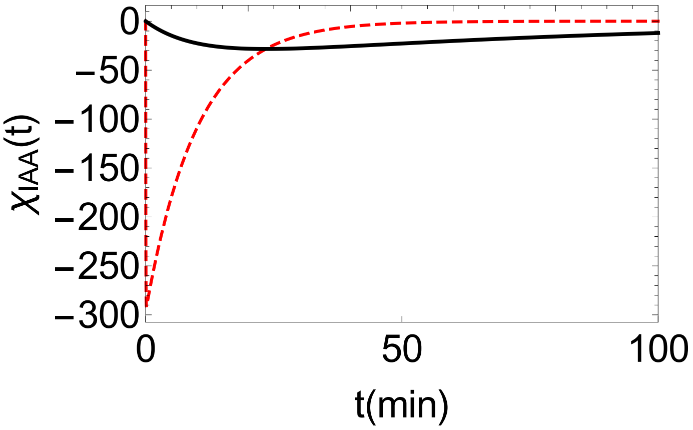

```
Plot[{chiiaareg /. {Saux → 0.02}, chiiaa /. {Saux → 0.02}}, {t, 0., 1.},
 PlotRange → {{0., 1.}, All}, PlotStyle → {{Red, Dashed}, {Black, Thick}},
 Frame → True, FrameLabel → {"t(min)", "χIAA(t)"},
 FrameTicks → {{Automatic, Automatic}, {{0, 0.5, 1}, Automatic}}, AxesOrigin → {0, 0}]
```

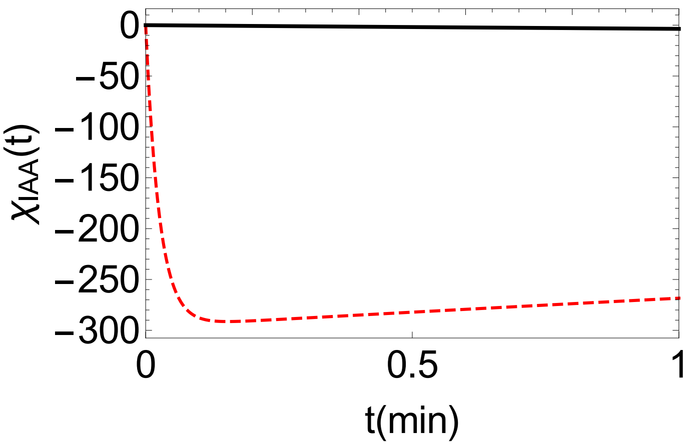


```
Plot[{chiaux /. {Saux → 0.02}, chiauxreg /. {Saux → 0.02}}, {t, 0., 100.},
 PlotRange → {{0., 100.}, All}, PlotStyle → {{Black, Thick}, {Red, Dashed}},
 Frame → True, FrameLabel → {"t(min)", "χaux(t)"}, FrameTicks →
  {{{0, 0.5, 1}, Automatic}, {{0, 50, 100}, Automatic}}, AxesOrigin → {0, 0}]
```

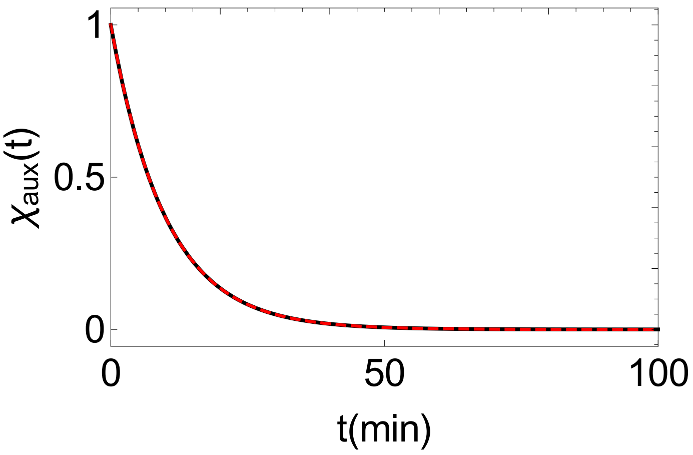

Toy Model with Mass-Action Law

Here we define the toy model with mass-action law: we define the variables' list, their derivatives and the steady-state variables. We fix the parameters and define the ODEs for the regulated and unregulated case.

```
ClearAll["Global`*"]
SetOptions[ListLinePlot, BaseStyle → FontSize → 20];
SetOptions[Plot, BaseStyle → FontSize → 20];
vt = {aux[t], iaa[t], arf[t], arfiaa[t], auxiaa[t]};
vdt = {aux'[t], iaa'[t], arf'[t], arfiaa'[t], auxiaa'[t]};
vss = {auxss, iaass, arfss, arfiaass, auxiaass};

param =
  {τaux → 10, τiaa → 333.33, δ → 10, γ → 10, β → 0.3, α0 → 0.007, α1 → 0.03, ARFT → 40.};

Siaa = 1. /. param;
Siaareg = (1/τiaa iaa[t] (1 - α1 / α0) + α1 / α0);
M = {{-1 / τaux, 0, 0, 0, 0}, {0, -1/τiaa, 0, 0, 0},
    {0, 0, 0, 0, 0}, {0, 0, 0, -δ, 0}, {0, 0, 0, 0, -γ}};
b = {{Saux - α0 aux[t] iaa[t] + γ auxiaa[t]},
    {Siaa - α0 aux[t] * iaa[t] - β arf[t] iaa[t] + δ arfiaa[t]},
    {-β iaa[t] * arf[t] + δ arfiaa[t]}, {β arf[t] iaa[t]}, {α0 aux[t] iaa[t]}};
breg = {{Saux - α1 aux[t] iaa[t] + γ auxiaa[t]},
    {Siaareg - α1 aux[t] * iaa[t] - β arf[t] iaa[t] + δ arfiaa[t]},
    {-β iaa[t] * arf[t] + δ arfiaa[t]}, {β arf[t] iaa[t]}, {α1 aux[t] iaa[t]}};
rhs = M.vt + b /. param;
rhsss =
  Append[M.vt + b, ARFT - arf[t] - arfiaa[t] == 0.] /. {aux[t] → auxss, iaa[t] → iaass,
      arf[t] → arfss, auxiaa[t] → auxiaass, arfiaa[t] → arfiaass} /. param;
rhsreg = Append[M.vt + breg, ARFT - arf[t] - arfiaa[t] == 0.] /. param;
rhsssreg =
  Append[M.vt + breg, ARFT - arf[t] - arfiaa[t] == 0.] /. {aux[t] → auxss, iaa[t] → iaass,
      arf[t] → arfss, auxiaa[t] → auxiaass, arfiaa[t] → arfiaass} /. param;

eqn = Append[Table[vdt[[n]] == rhs[[n]][[1]], {n, 1, 5}], ARFT - arf[t] - arfiaa[t] == 0.];
eqnreg =
  Append[Table[vdt[[n]] == rhsreg[[n]][[1]], {n, 1, 5}], ARFT - arf[t] - arfiaa[t] == 0.];
```

We solve the equations for the steady state as a function of auxin influx.

```
ss = vss /. Flatten@Solve[Table[rhsss[[n]][[1]] == 0., {n, 1, 6}], vss] //
     FullSimplify // Quiet;

ssreg = vss /. Flatten@Solve[Table[rhsssreg[[n]][[1]] == 0., {n, 1, 6}], vss] //
     FullSimplify // Quiet;
```

Steady-state plots

```
ListLinePlot[{Table[{n, ss[[1]] /. {Saux → n}}, {n, 0., 1, 0.001}],
   Table[{n, ssreg[[1]] /. {Saux → n}}, {n, 0., 1, 0.001}]},
  Frame → True, FrameLabel → {"Sauxin (nM/min)", "auxin (nM)"},
  PlotRange → {All, All}, PlotStyle → {{Black, Thick}, {Red, Dashed}}]
```

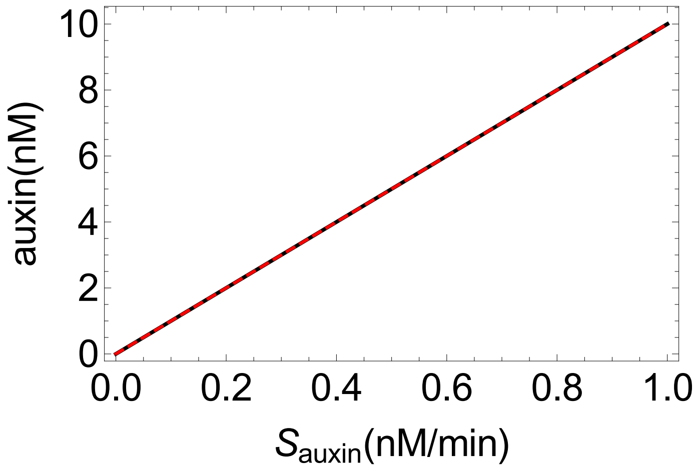


```
auxiaassplot = ListLinePlot[{Table[{n, ss[[2]] /. {Saux → n}}, {n, 0., 1, 0.001}],
    Table[{n, ssreg[[2]] /. {Saux → n}}, {n, 0., 1, 0.001}]},
   Frame → True, FrameLabel → {"Sauxin (nM/min)", "Aux/IAA (nM)"},
   PlotRange → {All, All}, PlotStyle → {{Black, Thick}, {Red, Dashed}}]
```

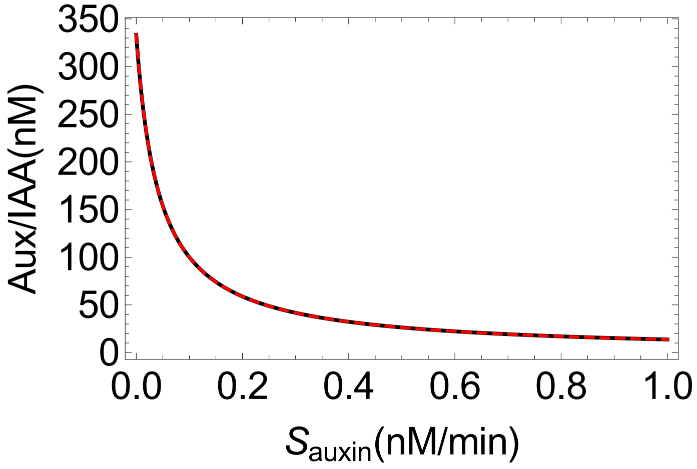

```
arfssplot = ListLinePlot[{Table[{n, (ss[[3]]) /. {Saux → n}}, {n, 0., 1, 0.001}],
    Table[{n, (ssreg[[3]]) /. {Saux → n}}, {n, 0., 1, 0.001}]},
  Frame → True, FrameLabel → {"Sauxin (nM/min)", "ARF (nM)"},
  PlotRange → All, PlotStyle → {{Black, Thick}, {Red, Dashed}}]
```

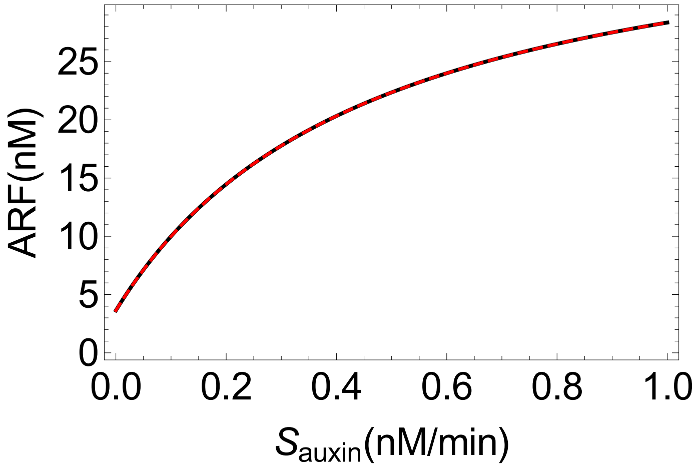

Linear Response Analysis

We hereby implement the Linear Response analysis. We first define the jacobian matrices for the unregulated and regulated case respectively and we then plot the components of the Jacobian matrix's exponential for ARF, IAA and auxin describing their relaxation with respect to auxin perturbations.

```
jacobian = Table[Flatten[Table[D[rhs[[n]], vt[[m]]], {m, 1, 5}], 1], {n, 1, 5}] /.
    {aux[t] → ss[[1]], iaa[t] → ss[[2]], arf[t] → ss[[3]],
     arfiaa[t] → ss[[4]], auxiaa[t] → ss[[5]]};

jacobianreg = Table[Flatten[Table[D[rhsreg[[n]], vt[[m]]], {m, 1, 5}], 1], {n, 1, 5}] /.
    {aux[t] → ssreg[[1]], iaa[t] → ssreg[[2]], arf[t] → ssreg[[3]],
     arfiaa[t] → ssreg[[4]], auxiaa[t] → ssreg[[5]]};

chiarf = MatrixExp[jacobian * t][[3, 1]] /. {auxss → ss[[1]],
     iaass → ss[[2]], arfss → ss[[3]], arfiaa[t] → ss[[4]], auxiaa[t] → ss[[5]]};
chiarfreg = MatrixExp[jacobianreg * t][[3, 1]] /.
    {auxss → ssreg[[1]], iaass → ssreg[[2]], arfss → ssreg[[3]],
     arfiaa[t] → ssreg[[4]], auxiaa[t] → ssreg[[5]]};
chiiaa = MatrixExp[jacobian * t][[2, 1]] /. {auxss → ss[[1]], iaass → ss[[2]],
     arfss → ss[[3]], arfiaass → ss[[4]], auxiaass → ss[[5]]};
chiiaareg = MatrixExp[jacobianreg * t][[2, 1]] /.
    {auxss → ssreg[[1]], iaass → ssreg[[2]], arfss → ssreg[[3]],
     arfiaass → ssreg[[4]], auxiaass → ssreg[[5]]};
chiauxreg = MatrixExp[jacobianreg * t][[1, 1]] /.
    {auxss → ssreg[[1]], iaass → ssreg[[2]], arfss → ssreg[[3]],
     arfiaa[t] → ssreg[[4]], auxiaa[t] → ssreg[[5]]};
chiaux = MatrixExp[jacobian * t][[1, 1]] /. {auxss → ss[[1]], iaass → ss[[2]],
     arfss → ss[[3]], arfiaa[t] → ss[[4]], auxiaa[t] → ss[[5]]};
```

Linear-response analysis plots

```
Plot[{chiarfreg /. {Saux → 0.02} /. param, chiarf /. {Saux → 0.02} /. param},
 {t, 0., 1000.}, PlotRange → {{0., 1000.}, All},
 PlotStyle → {{Red, Dashed}, {Black, Thick}}, Frame → True,
 FrameLabel → {"t(min)", "χIAA(t)"}, AxesOrigin → {0, 0}]
```

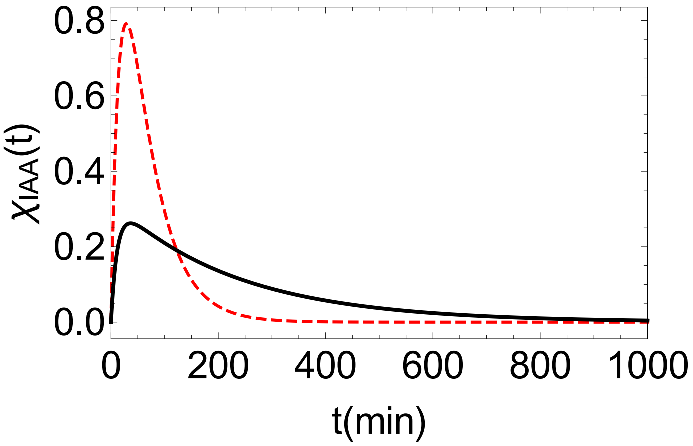


```
Plot[{chiiaareg /. {Saux → 0.02} /. param, chiiaa /. {Saux → 0.02} /. param},
 {t, 0., 1000.}, PlotRange → {{0., 1000.}, All},
 PlotStyle → {{Red, Dashed}, {Black, Thick}}, Frame → True,
 FrameLabel → {"t(min)", "χIAA(t)"}, AxesOrigin → {0, 0}]
```

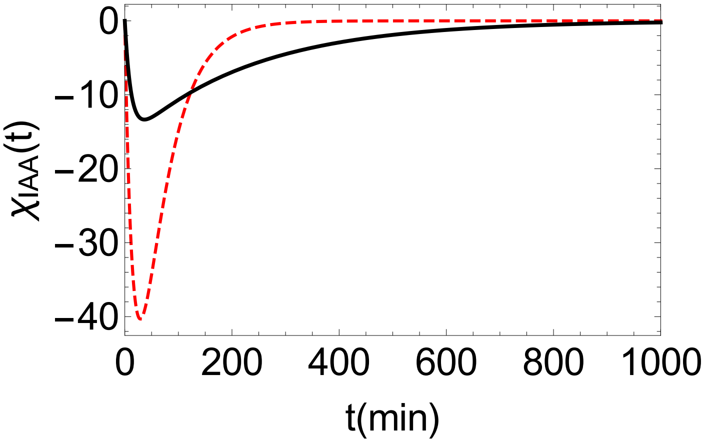

```
Plot[{Chop@chiauxreg /. {Saux → 0.02}, Chop@chiaux /. {Saux → 0.02} /. param},
 {t, 0., 50.}, PlotRange → {{0., 50.}, All},
 PlotStyle → {{Red, Dashed}, {Black, Thick}}, Frame → True,
 FrameLabel → {"t(min)", "χaux(t)"}, FrameTicks →
  {{{0, 0.5, 1}, Automatic}, {{0, 50, 100}, Automatic}}, AxesOrigin → {0, 0}]
```

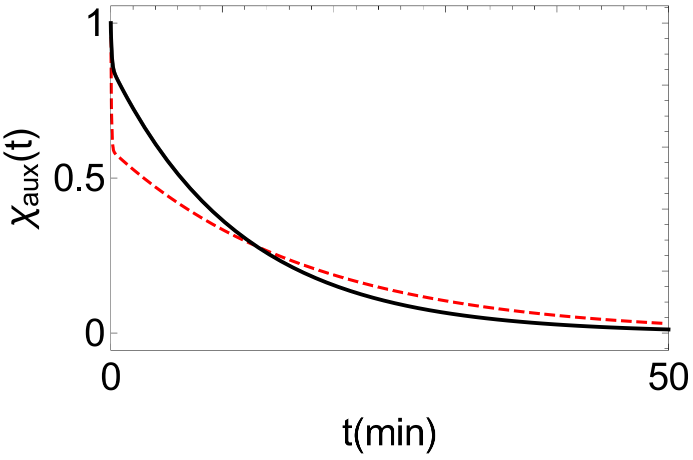

We add here a zoom on auxin behaviour to better visualise the first half minute of relaxation.

```
chiauxinzoom =
 Plot[{chiauxreg /. {Saux → 0.02}, chiaux /. {Saux → 0.02} /. param}, {t, 0., 0.5},
  PlotRange → {{0., 0.5}, {0.4, 1}}, PlotStyle → {{Red, Dashed}, {Black, Thick}},
  Frame → True, FrameLabel → {"t(min)", "χaux(t)"}, FrameTicks →
   {{{0, 0.5, 0.75, 1}, Automatic}, {{0, 0.2, 0.4}, Automatic}}, AxesOrigin → {0, 0}]
```

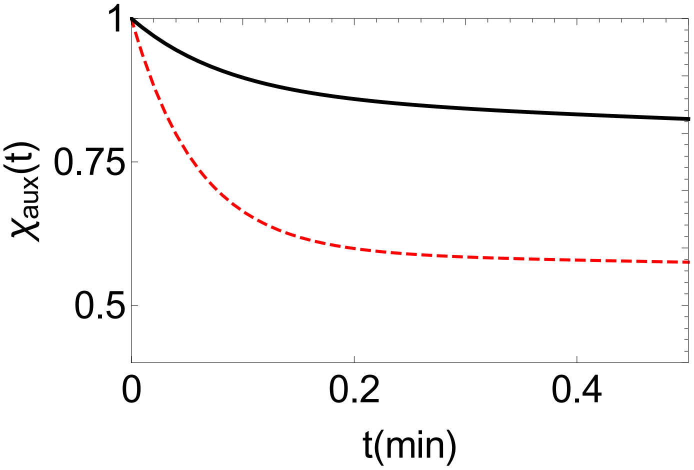

Vernoux Model

```
Remove["Global`*"]
SetOptions[Plot, BaseStyle → FontSize → 18];
SetOptions[ListLinePlot, BaseStyle → FontSize → 18];
SetOptions[ListPlot, BaseStyle → FontSize → 18];
```

System setup

Parameters

```
paramreg = {piI → 1., dr → 0.007, dA → 0.003, dII → 0.003, dIA → 0.003, kII → 1., kIA → 1.,
    bd → 100.0, fA → 10., gammaI → 10., Kaux → 1. , KII → 10, KIA → 10, Ka → 1,
    fC → 10, f → 10, wA → 10, wI → 10, wD → 10., dI → 0.05, piA → 1., kAm → 10.,
    kprimeIA → 10., kprimeII → 10., τ → 10, dstarIA → 0.003, dstarII → 0.003};
param = {piI → 1., dr → 0.007, dA → 0.003, dII → 0.003, dIA → 0.003, kII → 1.,
    kIA → 1., bd → 100.0, fA → 10., gammaI → 2.1, Kaux → 1. , KII → 10, KIA → 10,
    Ka → 5.5, fC → 10, f → 10, wA → 10, wI → 10, wD → 10., dI → 0.05, piA → 1.,
    kAm → 10., kprimeIA → 10., kprimeII → 10., dstarIA → 0.003, dstarII → 0.003,
    τ → 10};
wbasal = 0.00005;
w0 = 0.02;
```

Variables’ definition for time variation, steady state and derivatives

```
vt = {IAA[t], IAA2[t], ARF[t], ARFIAA[t], R[t], aux[t]};
vss = {IAA, IAA2, ARF, ARFIAA, R, aux[t]};
vdt = {{IAA'[t]}, {IAA2'[t]}, {ARF'[t]}, {ARFIAA'[t]}, {R'[t]}, {aux'[t]}};
nspecies = Length[vss];
```

mRNA regulation

```
h = (1 + f / bd * ARF[t] * (1 + fA / bd * wA * ARF[t])) / (1 + ARF[t] / bd * (1 + wA / bd * ARF[t]) +
      wI / (kprimeIA / kIA * bd) * ARF[t] * IAA[t] + wD / bd * ARFIAA[t] + kAm);
dIx = gammaI * dI * aux[t] * Ka / (1 + Ka * aux[t]);
dIAx = gammaI * dIA * aux[t] * Ka / (1 + Ka * aux[t]);
dIIx = gammaI * dII * aux[t] * Ka / (1 + Ka * aux[t]);
f0 = 2 * dstarII * 5865 /. paramreg;
```

Steady states expressions for every species

```
auxss = w * τ;
IAA2ss = kII * IAAss^2 / (kprimeII + dstarII + dIIx) /. {aux[t] → auxss};
ARFIAAss = kIA * IAAss * ARFss / (kprimeIA + dstarIA + dIAx) /. {aux[t] → auxss};
Rss =
  (h /. {IAA[t] → IAAss, ARF[t] → ARFss, ARFIAA[t] → ARFIAAss, aux[t] → auxss}) / dr;
alphaIA = kIA * dstarIA / (kprimeIA + dstarIA + dIAx) /. {aux[t] → auxss};
alphaII = kII * dstarII / (kprimeII + dstarII + dIIx) /. {aux[t] → auxss};
ARFss = piA / (dA + alphaIA * IAAss) /. {aux[t] → auxss};
```

Vector of steady states

```
ss = {IAAss, IAA2ss, ARFss, ARFIAAss, Rss, auxss};
ssconst = {IAAss, IAA2ss, ARFss, ARFIAAss, Rssconst, auxss};
replacess = Table[vt[[n]] → ss[[n]], {n, 1, nspecies}];
```

Definition of the unregulated case: we take the rate of transcription at the basal level of auxin

```
initbasal =
  Block[{w = wbasal}, NSolve[{((piI * Rss - 2 * alphaII * IAAss^2 - alphaIA * IAAss * ARFss -
            dIAx * ARFIAAss - dIIx * IAA2ss - dIx * IAAss) /.
          {aux[t] → auxss} /. paramreg) == 0, IAAss > 0.}, IAAss]];
hnr = Block[{w = wbasal}, h /. replacess /. paramreg /. initbasal[[1]]];
Rssconst = hnr / dr;
```

Dynamical equations

Dynamical equations: regulation vs non-regulation

Definition of linear and nonlinear part of the dynamics, both in the regulated and unregulated case

```
M = {{-dIx, 2 * kprimeII + dIIx, 0, kprimeIA, piI, 0},
    {0, - (kprimeII + dIIx + dstarII), 0, 0, 0, 0},
    {0, 0, -dA, kprimeIA + dIAx, 0, 0}, {0, 0, 0, - (kprimeIA + dIAx + dstarIA), 0, 0},
    {0, 0, 0, 0, -dr, 0}, {0, 0, 0, 0, 0, -1 / τ}};
bneg = {{-2 * kII * IAA[t] ^ 2 - kIA * IAA[t] * ARF[t]}, {kII * IAA[t] ^ 2},
    {piA - kIA * IAA[t] * ARF[t]}, {kIA * IAA[t] * ARF[t]}, {h}, {w}};
bnr = {{-2 * kII * IAA[t] ^ 2 - kIA * IAA[t] * ARF[t]}, {kII * IAA[t] ^ 2},
    {piA - kIA * IAA[t] * ARF[t]}, {kIA * IAA[t] * ARF[t]}, {hnr}, {w}};
```

We hereby define the ‘operative’ values, i.e. including the numerical value of parameters

```
Mopn = M /. paramreg;
Mopc = M /. param;
bopneg = bneg /. paramreg;
bopconst = bnr /. param;
RHSneg = Mopn.vt + bopneg;
RHSconst = Mopc.vt + bopconst;
```

Solution with a perturbation in auxin at t = 0

Initialization at $S_{auxin} = 0.02$ by solving the self-consistent equation for the steady state IAA

```
initneg = Block[{w = w0},
   NSolve[{((piI * Rss - 2 * alphaII * IAAss ^ 2 -
          alphaIA * IAAss * ARFss - dIAx * ARFIAAss - dIIx * IAA2ss - dIx * IAAss) /.
        {aux[t] → auxss} /. paramreg) == 0, IAAss > 0.}, IAAss]];
initconst = Block[{w = w0}, NSolve[{((piI * Rssconst - 2 * alphaII * IAAss ^ 2 -
          alphaIA * IAAss * ARFss - dIAx * ARFIAAss - dIIx * IAA2ss - dIx * IAAss) /.
        {aux[t] → auxss} /. param) == 0, IAAss > 0.}, IAAss]];
```

Initial conditions for regulated and unregulated case: we take as initial conditions the steady states, we include a perturbation in auxin at the inital time t=0 (auxin concentration is increased additively by ten times its initial value)

```
tinit = 0.;
vecpert = {0, 0, 0, 0, 0, 10 * w * τ};
icsneg = Table[(vt[[i]] /. {t → tinit}) == Block[{w = w0},
      (ss[[i]] + vecpert[[i]]) /. initneg[[1]] /. paramreg], {i, nspecies}];
icsconst = Table[(vt[[i]] /. {t → tinit}) == Block[{w = w0},
      (ssconst[[i]] + vecpert[[i]]) /. initconst[[1]] /. param], {i, nspecies}];
```

We hereby compute the solution of the perturbed dynamics

```
eqspertneg = Table[vdt[[i]] == RHSneg[[i]], {i, nspecies}];
eqspertconst = Table[vdt[[i]] == RHSconst[[i]], {i, nspecies}];
eqssysneg = Join[eqspertneg, icsneg];
eqssysconst = Join[eqspertconst, icsconst];
Block[{ tinit = 0., tfin = 10000., w = w0},
 nsol = NDSolve[eqssysneg, vt, {t, tinit, tfin}];
 csol = NDSolve[eqssysconst, vt, {t, tinit, tfin}];];
```

```
dt = 0.1;
c = {0.01, 1 / 700, 0.05, 0.01, 0.05, 1};
```

```
solpertneg =
  Table[Join[Table[{t, Block[{w = w0}, c[[i]] * ss[[i]] /. paramreg /. initneg[[1]]]},
     {t, -50, 0, dt}], Table[{t, Evaluate[c[[i]] * vt[[i]] /. nsol[[1]]]},
     {t, 0.1, 1000, dt}]], {i, nspecies}];
solpertconst = Table[Join[Table[{t, Block[{w = w0},
        c[[i]] * ssconst[[i]] /. param /. initconst[[1]]]}, {t, -50, 0, dt}],
    Table[{t, Evaluate[c[[i]] * vt[[i]] /. csol[[1]]]}, {t, 0.1, 1000, dt}]], {i,
    nspecies}];
```

Plots of time courses with regulation

```
ListPlot[solpertneg, PlotRange → {{-30, 80}, All},
 Joined → True, PlotLegends → {c[[1]] "IAA", c[[2]] "IAA2 ",
   c[[3]] "ARF", c[[4]] "ARFIAA", c[[5]] "mRNA", c[[6]] "aux"}, Frame → True,
 FrameLabel → {"t(min)", "concentration (nM)"}, PlotStyle → Thick]
```

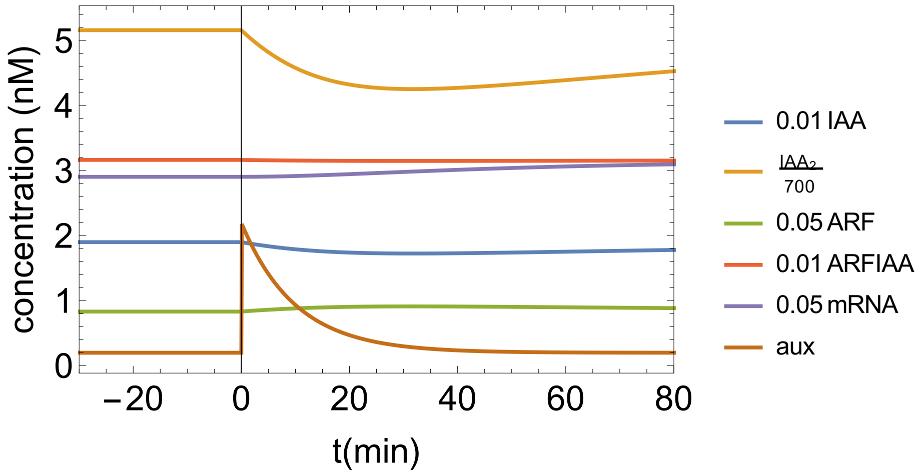

```
ListPlot[solpertconst, PlotRange → {{-30, 80}, All},
 Joined → True, PlotLegends → {c[[1]] "IAA", c[[2]] "IAA2 ",
   c[[3]] "ARF", c[[4]] "ARFIAA", c[[5]] "mRNA", c[[6]] "aux"}, Frame → True,
 FrameLabel → {"t(min)", "concentration (nM)"}, PlotStyle → Thick]
```

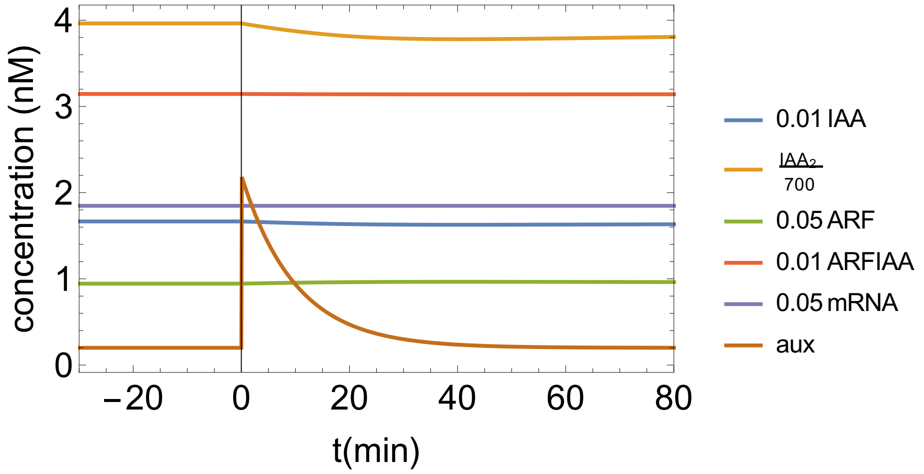


```
ListPlot[{solpertneg[[1]], solpertneg[[3]], solpertneg[[6]]},
 PlotRange → {{-30, 80}, All}, Joined → True,
 PlotLegends → {c[[1]] "IAA", c[[3]] "ARF", c[[6]] "aux"}, Frame → True,
 FrameLabel → {"t(min)", "concentration (nM)"}, PlotStyle → Thick]
```

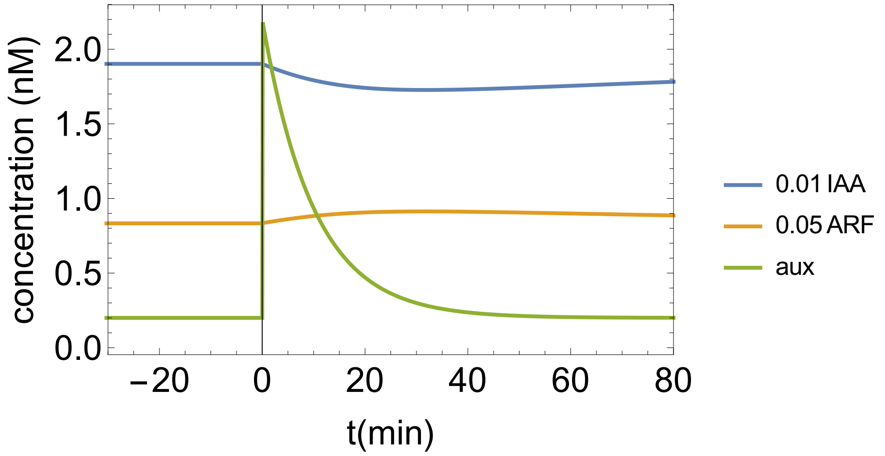

Plots of time courses without regulation

```
ListPlot[{solpertconst[[1]], solpertconst[[3]], solpertconst[[6]]},
 PlotRange → {{-30, 80}, All}, Joined → True,
 PlotLegends → {c[[1]] "IAA", c[[3]] "ARF", c[[6]] "aux"}, Frame → True,
 FrameLabel → {"t(min)", "concentration (nM)"}, PlotStyle → Thick]
```

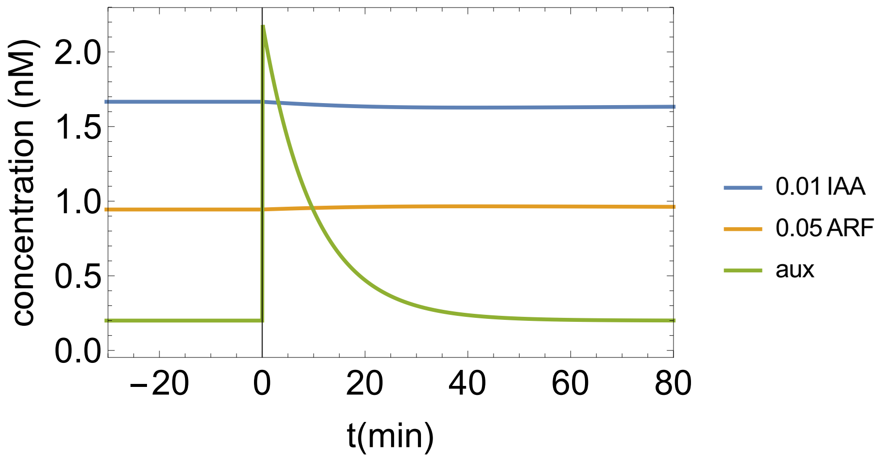

Nonlinear Response χ^{nl}

Hereby we quantify the nonlinear response as the change w.r.t. the steady-state value (assumed for t < 0) caused by the perturbation in auxin.

We define the nonlinear response for the regulated and unregulated case at S_{aux}=0.02

```
chinlreg = Block[{w = w0},
   ((Evaluate[vt /. nsol[[1]]] - ss /. initneg[[1]]) / (10 * w0 * τ)) /. paramreg];
chinl = Block[{w = w0},
   ((Evaluate[vt /. csol[[1]]] - ss /. initconst[[1]]) / (10 * w0 * τ)) /. param];
```

Plots of comparison of nonlinear responses for regulated and unregulated case

```
Plot[{chinlreg[[6]], chinl[[6]]}, {t, 0, 100},
 PlotLegends → {"Regulated", "Unregulated"}, PlotRange → {{0, 50}, All}, Frame → True,
 FrameLabel → {"t(min)", "χ^nl_aux(t)"}, PlotStyle → {{Blue, Dashed}, {Red, Thick}}]
```

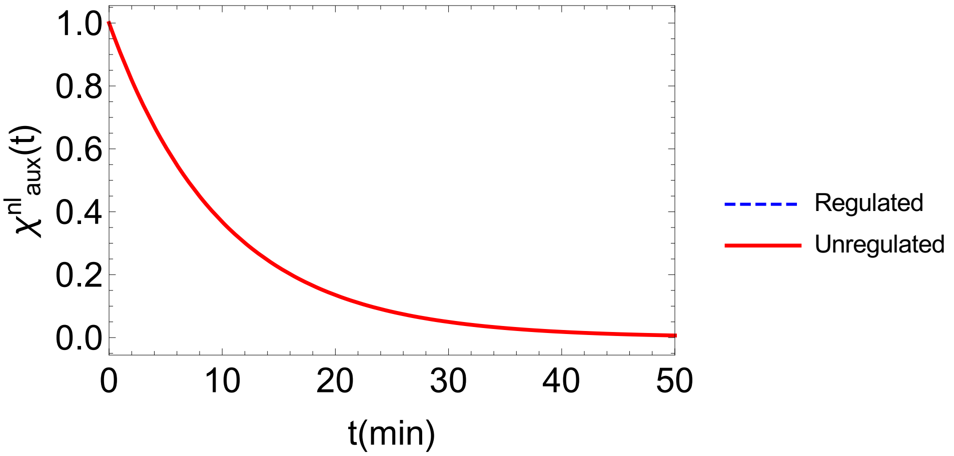

```
Plot[{chinlreg[[1]], chinl[[1]]}, {t, 0, 700}, PlotRange → {{0, 700}, All},
 PlotLegends → {"Regulated", "Unregulated"}, Frame → True,
 FrameLabel → {"t(min)", "χ^nl_IAA(t)"}, PlotStyle → {{Blue, Dashed}, {Red, Thick}}]
```

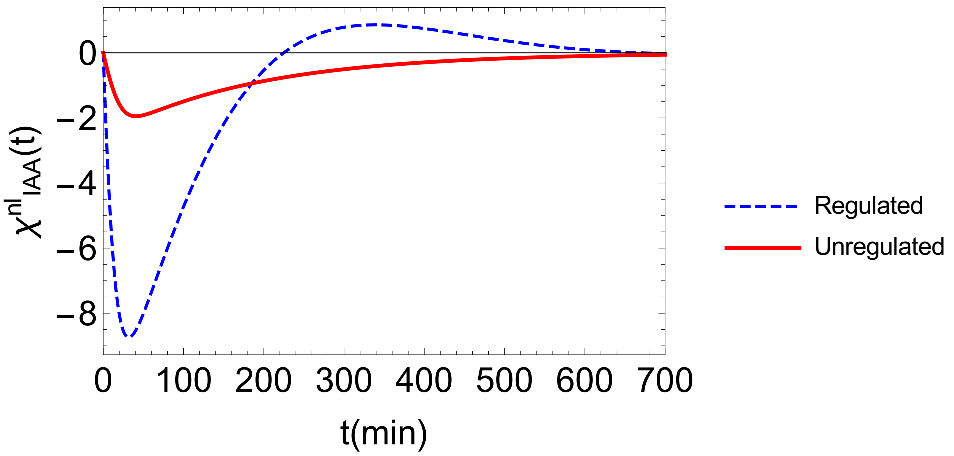


```
Plot[{chinlreg[[3]], chinl[[3]]}, {t, 0, 700}, PlotRange → {{0, 700}, All},
 PlotLegends → {"ARF nonlinear response regulated w=0.02", "unregulated"},
 Frame → True, FrameLabel → {"t(min)", "χ^nl_ARF(t)"},
 PlotStyle → {{Blue, Dashed}, {Red, Thick}}]
```

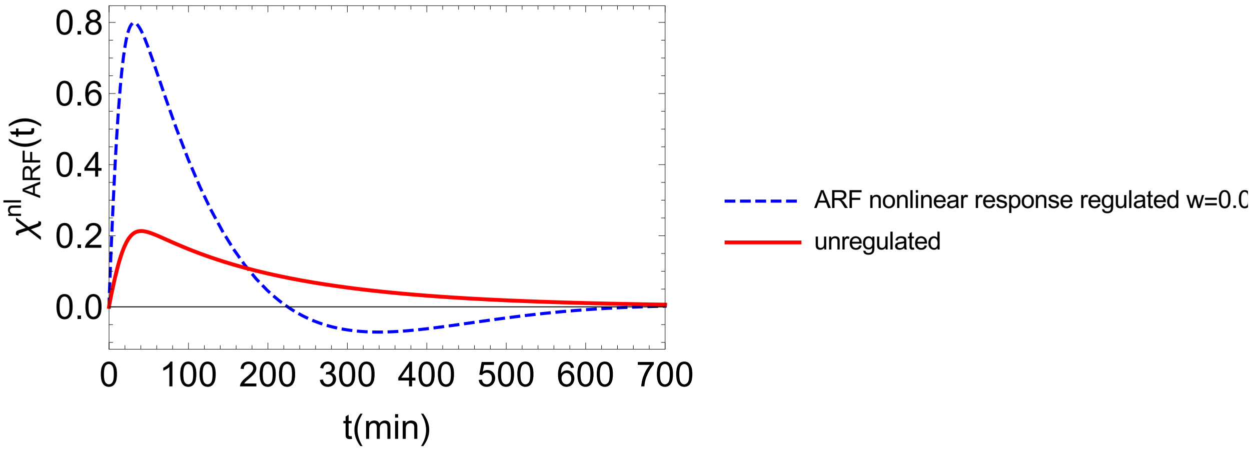

Resilience times for S_{auxin}=0.02, with and without regulation

We hereby calculate the resilience time δt as the time for nonlinear responses to decrease from their maximum value to 10% of that value.

With regulation

```
listchinlreg = Table[Abs[chinlreg], {t, 0, 1000, dt}];
posmax = Table[
   Flatten@Position[listchinlreg[[All, i]], Max[listchinlreg[[All, i]]]], {i, 3}];
listchinlreg01 = Table[Drop[listchinlreg[[All, i]], posmax[[i, 1]]], {i, 3}];
pos01max =
  Table[Flatten@Position[listchinlreg[[All, i]], Select[listchinlreg01[[i]],
       # < 0.1 * Max[listchinlreg[[All, i]]] &, 1][[1]]], {i, 3}];
```

IAA resilience time with regulation

```
dtIAAw0n = Table[{t, Abs[chinlreg[[1]]]}, {t, 0, 400, dt}][[pos01max[[1, 1]], 1]] -
  Table[{t, Abs[chinlreg[[1]]]}, {t, 0, 400, dt}][[posmax[[1, 1]], 1]]
```

```
155.7
```

ARF resilience time with regulation

```
dtARFw0n = Table[{t, Abs[chinlreg[[3]]]}, {t, 0, 400, dt}][[pos01max[[3, 1]], 1]] -
  Table[{t, Abs[chinlreg[[3]]]}, {t, 0, 400, dt}][[posmax[[3, 1]], 1]]
```

```
153.3
```

Without regulation

```
listchinl = Table[Abs[chinl], {t, 0, 1000, dt}];
posmax =
  Table[Flatten@Position[listchinl[[All, i]], Max[listchinl[[All, i]]]], {i, 3}];
listchinl01 = Table[Drop[listchinl[[All, i]], posmax[[i, 1]]], {i, 3}];
pos01max = Table[Flatten@Position[listchinl[[All, i]],
      Select[listchinl01[[i]], # < 0.1 * Max[listchinl[[All, i]]] &, 1][[1]]], {i, 3}];
```

IAA resilience time without regulation

```
dtIAAw0c = Table[{t, Abs[chinl[[1]]]}, {t, 0, 1000, dt}][[pos01max[[1, 1]], 1]] -
  Table[{t, Abs[chinl[[1]]]}, {t, 0, 1000, dt}][[posmax[[1, 1]], 1]]
```

```
435.3
```

ARF resilience time without regulation

```
dtARFw0c = Table[{t, Abs[chinl[[3]]]}, {t, 0, 1000, dt}][[pos01max[[3, 1]], 1]] -
  Table[{t, Abs[chinl[[3]]]}, {t, 0, 1000, dt}][[posmax[[3, 1]], 1]]
```

```
431.6
```

Resilience times for S_{auxin}=0.2, with and without regulation

We consider $S_{auxin} = 0.2$

```
w1 = 0.2;
```

We compute the corresponding steady states

```
initnegw1 = Block[{w = w1},
   NSolve[{((piI * Rss - 2 * alphaII * IAAss ^ 2 -
            alphaIA * IAAss * ARFss - dIAx * ARFIAAss - dIIx * IAA2ss - dIx * IAAss) /.
          {aux[t] → auxss} /. paramreg) == 0, IAAss > 0.}, IAAss]];
initconstw1 = Block[{w = w1}, NSolve[{((piI * Rssconst - 2 * alphaII * IAAss ^ 2 -
            alphaIA * IAAss * ARFss - dIAx * ARFIAAss - dIIx * IAA2ss - dIx * IAAss) /.
          {aux[t] → auxss} /. param) == 0, IAAss > 0.}, IAAss]];
```

We use the steady states as initial conditions, with an additive perturbation in auxin as previously

```
icsneg = Table[(vt[[i]] /. {t → tinit}) == Block[{w = w1},
     (ss[[i]] + vecpert[[i]]) /. initnegw1[[1]] /. paramreg], {i, nspecies}];
icsconst = Table[(vt[[i]] /. {t → tinit}) == Block[{w = w1},
     (ssconst[[i]] + vecpert[[i]]) /. initconstw1[[1]] /. param], {i, nspecies}];
```

We solve the system of ODE

```
eqssysneg = Join[eqspertneg, icsneg];
eqssysconst = Join[eqspertconst, icsconst];
Block[{ tinit = 0., tfin = 10 000., w = w1},
 nsolw1 = NDSolve[eqssysneg, vt, {t, tinit, tfin}];
 csolw1 = NDSolve[eqssysconst, vt, {t, tinit, tfin}];];
```

We compute the corresponding resilience times for IAA and ARF following the same steps as for S_{auxin}= 0.02, starting from the definition of the nonlinear response

```
chinlregw1 = Block[{w = w1},
   ((Evaluate[vt /. nsolw1[[1]]] - ss /. initnegw1[[1]]) / (10 * w * τ)) /. paramreg];
chinlw1 = Block[{w = w1},
   ((Evaluate[vt /. csolw1[[1]]] - ssconst /. initconstw1[[1]]) / (10 * w * τ)) /. param];
```

With regulation

```
listchinlreg = Table[Abs[chinlregw1], {t, 0, 400, dt}];
posmax = Table[
   Flatten@Position[listchinlreg[[All, i]], Max[listchinlreg[[All, i]]]], {i, 3}];
listchinlreg01 = Table[Drop[listchinlreg[[All, i]], posmax[[i, 1]]], {i, 3}];
pos01max =
  Table[Flatten@Position[listchinlreg[[All, i]], Select[listchinlreg01[[i]],
       # < 0.1 * Max[listchinlreg[[All, i]]] &, 1][[1]]], {i, 3}];
```

IAA resilience time with regulation

```
dtIAAw1n = Table[{t, Abs[chinlregw1[[1]]]}, {t, 0, 400, dt}][[pos01max[[1, 1]], 1]] -
  Table[{t, Abs[chinlregw1[[1]]]}, {t, 0, 400, dt}][[posmax[[1, 1]], 1]]
```

```
68.1
```

ARF resilience time with regulation

```
dtARFw1n = Table[{t, Abs[chinlregw1[[3]]]}, {t, 0, 400, dt}][[pos01max[[3, 1]], 1]] -
  Table[{t, Abs[chinlregw1[[3]]]}, {t, 0, 400, dt}][[posmax[[3, 1]], 1]]
```

```
67.6
```

Without regulation

```
listchinl = Table[Abs[chinlw1], {t, 0, 400, dt}];
posmax =
  Table[Flatten@Position[listchinl[[All, i]], Max[listchinl[[All, i]]]], {i, 3}];
listchinl01 = Table[Drop[listchinl[[All, i]], posmax[[i, 1]]], {i, 3}];
pos01max = Table[Flatten@Position[listchinl[[All, i]],
      Select[listchinl01[[i]], # < 0.1 * Max[listchinl[[All, i]]] &, 1][[1]]], {i, 3}];
```

IAA resilience time without regulation

```
dtIAAw1c = Table[{t, Abs[chinlw1[[1]]]}, {t, 0, 400, dt}][[pos01max[[1, 1]], 1]] -
  Table[{t, Abs[chinlw1[[1]]]}, {t, 0, 400, dt}][[posmax[[1, 1]], 1]]
```

```
316.2
```

ARF resilience time without regulation

```
dtARFw1c = Table[{t, Abs[chinlw1[[3]]]}, {t, 0, 400, dt}][[pos01max[[3, 1]], 1]] -
  Table[{t, Abs[chinlw1[[3]]]}, {t, 0, 400, dt}][[posmax[[3, 1]], 1]]
```

```
315.6
```

Plots of nonlinear responses for Sauxin=0.2

```
Plot[{chinlregw1[[6]], chinlw1[[6]]}, {t, 0, 100},
 PlotLegends → {"Regulated", "Unregulated"}, PlotRange → {{0, 50}, All}, Frame → True,
 FrameLabel → {"t(min)", "χ^nl_aux(t)"}, PlotStyle → {{Blue, Dashed}, {Red, Thick}}]
```

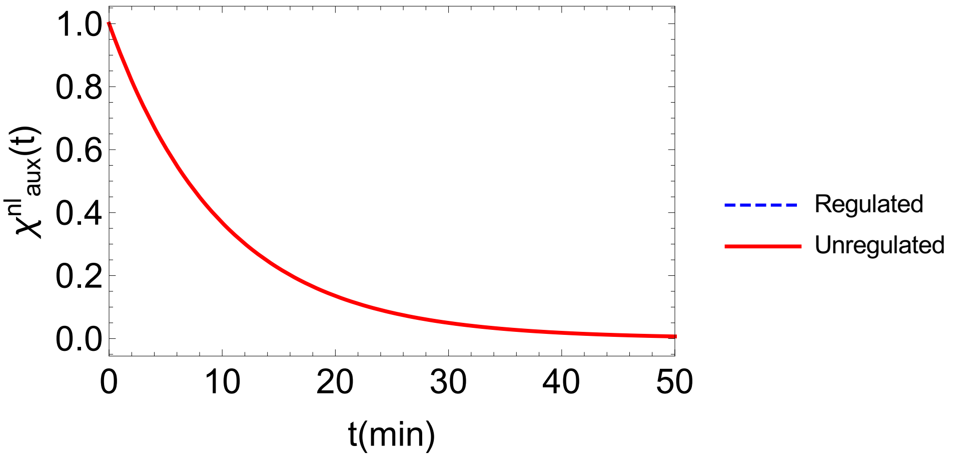

```
Plot[{chinlregw1[[1]], chinlw1[[1]]}, {t, 0, 400}, PlotRange → {{0, 400}, All},
 PlotLegends → {"Regulated", "Unregulated"}, Frame → True,
 FrameLabel → {"t(min)", "χ^nl_IAA(t)"}, PlotStyle → {{Blue, Dashed}, {Red, Thick}}]
```

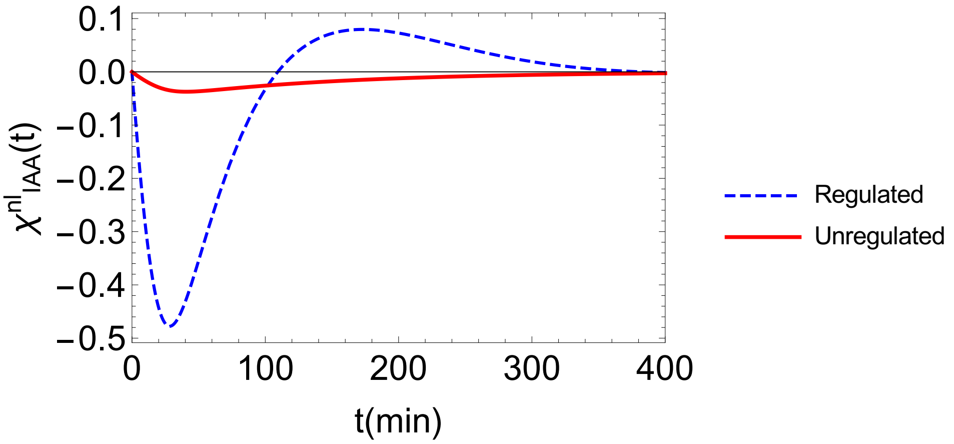


```
Plot[{chinlregw1[[3]], chinlw1[[3]]}, {t, 0, 400}, PlotRange → {{0, 400}, All},
 PlotLegends → {"Regulated", "Unregulated"}, Frame → True,
 FrameLabel → {"t(min)", "χ^nl_ARF(t)"}, PlotStyle → {{Blue, Dashed}, {Red, Thick}}]
```

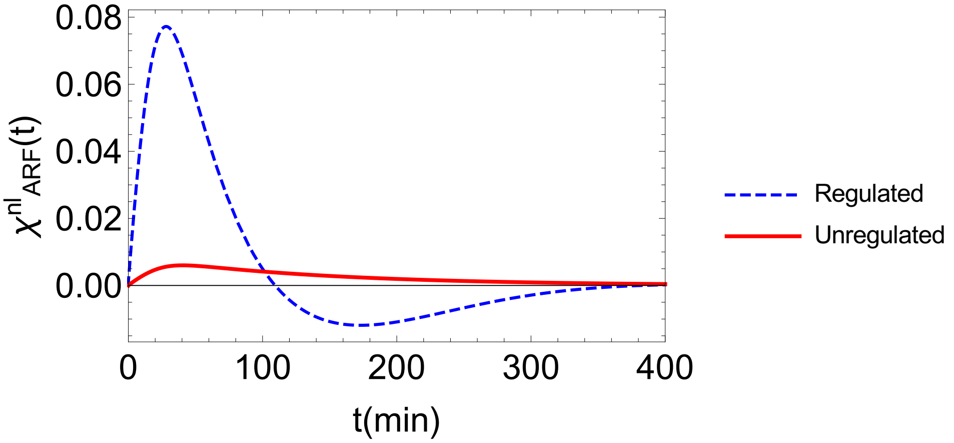

Steady state analysis

Definition of Vernoux model without IAA-homodimer

Here we are interested in assessing the effect on the statics and dynamics of the presence of IAA-homodimers, therefore we remove it. Hereby we define a version of the Vernoux model without IAA-homodimers

Parameters

```
paramnodimer = {piI → 1., dr → 0.007, dA → 0.003, dIA → 0.003, kIA → 1.,
   bd → 100.0, fA → 10., gammaI → 10., Kaux → 1. , KIA → 10, Ka → 1 * 1.3,
   fC → 10, f → 10, wA → 10, wI → 10, wD → 10., dI → 0.05 * 1.3, piA → 1.,
   kAm → 10., kprimeIA → 10.,  auxbasal → 0.11, dstarIA → 0.003, τ → 10};
```

Time variation

```
vtnd = {IAA[t], ARF[t], ARFIAA[t], R[t], aux[t]};
```

Vector of steady states

```
ssnd = {IAAss, ARFss, ARFIAAss, Rss, auxss};
replacessnd = Table[vtnd[[n]] → ssnd[[n]], {n, 1, nspecies - 1}];
```

Linear and nonlinear part of the dynamics and inclusion of parameters

```
Mnd = {{-dIx, 0, kprimeIA, piI, 0}, {0, -dA, kprimeIA + dIAx, 0, 0},
   {0, 0, - (kprimeIA + dIAx + dstarIA), 0, 0}, {0, 0, 0, -dr, 0}, {0, 0, 0, 0, -1 / τ}};
bnd = {{-kIA * IAA[t] * ARF[t] - f0}, {piA - kIA * IAA[t] * ARF[t]},
   {kIA * IAA[t] * ARF[t]}, {h}, {w}};
Mopnd = Mnd /. paramnodimer;
bopnd  = bnd /. paramnodimer;
RHSnd  = Mopnd.vtnd + bopnd;
```

Jacobian of the dynamics

We hereby linearize the dynamics around the steady state and define the Jacobian matrices for the negative feedback case, the unregulated case and the model without IAA-homodimers.

```
Clear[IAAss, IAA2ss, ARFss, ARFIAAss, Rss, auxss, ss, ssnd];
ss = {IAAss, IAA2ss, ARFss, ARFIAAss, Rss, auxss};
ssnd = {IAAss, ARFss, ARFIAAss, Rss, auxss};
replacess = Table[vt[[n]] → ss[[n]], {n, 1, nspecies}];
replacessnd = Table[vtnd[[n]] → ssnd[[n]], {n, 1, nspecies - 1}];
RHSssneg = RHSneg /. replacess;
RHSssnd = RHSnd /. replacessnd;
RHSssconst = RHSconst /. replacess;
RHSNDssF[IAAss, IAA2ss, ARFss, ARFIAAss, Rss, auxss] := RHSssneg;
RHSNDssFconst[IAAss, IAA2ss, ARFss, ARFIAAss, Rss, auxss] := RHSssconst;
RHSNDssFnd[IAAss, ARFss, ARFIAAss, Rss, auxss] := RHSssnd;
```

Hereby the definition of the Jacobian matrices for the negative feedback, the unregulated case and the

case without IAA dimers.

```
Jacobnd =
  Table[D[RHSNDssFnd[IAAss, ARFss, ARFIAAss, Rss, auxss][[m]][[1]], ssnd[[n]]],
   {m, 1, nspecies - 1}, {n, 1, nspecies - 1}];
Jacobneg = Table[D[RHSNDssF[IAAss, IAA2ss, ARFss, ARFIAAss, Rss, auxss][[m]][[1]],
     ss[[n]]], {m, 1, nspecies}, {n, 1, nspecies}];
Jacobconst = Table[D[RHSNDssFconst[IAAss, IAA2ss, ARFss, ARFIAAss, Rss, auxss][[m]][[
      1]], ss[[n]]], {m, 1, nspecies}, {n, 1, nspecies}];
```

Steady state concentrations with varying S_{auxin}

Hereby we re-introduce the expressions for the steady states

```
IAA2ss = kII * IAAss ^ 2 / (kprimeII + dstarII + dIIx) /. {aux[t] → auxss};
ARFIAAss = kIA * IAAss * ARFss / (kprimeIA + dstarIA + dIAx) /. {aux[t] → auxss};
Rss =
  (h /. {IAA[t] → IAAss, ARF[t] → ARFss, ARFIAA[t] → ARFIAAss, aux[t] → auxss}) / dr;
alphaIA = kIA * dstarIA / (kprimeIA + dstarIA + dIAx) /. {aux[t] → auxss};
alphaII = kII * dstarII / (kprimeII + dstarII + dIIx) /. {aux[t] → auxss};
ARFss = piA / (dA + alphaIA * IAAss) /. {aux[t] → auxss};
auxss = w * τ;
```

We define the range of variation of auxin influx

```
lwwinit = -10.;
lwwfin = 10.;
lwwstep = 0.1;
wwinit = Exp[lwwinit];
wwfin = Exp[lwwfin];
```

We initialize the steady states by solving the self-consistent equation for IAAss

```
initneg =
  Block[{w = wwinit}, NSolve[{((piI * Rss - 2 * alphaII * IAAss ^ 2 - alphaIA * IAAss * ARFss -
           dIAx * ARFIAAss - dIIx * IAA2ss - dIx * IAAss) /.
         {aux[t] → auxss} /. paramreg) == 0, IAAss > 0.}, IAAss]];
initconst = Block[{w = wwinit}, NSolve[{((piI * Rssconst - 2 * alphaII * IAAss ^ 2 -
           alphaIA * IAAss * ARFss - dIAx * ARFIAAss - dIIx * IAA2ss - dIx * IAAss) /.
         {aux[t] → auxss} /. param) == 0, IAAss > 0.}, IAAss]];
initnd = Block[{w = wwinit}, NSolve[{((piI * Rss - alphaIA * IAAss * ARFss -
            dIAx * ARFIAAss - dIx * IAAss - f0) /. {aux[t] → auxss} /. paramnodimer /.
         {IAA2ss → 0} /. paramnodimer) == 0, IAAss > 0.}, IAAss]];
```

Hereby we define the lists of the steady states for IAA, ARF and auxin for the negative feedback and the unregulated case, respectively

```
listpneg = {};
listpconst = {};
listpnd = {};
listtarfneg = {};
listtarfconst = {};
listtarfnd = {};
listauxneg = {};
listauxnd = {};
listauxconst = {};
```

We start varying the auxin influx and find the solution to the self-consistency equation. We use this result to recompute all the steady states at each step

```
For[lww = lwwinit, lww ≤ lwwfin, lww += lwwstep, ww = Exp[lww];
 initcondsneg = IAAss /. paramreg /. initneg[[1]];
 initcondsconst = IAAss /. param /. initconst[[1]];
 initcondsnd = IAAss /. paramnodimer /. initnd[[1]];
 newnd = Block[{w = ww}, Assuming[IAAss > 0.,
    FindRoot[((piI * Rss - alphaIA * IAAss * ARFss - dIAx * ARFIAAss - dIx * IAAss - f0) /.
         {aux[t] → auxss} /. paramnodimer) == 0, {IAAss, initcondsnd}]]];
 newneg = Block[{w = ww}, Assuming[IAAss > 0., FindRoot[
     ((piI * Rss - 2 * alphaII * IAAss ^ 2 - alphaIA * IAAss * ARFss -
           dIAx * ARFIAAss - dIIx * IAA2ss - dIx * IAAss) /.
         {aux[t] → auxss} /. paramreg) == 0, {IAAss, initcondsneg}]]];
 newconst = Block[{w = ww}, Assuming[IAAss > 0., FindRoot[
     ((piI * Rssconst - 2 * alphaII * IAAss ^ 2 - alphaIA * IAAss * ARFss -
           dIAx * ARFIAAss - dIIx * IAA2ss - dIx * IAAss) /.
         {aux[t] → auxss} /. param) == 0, {IAAss, initcondsconst}]]];
 listpneg = Append[listpneg, {ww, IAAss} /. newneg];
 listpnd = Append[listpnd, {ww, IAAss} /. newnd];
 listpconst = Append[listpconst, {ww, IAAss} /. newconst];
 listtarfneg = Append[listtarfneg, {ww, ARFss} /. paramreg /. newneg /. {w → ww}];
 listtarfnd = Append[listtarfnd, {ww, ARFss} /. paramnodimer /. newnd /. {w → ww}];
 listtarfconst = Append[listtarfconst, {ww, ARFss} /. param /. newconst /. {w → ww}];
 listauxneg = Append[listauxneg, {ww, auxss} /. paramreg /. newneg /. {w → ww}];
 listauxnd = Append[listauxnd, {ww, auxss} /. paramnodimer /. newnd /. {w → ww}];
 listauxconst = Append[listauxconst, {ww, auxss} /. param /. newconst /. {w → ww}];
 initneg[[1]] = newneg;
 initconst[[1]] = newconst;
 initnd[[1]] = newnd;
]
```

Plots of comparison between the Vernoux models with and without IAA-homodimer

```
SetOptions[ListLinePlot, BaseStyle → FontSize → 18];
```

```
ListLinePlot[{listauxneg, listauxnd}, PlotRange → {{0, 0.5}, {0, 5}}, Frame → True,
 FrameLabel → {"\!\(\*SubscriptBox[\(S\), \(auxin\)]\)(nM/min)", "auxin(nM)"},
 PlotStyle → {{Blue, Dashed}, {Green, Thick}},
 PlotLegends → {"With dimer", "Without dimer"}]
```

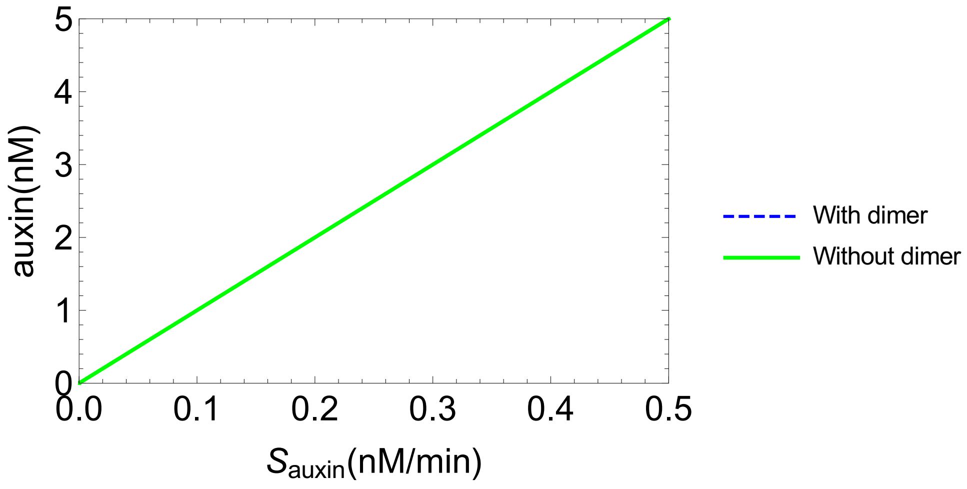


```
ListLinePlot[{listpneg[[1 ;; 125, All]], listpnd[[1 ;; 125, All]]},
 PlotRange → {{0., 0.5}, {0, 250}}, Frame → True,
 FrameLabel → {"\!\(\*SubscriptBox[\(S\), \(auxin\)]\)(nM/min)", "IAA(nM)"},
 PlotStyle → {{Blue, Dashed}, {Green, Thick}},
 PlotLegends → {"With dimer", "Without dimer"}]
```

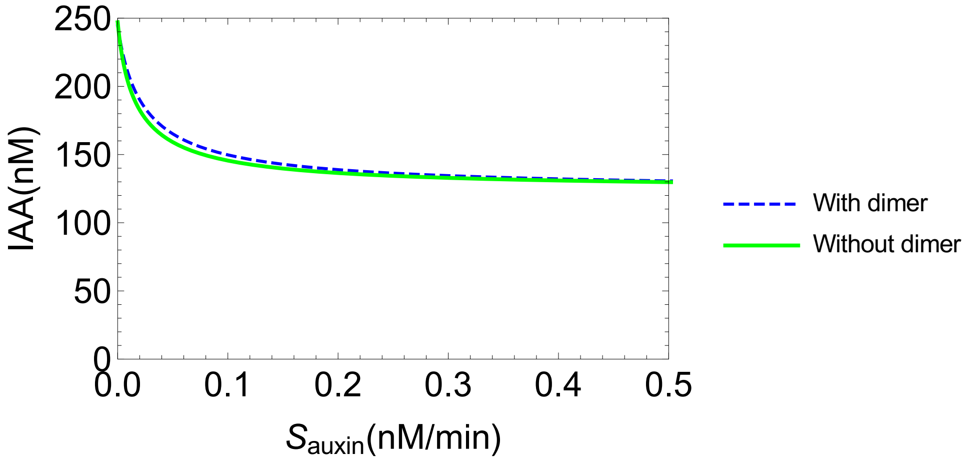

```
ListLinePlot[{listtarfneg[[1 ;; 125, All]], listtarfnd[[1 ;; 125, All]]},
 PlotRange → {{0, 0.5}, {0, 40}}, Frame → True,
 FrameLabel → {"\!\(\*SubscriptBox[\(S\), \(auxin\)]\)(nM/min)", "ARF(nM)"},
 PlotStyle → {{Blue, Dashed}, {Green, Thick}},
 PlotLegends → {"With dimer", "Without dimer"}]
```

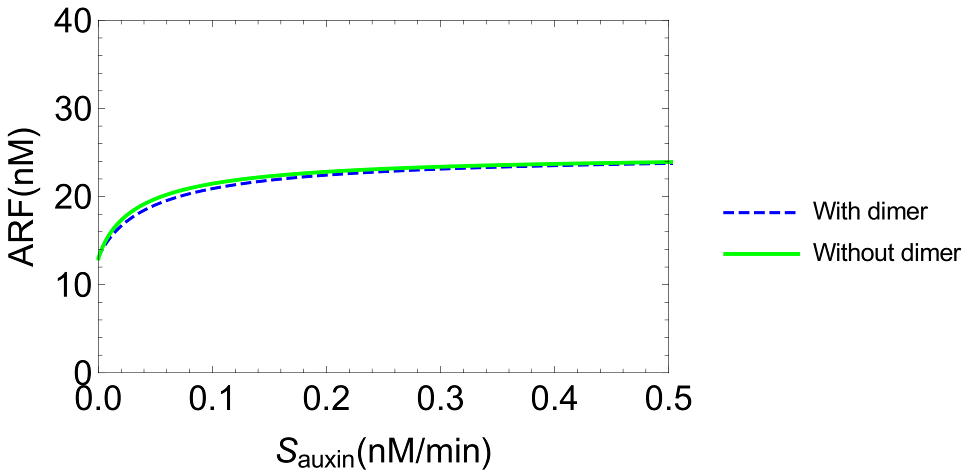

Plots of comparison between regulated and unregulated model

```
plotauxss = ListLinePlot[{listauxneg[[1 ;; 125, All]], listauxconst[[1 ;; 125, All]]},
  PlotRange → {{0, 0.5}, {0, 5}}, Frame → True,
  FrameLabel → {"\!\(\*SubscriptBox[\(S\), \(auxin\)]\)(nM/min)", "auxin(nM)"},
  PlotStyle → {{Blue, Dashed}, {Red, Thick}},
  PlotLegends → {"Regulated", "Unregulated"}]
```

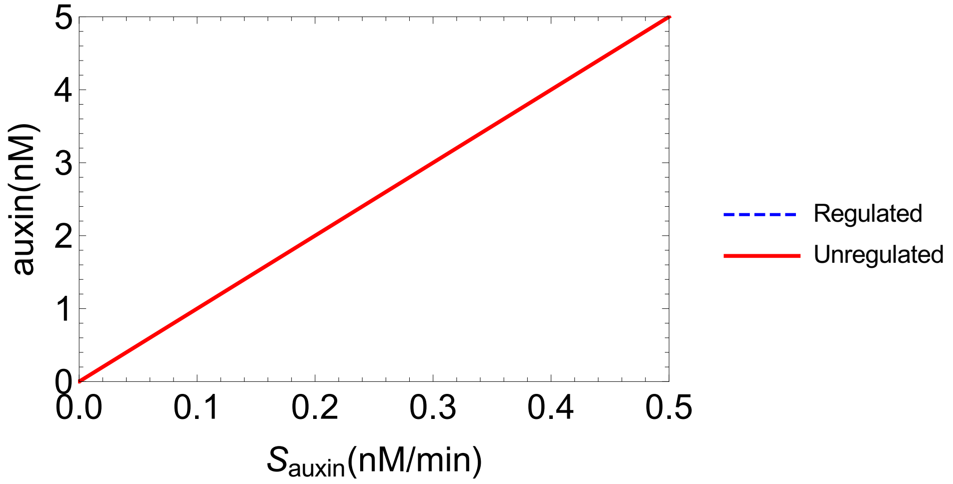

```
plotIAAss = ListLinePlot[{listpneg[[1 ;; 125, All]], listpconst[[1 ;; 125, All]]},
  PlotRange → {{0, 0.5}, All}, Frame → True,
  FrameLabel → {"\!\(\*SubscriptBox[\(S\), \(auxin\)]\)(nM/min)", "IAA(nM)"},
  PlotStyle → {{Blue, Dashed}, {Red, Thick}},
  PlotLegends → {"Regulated", "Unregulated"}]
```

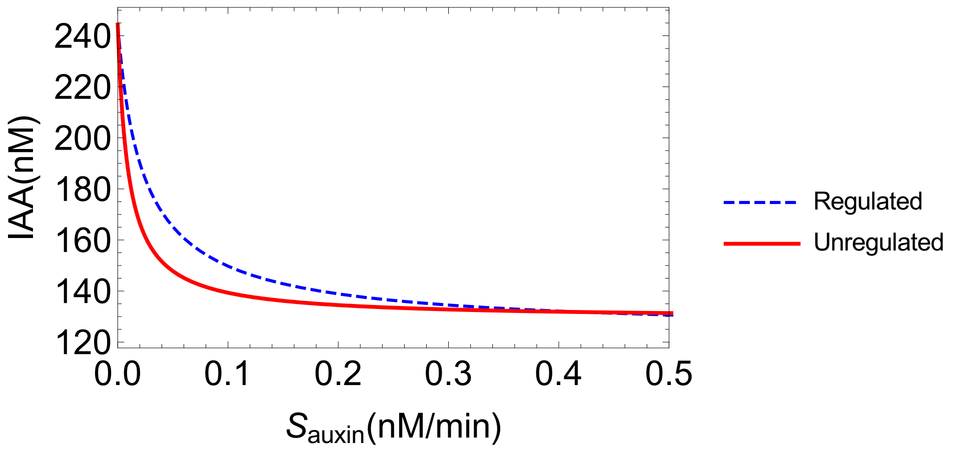


```
plotARFss =
 ListLinePlot[{listtarfneg[[1 ;; 125, All]], listtarfconst[[1 ;; 125, All]]},
  PlotRange → {{0, 0.5}, All}, Frame → True,
  FrameLabel → {"\!\(\*SubscriptBox[\(S\), \(auxin\)]\)(nM/min)", "ARF(nM)"},
  PlotStyle → {{Blue, Dashed}, {Red, Thick}},
  PlotLegends → {"Regulated", "Unregulated"}]
```

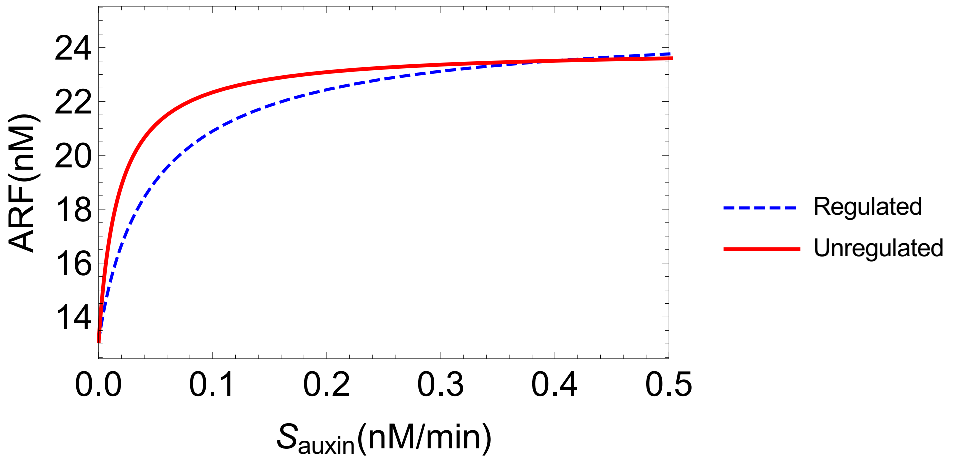

Linear Response analysis

We hereby consider $S_{auxin} = 0.02$ and compute the corresponding steady states

```
w0 = 0.02;
initneg =
  Block[{w = w0}, NSolve[{((piI * Rss - 2 * alphaII * IAAss ^ 2 - alphaIA * IAAss * ARFss -
          dIAx * ARFIAAss - dIIx * IAA2ss - dIx * IAAss) /.
        {aux[t] → auxss} /. paramreg) == 0, IAAss > 0.}, IAAss]];
initconst = Block[{w = w0}, NSolve[{((piI * Rssconst - 2 * alphaII * IAAss ^ 2 -
          alphaIA * IAAss * ARFss - dIAx * ARFIAAss - dIIx * IAA2ss - dIx * IAAss) /.
        {aux[t] → auxss} /. param) == 0, IAAss > 0.}, IAAss]];
initnd = Block[{w = w0}, NSolve[{((piI * Rss - alphaIA * IAAss * ARFss -
          dIAx * ARFIAAss - dIx * IAAss - f0) /.
        {aux[t] → auxss, c → 40.} /. paramnodimer) == 0, IAAss > 0.}, IAAss]];
```

We hereby verify that the eigenvalues of the Jacobian are mostly real and they are all negative (this applies to the real part when complex), i.e. the time evolution is given by a decay

```
J = Jacobneg /. paramreg /. initneg[[1]] /. w → w0;
J1 = Jacobconst /. param /. initconst[[1]] /. w → w0;
Jnd = Jacobnd /. paramnodimer /. initnd[[1]] /. w → w0;
Chop[Eigenvalues[J]]
Chop[Eigenvalues[Jnd]]
Chop[Eigenvalues[J1]]
```

```
{-792.785, -194.832, -0.1,
 -0.00675823 + 0.00709818 i, -0.00675823 - 0.00709818 i, -0.003}

{-209.964, -0.1, -0.0654 + 0.0244978 i, -0.0654 - 0.0244978 i, -0.003}

{-701.383, -170.622, -0.1, -0.007, -0.00539583, -0.003}
```

To find the linear responses, one takes the exponential of the Jacobian x time

```
RT = MatrixExp[J * t];
RT1 = MatrixExp[J1 * t];
RTnd = MatrixExp[Jnd * t];
```

Plots of comparison of linear responses with and without IAA-homodimer (we plot the components which represent the response, in the linear regime, of aux, IAA, ARF concentrations to a small perturbation in aux)

```
SetOptions[ListLinePlot, BaseStyle → {FontSize → 18, FontFamily → Helvetica}];
SetOptions[LogLinearPlot, BaseStyle → FontSize → 18];
```

```
Plot[{RT[[6, 6]], RTnd[[5, 5]]}, {t, 0, 50}, PlotRange → {{0, 50}, All},
 PlotStyle → {{Blue, Dashed}, {Green, Thick}}, Frame → True,
 FrameLabel -> {"t(min)", "χaux(t)"}, PlotLegends → {"With dimer", "Without dimer"}]
```

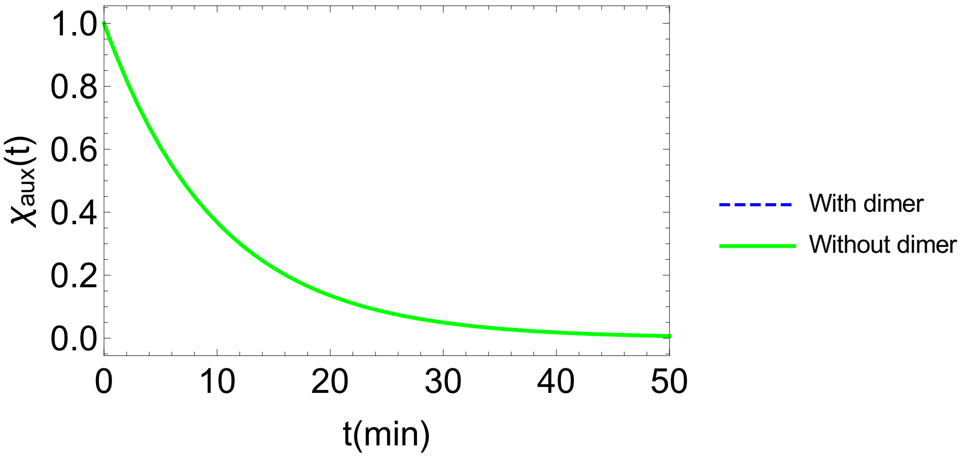


```
Plot[{RT[[1, 6]], Chop[RTnd[[1, 5]]]}, {t, 0, 250}, PlotRange → {{0, 250}, All},
 PlotStyle → {{Blue, Dashed}, {Green, Thick}}, Frame → True,
 FrameLabel -> {"t(min)", "χIAA(t)"}, PlotLegends → {"With dimer", "Without dimer"}]
```

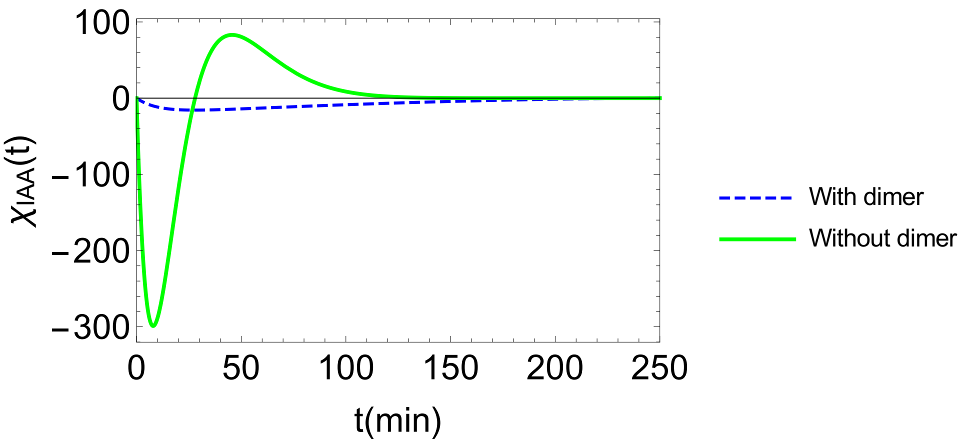

```
Plot[{RT[[3, 6]], Chop[RTnd[[2, 5]]]}, {t, 0, 250}, PlotRange → {{0, 250}, All},
 PlotStyle → {{Blue, Dashed}, {Green, Thick}}, Frame → True,
 FrameLabel -> {"t(min)", "χARF(t)"}, PlotLegends → {"With dimer", "Without dimer"}]
```

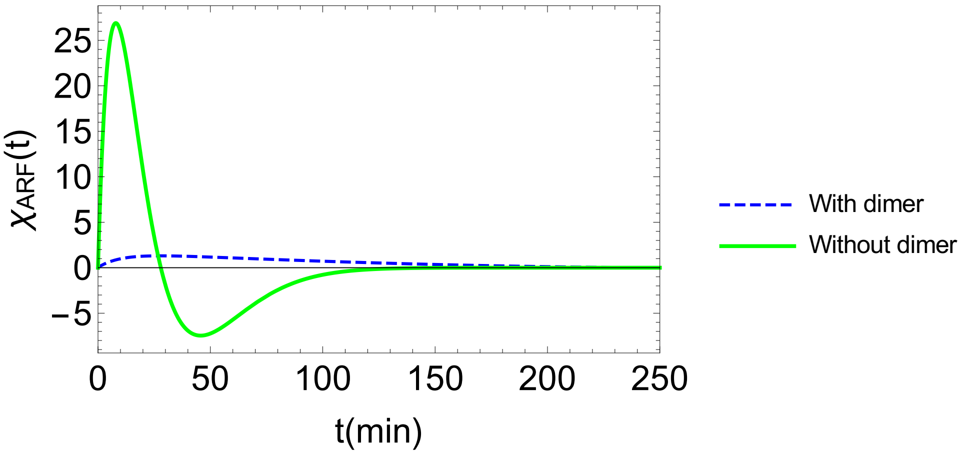

Plots of comparison of linear responses with and without regulation

```
Plot[{RT[[6, 6]], RT1[[6, 6]]}, {t, 0, 50}, PlotRange → {{0, 50}, All},
 PlotStyle → {{Blue, Dashed}, {Red, Thick}}, Frame → True,
 FrameLabel -> {"t(min)", "χaux(t)"}, PlotLegends → {"Regulated", "Unregulated"}]
```

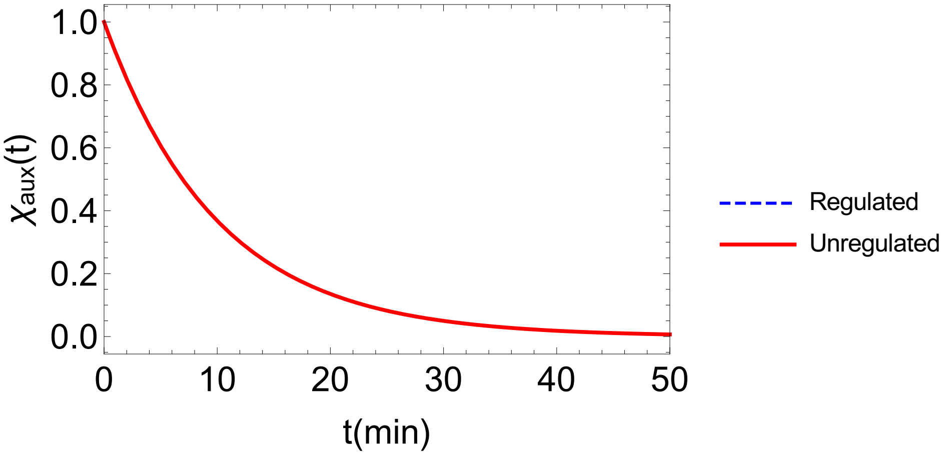

```
Plot[{RT[[1, 6]], RT1[[1, 6]]}, {t, 0, 800}, PlotRange → {{0, 800}, All},
 PlotStyle → {{Blue, Dashed}, {Red, Thick}}, Frame → True,
 FrameLabel -> {"t(min)", "χIAA(t)"}, PlotLegends → {"Regulated", "Unregulated"}]
```

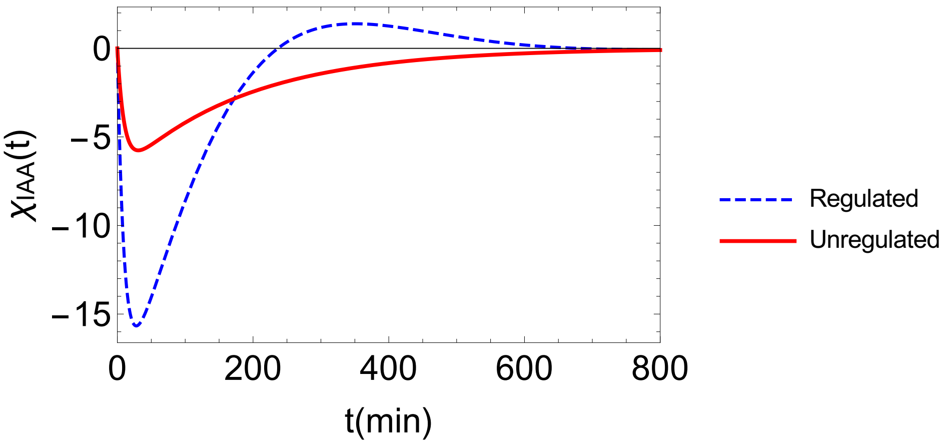


```
Plot[{RT[[3, 6]], RT1[[3, 6]]}, {t, 0, 800}, PlotRange → {{0, 800}, All},
 PlotStyle → {{Blue, Dashed}, {Red, Thick}}, Frame → True,
 FrameLabel -> {"t(min)", "χARF(t)"}, PlotLegends → {"Regulated", "Unregulated"}]
```

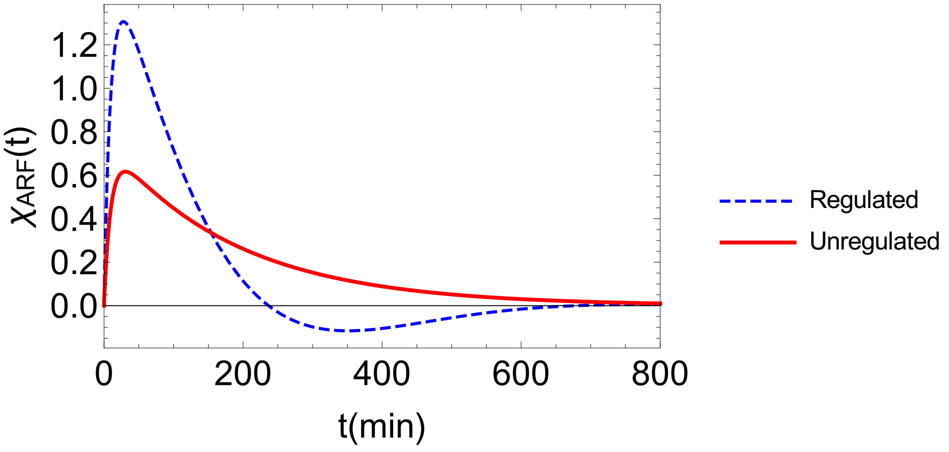

Resilience times for S_{auxin}=0.02, with and without regulation

We hereby calculate the resilience time δt as the time for the linear responses to decrease from their maximum value to 10% of that value

With regulation

```
listchireg = Table[Abs[RT[[All, 6]]], {t, 0, 1000, dt}];
posmax =
  Table[Flatten@Position[listchireg[[All, i]], Max[listchireg[[All, i]]]], {i, 3}];
listchireg01 = Table[Drop[listchireg[[All, i]], posmax[[i, 1]]], {i, 3}];
pos01max = Table[Flatten@Position[listchireg[[All, i]], Select[
       listchireg01[[i]], # < 0.1 * Max[listchireg[[All, i]]] &, 1][[1]]], {i, 3}];
```

IAA resilience time with regulation

```
dtIAAw0n = Table[{t, Abs[RT[[1, 6]]]}, {t, 0, 400, dt}][[pos01max[[1, 1]], 1]] -
  Table[{t, Abs[RT[[1, 6]]]}, {t, 0, 400, dt}][[posmax[[1, 1]], 1]]
167.8
```

ARF resilience time with regulation

```
dtARFw0n = Table[{t, Abs[RT[[3, 6]]]}, {t, 0, 400, dt}][[pos01max[[3, 1]], 1]] -
  Table[{t, Abs[RT[[3, 6]]]}, {t, 0, 400, dt}][[posmax[[3, 1]], 1]]
168.
```

Without regulation

```
listchi = Table[Abs[RT1[[All, 6]]], {t, 0, 1000, dt}];
posmax =
  Table[Flatten@Position[listchi[[All, i]], Max[listchi[[All, i]]]], {i, 3}];
listchi01 = Table[Drop[listchi[[All, i]], posmax[[i, 1]]], {i, 3}];
pos01max = Table[Flatten@Position[listchi[[All, i]],
     Select[listchi01[[i]], # < 0.1 * Max[listchi[[All, i]]] &, 1][[1]]], {i, 3}];
```

IAA resilience time without regulation

```
dtIAAw0c = Table[{t, Abs[RT1[[1, 6]]]}, {t, 0, 1000, dt}][[pos01max[[1, 1]], 1]] -
  Table[{t, Abs[RT1[[1, 6]]]}, {t, 0, 1000, dt}][[posmax[[1, 1]], 1]]
437.
```

ARF resilience time without regulation

```
dtARFw0c = Table[{t, Abs[RT1[[3, 6]]]}, {t, 0, 1000, dt}][[pos01max[[3, 1]], 1]] -
  Table[{t, Abs[RT1[[3, 6]]]}, {t, 0, 1000, dt}][[posmax[[3, 1]], 1]]
437.
```

Resilience times for S_{auxin}=0.2, with and without regulation

We hereby calculate the resilience time δt as the time for the linear responses to decrease from their maximum value to 10% of that value

We first estimate the linear responses following the same steps as for S_{auxin}=0.02

```
w1 = 0.2;
initnegw1 =
  Block[{w = w1}, NSolve[{((piI * Rss - 2 * alphaII * IAAss ^ 2 - alphaIA * IAAss * ARFss -
          dIAx * ARFIAAss - dIIx * IAA2ss - dIx * IAAss) /.
        {aux[t] → auxss} /. paramreg) == 0, IAAss > 0.}, IAAss]];
initconstw1 = Block[{w = w1}, NSolve[{((piI * Rssconst - 2 * alphaII * IAAss ^ 2 -
          alphaIA * IAAss * ARFss - dIAx * ARFIAAss - dIIx * IAA2ss - dIx * IAAss) /.
        {aux[t] → auxss} /. param) == 0, IAAss > 0.}, IAAss]];

J = Jacobneg /. paramreg /. initnegw1[[1]] /. w → w1;
J1 = Jacobconst /. param /. initconstw1[[1]] /. w → w1;
RT = MatrixExp[J * t];
RT1 = MatrixExp[J1 * t];
```

With regulation

```
listchiregw1 = Table[Abs[RT[[All, 6]]], {t, 0, 1000, dt}];
posmax = Table[
   Flatten@Position[listchiregw1[[All, i]], Max[listchiregw1[[All, i]]]], {i, 3}];
listchireg01w1 = Table[Drop[listchiregw1[[All, i]], posmax[[i, 1]]], {i, 3}];
pos01max =
  Table[Flatten@Position[listchiregw1[[All, i]], Select[listchireg01w1[[i]],
       # < 0.1 * Max[listchiregw1[[All, i]]] &, 1][[1]]], {i, 3}];
```

IAA resilience time with regulation

```
dtIAAw1n = Table[{t, Abs[RT[[1, 6]]]}, {t, 0, 1000, dt}][[pos01max[[1, 1]], 1]] -
  Table[{t, Abs[RT[[1, 6]]]}, {t, 0, 1000, dt}][[posmax[[1, 1]], 1]]
```

```
72.1
```

ARF resilience time with regulation

```
dtARFw1n = Table[{t, Abs[RT[[3, 6]]]}, {t, 0, 1000, dt}][[pos01max[[3, 1]], 1]] -
  Table[{t, Abs[RT[[3, 6]]]}, {t, 0, 1000, dt}][[posmax[[3, 1]], 1]]
```

```
72.2
```

Without regulation

```
listchiw1 = Table[Abs[RT1[[All, 6]]], {t, 0, 1000, dt}];
posmax =
  Table[Flatten@Position[listchiw1[[All, i]], Max[listchiw1[[All, i]]]], {i, 3}];
listchi01w1 = Table[Drop[listchiw1[[All, i]], posmax[[i, 1]]], {i, 3}];
pos01max = Table[Flatten@Position[listchiw1[[All, i]],
     Select[listchi01w1[[i]], # < 0.1 * Max[listchiw1[[All, i]]] &, 1][[1]]], {i, 3}];
```

IAA resilience time without regulation

```
dtIAAw1c = Table[{t, Abs[RT1[[1, 6]]]}, {t, 0, 1000, dt}][[pos01max[[1, 1]], 1]] -
  Table[{t, Abs[RT1[[1, 6]]]}, {t, 0, 1000, dt}][[posmax[[1, 1]], 1]]
```

```
315.9
```

ARF resilience time without regulation

```
dtARFw1c = Table[{t, Abs[RT1[[3, 6]]]}, {t, 0, 1000, dt}][[pos01max[[3, 1]], 1]] -
  Table[{t, Abs[RT1[[3, 6]]]}, {t, 0, 1000, dt}][[posmax[[3, 1]], 1]]
```

```
315.8
```

Full calibrated model

```
ClearAll["Global`*"]
SetOptions[Plot, BaseStyle → FontSize → 18];
SetOptions[ListPlot, BaseStyle → FontSize → 18];
SetOptions[ListLinePlot, BaseStyle → FontSize → 18];
```

System's setup

Variables' definition for time variation, steady state and derivatives

```
vt = {IAAm[t], IAAp[t], TIR1[t], auxTIR1[t],
   auxTIR1IAA[t], IAAstar[t], ARF[t], ARFIAA[t], ARF2[t], aux[t]};
ss = {IAAmss, IAApss, TIR1ss, auxTIR1ss, auxTIR1IAAss,
   IAAstarss, ARFSS, ARFIAAss, ARF2ss, auxss};
vdt = {{IAAm'[t]}, {IAAp'[t]}, {TIR1'[t]}, {auxTIR1'[t]}, {auxTIR1IAA'[t]},
   {IAAstar'[t]}, {ARF'[t]}, {ARFIAA'[t]}, {ARF2'[t]}, {aux'[t]}};
```

mRNA regulation functions

```
F1ssneg =
  ARFSS / thetaARF / (1 + ARFSS / thetaARF + ARFIAAss / thetaARFIAA + ARF2ss / thetaARF2);
F1neg = ARF[t] / thetaARF /
   (1 + ARF[t] / thetaARF + ARFIAA[t] / thetaARFIAA + ARF2[t] / thetaARF2);
F1ssconst = 0.0003;
F1const = 0.0003;
```

Initial conditions

```
ics = {{IAAm[tinit] == 0.}, {IAAp[tinit] == 0.}, {TIR1[tinit] == 18.51},
   {auxTIR1[tinit] == 0.}, {auxTIR1IAA[tinit] == 0.}, {IAAstar[tinit] == 0.},
   {ARF[tinit] == 10.}, {ARFIAA[tinit] == 0.}, {ARF2[tinit] == 0.}, {aux[tinit] == 0.}};
ics2 = {{IAAm[t /; t ≤ tinit] == 0.}, {IAAp[t /; t ≤ tinit] == 0.},
   {TIR1[t /; t ≤ tinit] == 18.51}, {auxTIR1[t /; t ≤ tinit] == 0.},
   {auxTIR1IAA[t /; t ≤ tinit] == 0.}, {IAAstar[t /; t ≤ tinit] == 0.},
   {ARF[t /; t ≤ tinit] == 10.}, {ARFIAA[t /; t ≤ tinit] == 0.},
   {ARF2[t /; t ≤ tinit] == 0.}, {aux[t /; t ≤ tinit] == 0.}};
```

Parameters

```
param = {muIAAm -> 0.003, lm → 0.009, delta → 4, la → 0.575, ld → 0.045, pa → 1.,
   pd → 0.072, ka → 0.00082, kd → 0.33, qa → 0.5, qd → 0.44 , muIAAstar → 0.1,
   muaux → 0.1, lambda1 → 0.48, thetaARF → 100., thetaARF2 → 100.,
   thetaARFIAA → 100.,  TIR1T → 100, ARFT → 200., muIAA → 0.003};
paramreg = {muIAAm -> 0.003, lm → 0.9, delta → 4, la → 5.75, ld → 0.045, pa → 1.,
   pd → 0.072, ka → 0.00082, kd → 0.33, qa → 0.5, qd → 0.44 , muIAAstar → 0.1,
   muaux → 0.1, lambda1 → 0.48, thetaARF → 100., thetaARF2 → 100.,
   thetaARFIAA → 100., TIR1T → 100, ARFT → 200., muIAA → 0.003};
```

ODEs definition -- linear part and non-linear part

```
M = {{-muIAAm, 0, 0, 0, 0, 0, 0, 0, 0, 0}, {delta, -muIAA, 0, 0, ld, 0, 0, pd, 0, 0},
   {0, 0, 0, kd, 0, 0, 0, 0, 0, 0}, {0, 0, 0, -kd, ld + lm, 0, 0, 0, 0, 0},
   {0, 0, 0, 0, -ld - lm, 0, 0, 0, 0, 0}, {0, 0, 0, 0, lm, -muIAAstar, 0, 0, 0, 0},
   {0, 0, 0, 0, 0, 0, 0, pd, 2 qd, 0}, {0, 0, 0, 0, 0, 0, 0, -pd, 0, 0},
   {0, 0, 0, 0, 0, 0, 0, 0, -qd, 0}, {0, 0, 0, kd, 0, 0, 0, 0, 0, -muaux}};
bneg = {{lambda1 * F1neg}, {-la * IAAp[t] * auxTIR1[t] - pa * IAAp[t] * ARF[t]},
   {-ka * aux[t] * TIR1[t]}, {ka * aux[t] * TIR1[t] - la * auxTIR1[t] * IAAp[t]},
   {la * IAAp[t] * auxTIR1[t]}, {0}, {-2 * qa * (ARF[t] ^ 2) - pa * ARF[t] * IAAp[t]},
   {pa * ARF[t] * IAAp[t]}, {qa * (ARF[t]) ^ 2}, {Sauxin - ka * aux[t] * TIR1[t]}};
bconst = {{lambda1 * F1const}, {-la * IAAp[t] * auxTIR1[t] - pa * IAAp[t] * ARF[t]},
   {-ka * aux[t] * TIR1[t]}, {ka * aux[t] * TIR1[t] - la * auxTIR1[t] * IAAp[t]},
   {la * IAAp[t] * auxTIR1[t]}, {0}, {-2 * qa * (ARF[t] ^ 2) - pa * ARF[t] * IAAp[t]},
   {pa * ARF[t] * IAAp[t]}, {qa * (ARF[t]) ^ 2}, {Sauxin - ka * aux[t] * TIR1[t]}};
```

Non-linear part with perturbation

```
bpertconst =
  {{lambda1 * F1const + lambda2 * F2}, {-la * IAAp[t] * auxTIR1[t] - pa * IAAp[t] * ARF[t]},
   {-ka * aux[t] * TIR1[t]}, {ka * aux[t] * TIR1[t] - la * auxTIR1[t] * IAAp[t]},
   {la * IAAp[t] * auxTIR1[t]}, {0}, {-2 * qa * (ARF[t] ^ 2) - pa * ARF[t] * IAAp[t]},
   {pa * ARF[t] * IAAp[t]}, {qa * (ARF[t]) ^ 2}, {pert - ka * aux[t] * TIR1[t]}};
bpertneg = {{lambda1 * F1neg + lambda2 * F2},
   {-la * IAAp[t] * auxTIR1[t] - pa * IAAp[t] * ARF[t]},
   {-ka * aux[t] * TIR1[t]}, {ka * aux[t] * TIR1[t] - la * auxTIR1[t] * IAAp[t]},
   {la * IAAp[t] * auxTIR1[t]}, {0}, {-2 * qa * (ARF[t] ^ 2) - pa * ARF[t] * IAAp[t]},
   {pa * ARF[t] * IAAp[t]}, {qa * (ARF[t]) ^ 2}, {pert - ka * aux[t] * TIR1[t]}};
```

Operative definitions of the systems (with parameters’ values inserted)

```
Mop = M /. param;
bopconst = bconst /. param;
Mopneg = M /. paramreg;
bopneg = bneg /. paramreg;

RHSneg = Mopneg.vt + bopneg;
RHSconst = Mop.vt + bopconst;
```

```
eqsneg = Table[vdt[[i]] == RHSneg[[i]], {i, 10}];
 eqsconst = Table[vdt[[i]] == RHSconst[[i]], {i, 10}];
eqssysneg = Join[eqsneg, ics];
eqssysconst = Join[eqsconst, ics];
```

```
Block[{ tinit = 0., tfin = 10 000., Sauxin = 0.02},
 nsol = NDSolve[eqssysneg, vt, {t, tinit, tfin}];
 csol = NDSolve[eqssysconst, vt, {t, tinit, tfin}];];
```

Jacobian and steady-state concentrations: definitions

We hereby define the Jacobian matrices for the negative feedback and the unregulated case.

```
replacess = Table[vt[[n]] → ss[[n]], {n, 1, 10}];
RHSssneg = RHSneg /. replacess;
RHSssconst = RHSconst /. replacess;
RHSNDssF[IAAmss, IAApss, TIR1ss, auxTIR1ss,
   auxTIR1IAAss, IAAstarss, ARFSS, ARFIAAss, ARF2ss, auxss] := RHSssneg;
RHSNDssFconst[IAAmss, IAApss, TIR1ss, auxTIR1ss, auxTIR1IAAss,
   IAAstarss, ARFSS, ARFIAAss, ARF2ss, auxss] := RHSssconst;
```

```
Jacob =
  Table[D[RHSNDssF[IAAmss, IAApss, TIR1ss, auxTIR1ss, auxTIR1IAAss, IAAstarss, ARFSS,
       ARFIAAss, ARF2ss, auxss][[m]][[1]], ss[[n]]], {m, 1, 10}, {n, 1, 10}];
Jacobc = Table[D[RHSNDssFconst[IAAmss, IAApss, TIR1ss, auxTIR1ss,
       auxTIR1IAAss, IAAstarss, ARFSS, ARFIAAss, ARF2ss, auxss][[
      m]][[1]], ss[[n]]], {m, 1, 10}, {n, 1, 10}];
```

We define the steady-state behaviour for all species.

```
P = pa / pd;
Q = qa / qd;
L = la / (ld + lm);
K = ka / kd;
auxTIR1IAAss = IAApss * K * L * TIR1ss * Sauxin / muaux;
auxss = Sauxin / muaux;
ARFSS = 1 / (4 Q) * (- (1 + P * IAApss) + Sqrt[(1 + P * IAApss) ^ 2 + 8 * Q * ARFT]);
ARF2ss = Q * ARFSS ^ 2;
ARFIAAss = P * ARFSS * IAApss;
TIR1ss = TIR1T / (1 + K * Sauxin * muaux + K * Sauxin * L * IAApss / muaux);
IAAstarss = auxTIR1IAAss * lm / muIAAstar;
auxTIR1ss = K * TIR1ss * auxss;
IAAmssneg = (lambda1 / muIAAm) * F1ssneg;
IAAmssconst = (lambda1 / muIAAm) * F1ssconst;
FMAX = Block[{Sauxin = Exp[8]}, NSolve[
    {(((la - ld * L) * K * Sauxin / muaux * IAApss * TIR1ss - lambda1 * delta / muIAAm *
          F1ssneg + muIAA * IAApss) /. paramreg) == 0, IAApss > 0.}, IAApss]];
ARF2ss /. param /. FMAX;
ARFSS /. param /. FMAX[[1]];
```

We start replacing the steady-state behaviours into the Jacobian matrices.

```
Jacobneg = Jacob /. {IAAmss → IAAmssneg};
Jacobconst = Jacobc /. {IAAmss → IAAmssconst};
```

Linear Response Analysis

Hereby we define the value of the amplitude of auxin influx perturbation we are going to apply and compute the corresponding steady-state value of IAA.

```
ϵ = 0.02;

initneg = Block[{Sauxin = ϵ},
   NSolve[{(((la - ld * L) * K * Sauxin / muaux * IAApss * TIR1ss - lambda1 *
            delta / muIAAm * F1ssneg + muIAA * IAApss) /. paramreg) == 0,
     IAApss > 0.}, IAApss, WorkingPrecision → 10]];
initconst = Block[{Sauxin = ϵ}, NSolve[
    {(((la - ld * L) * K * Sauxin / muaux * IAApss * TIR1ss -
           lambda1 * delta / muIAAm * F1ssconst + muIAA * IAApss) /. param) == 0,
     IAApss > 0.}, IAApss, WorkingPrecision → 10]];
```

We compute the Jacobian matrices with this steady states for the negative feedback and the unregulated case.

```
J = Jacobneg /. paramreg /. initneg[[1]] /. Sauxin → ϵ;
J1 = Jacobconst /. param /. initconst[[1]] /. Sauxin → ϵ;
```

PCA on the Jacobian

We implement the Principal Component Analysis described into the Supplementary Material to understand whether some species may be clustered according to their relaxation time after auxin perturbation. This will consist in computing the eigenvectors of the two Jacobian matrices, i.e., negative feedback and unregulated case, and plot the components corresponding to the modes associated to the first two highest eigenvalues.

The following list of species is needed only for visualisation.

```
listspecies = {"IAAm", "IAA", "TIR1", "Aux-TIR1",
   "Aux-TIR1-IAA", "IAA*", "ARF", "ARF-IAA", "ARF2", "Aux", "IAA2"};
```

```
p = ListPlot[Table[Labeled[{Eigenvectors[J][[1]][[n]], Eigenvectors[J][[2]][[n]]},
     listspecies[[n]]], {n, 1, 10}], PlotRange → All,
  AxesLabel → {"<i|w1>", "<i|w2>"}, PlotMarkers → {"●", 14}, PlotStyle → Red]
```

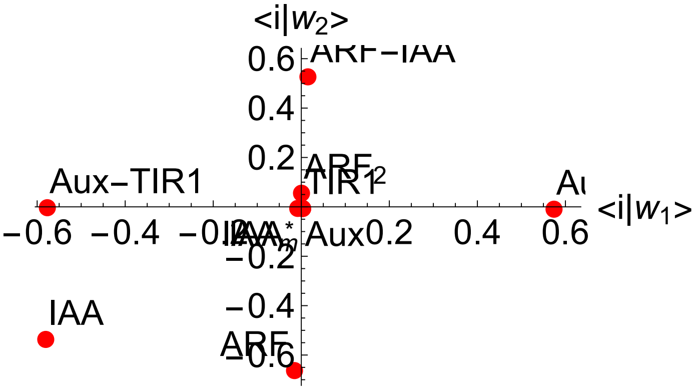


```
p2 =
 ListPlot[Table[Labeled[{Eigenvectors[J1][[1]][[n]], Eigenvectors[J1][[2]][[n]]},
     listspecies[[n]]], {n, 1, 10}], PlotRange → All,
  AxesLabel → {"<i|w1>", "<i|w2>"}, PlotMarkers → {"●", 14}, PlotStyle → Red]
```

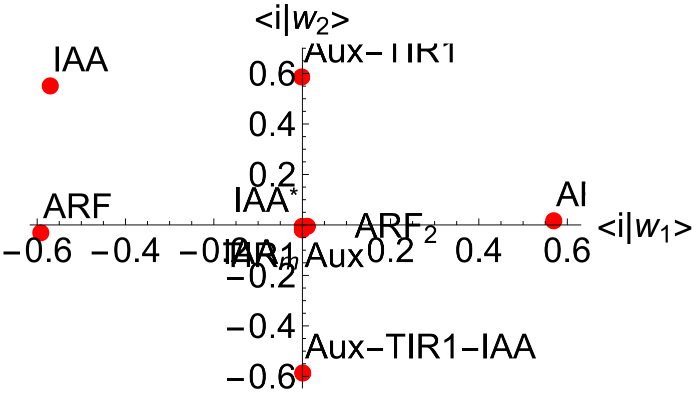

Plots of the temporal responses

We compute first the exponential of the jacobian matrices.

```
RT = MatrixExp[J * t];
RT1 = MatrixExp[J1 * t];
```

We plot the components corresponding to the response of IAA, ARF and auxin with respect to variations in auxin.

```
Plot[{Chop@RT[[2, 10]], Chop@RT1[[2, 10]]}, {t, 0., 2000},
 PlotRange → {All, All}, PlotStyle → {{Red, Dashed}, {Black, Thick}},
 PlotLegends → {"Reg", "No Reg"}, Frame → True,
 FrameLabel → {"t(min)", "\!\(\*SubscriptBox[\(χ\), \(IAA\)]\)(t)"}]
```

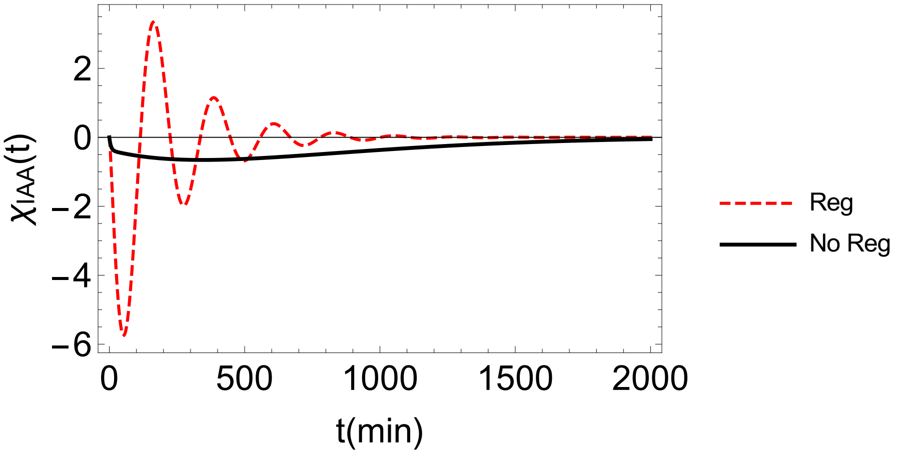


```
Plot[{Chop@RT[[7, 10]], Chop@RT1[[7, 10]]}, {t, 0., 2000},
 PlotRange → {{0., 2000}, All}, PlotStyle → {{Red, Dashed}, {Black, Thick}},
 PlotLegends → {"Reg", "No Reg"}, Frame → True,
 FrameLabel → {"t(min)", "\!\(\*SubscriptBox[\(χ\), \(ARF\)]\)(t)"}]
```

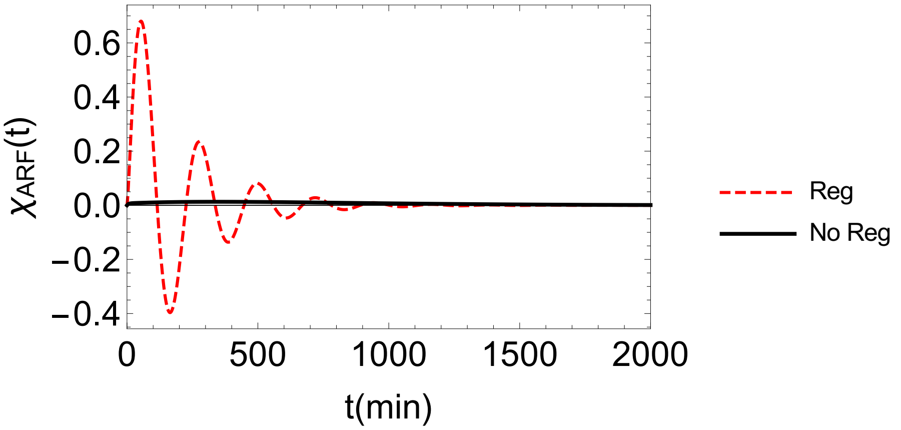

```
Plot[{RT[[10, 10]], RT1[[10, 10]]}, {t, 0., 2000}, PlotRange → {{0., 2000}, All},
 PlotStyle → {{Red, Dashed}, {Black, Thick}}, PlotLegends → {"Reg", "No Reg"},
 Frame → True, FrameLabel → {"t(min)", "\!\(\*SubscriptBox[\(χ\), \(Aux\)]\)(t)"}]
```

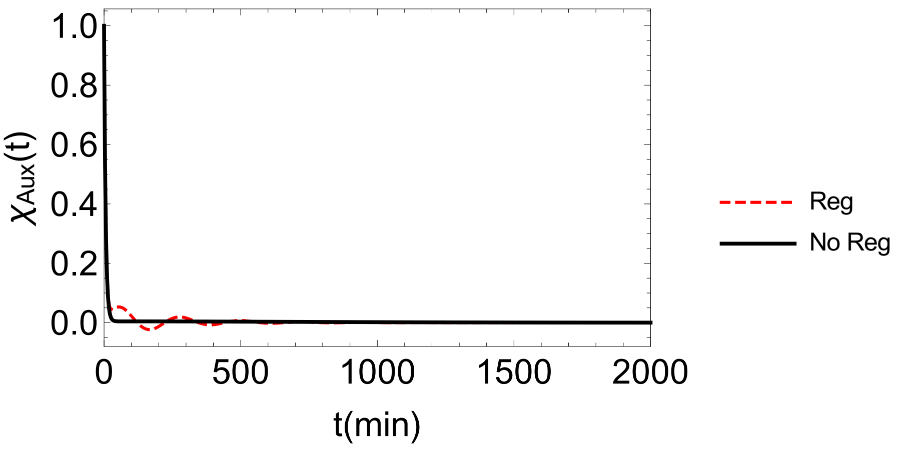

Toggle-switch implementation

Hereby we define the step-size perturbation for the auxin influx and we implement the equations for the auxin network with this perturbation.

```
omega0 = 0.02;
alpha = 0.2;
alpha0 = alpha;
pert2[t_, tc_, ti_, tm_] :=
 If[ti <= t <= tc, omega0 + alpha, omega0];
```

```
Plot[pert2[t, 50., 0., 200.], {t, -100., 500.}, Frame → True,
 FrameLabel → {"t (min)", "Sauxin (nM/min)"}, PlotStyle → {Black, Thick}]
```

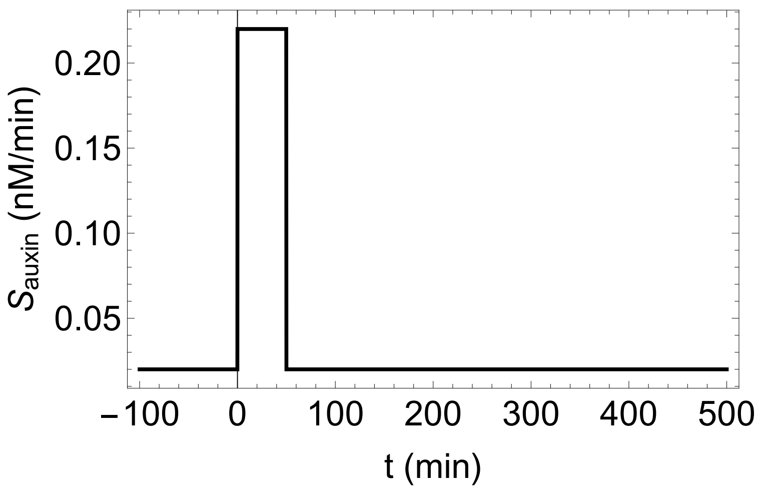

We compute first the steady-state values of the species in the auxin network for the basal rate of auxin influx, i.e., before adding the step-size perturbation.

```
tinit = -100;
tfin = 120 000;
Mop = M /. param;
Mopneg = M /. paramreg;
bopneg = bneg /. paramreg /. {Sauxin → omega0};
bopconst = bconst /. param /. {Sauxin → omega0};

RHSneg = Mopneg.vt + bopneg;
RHSconst = Mop.vt + bopconst;

eqneg = Table[vdt[[i]] == RHSneg[[i]], {i, 10}];
eqconst = Table[vdt[[i]] == RHSconst[[i]], {i, 10}];


eqssysSSneg = Join[eqneg, ics];
eqssysSSconst = Join[eqconst, ics2];
SSneg = NDSolve[eqssysSSneg, vt, {t, tinit, tfin}];
SSconst = NDSolve[eqssysSSconst, vt, {t, tinit, tfin}];

(*initial condition for the negative feedback*)
icsneglist = Table[Evaluate[vt[[i]] /. SSneg][[1]] /. {t → tfin}, {i, 1, 10}];
icsconstlist = Table[Evaluate[vt[[i]] /. SSconst][[1]] /. {t → tfin}, {i, 1, 10}];
icsneg = Table[
    {vt[[i]] == Table[Evaluate[vt[[i]] /. SSneg][[1]] /. {t → tfin}, {i, 1, 10}][[i]]},
    {i, 1, 10}] /. {t → tinit};
(*initial condition for no regulation*)
icsconst =
  Table[{vt[[i]] == Table[Evaluate[vt[[i]] /. SSconst][[1]] /. {t → tfin}, {i, 1, 10}][[
       i]]}, {i, 1, 10}] /. {t → tinit};
```

```
pert = pert2[t, tc, ti, tm];
Mopn = M /. paramreg;
Mopc = M /. param;
bopneg = bneg /. paramreg /. {Sauxin → omega0};
bopconst = bconst /. param /. {Sauxin → omega0};

bpertnegop = bpertneg /. paramreg /. {Sauxin → omega0};
bpertconstop = bpertconst /. param /. {Sauxin → omega0};

RHSneg = Mopn.vt + bopneg;
RHSconst = Mopc.vt + bopconst;
RHSpertneg = Mopn.vt + bpertnegop;
RHSpertconst = Mopc.vt + bpertconstop;

eqspertneg = Table[vdt[[i]] == RHSpertneg[[i]], {i, 10}];
eqspertconst = Table[vdt[[i]] == RHSpertconst[[i]], {i, 10}];

eqssysneg = Join[eqspertneg, icsneg];
eqssysconst = Join[eqspertconst, icsconst];
```

Here we implement the toggle switch by definying the parameters and the steady-state equations and their solution.

```
α1 = 0.5;
α2 = 0.5;
β = 4.;
γ = 4.;
θarf = 4.5;
tarf = 2;
δu = 0.1;
δv = 0.1;

toggleswitchsolneg = {ssu, ssv} /. NSolve[
    {0. == tarf icsneglist[[7]] / (θarf + icsneglist[[7]]) + α1 / (1. + ssv^β) - δu ssu,
     0. == α2 / (1. + ssu^γ) - δv ssv}, {ssu, ssv}, Reals];
toggleswitchsolconst = {ssu, ssv} /. NSolve[
    {0. == tarf icsconstlist[[7]] / (θarf + icsconstlist[[7]]) + α1 / (1. + ssv^β) - δu ssu,
     0. == α2 / (1. + ssu^γ) - δv ssv}, {ssu, ssv}, Reals];
```

We define hereby the dynamical equations for the toggle switch and set the steady-state values as initial conditions for the integration with a step-size perturbation.

```
toggleswitch = {u'[t] == tarf (ARF[t] / (θarf + ARF[t])) + α1 / (1. + v[t]^β) - δu u[t],
   v'[t] == α2 / (1. + u[t]^γ) - δv v[t]};
icstoggleneg = {u[tinit] == toggleswitchsolneg[[2, 1]],
   v[tinit] == toggleswitchsolneg[[2, 2]]};
icstoggleconst = {u[tinit] == toggleswitchsolconst[[2, 1]],
   v[tinit] == Chop@toggleswitchsolconst[[2, 2]]};
```

We prepare the final big system of ODEs by merging together the equations of the auxin network and

the toggle switch along with all initial conditions.

```
eqssysneg = Join[eqspertneg, toggleswitch, icsneg, icstoggleneg];
eqssysconst = Join[eqspertconst, toggleswitch, icsconst, icstoggleconst];
```

We integrate the equations for the negative feedback and the case without regulation.

```
tfin = 300;
tc = 50;
ti = 0;
tm = 200;
```

```
nsol =
 NDSolve[eqssysneg, Join[vt, {u[t], v[t]}], {t, tinit, tfin}, MaxStepSize → 0.01];
csol = NDSolve[eqssysconst, Join[vt, {u[t], v[t]}], {t, tinit, tfin}];
```

Toggle-switch plots

```
Plot[{Evaluate[u[t]] /. nsol, Evaluate[u[t]] /. csol}, {t, tinit, 200},
 PlotRange → All, Frame → True, FrameLabel → {"t(min)", "Gene I"},
 PlotStyle → {{Dashed, Darker[Green, 0.2]}, {Thick, Darker[Green, 0.5]}},
 AxesOrigin → {-100, 0}]
```

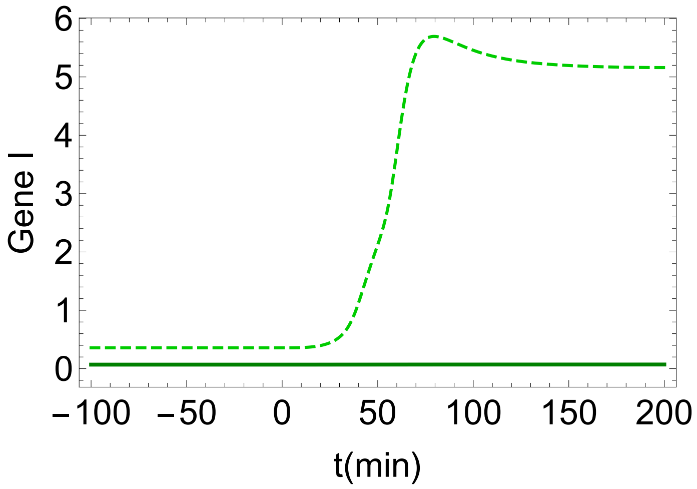

```
Plot[{Evaluate[v[t]] /. nsol, Evaluate[v[t]] /. csol}, {t, tinit, 200},
 PlotRange → All, Frame → True, FrameLabel → {"t(min)", "Gene II"},
 PlotStyle → {{Dashed, Red}, {Thick, Orange}}, AxesOrigin → {-100, 0}]
```

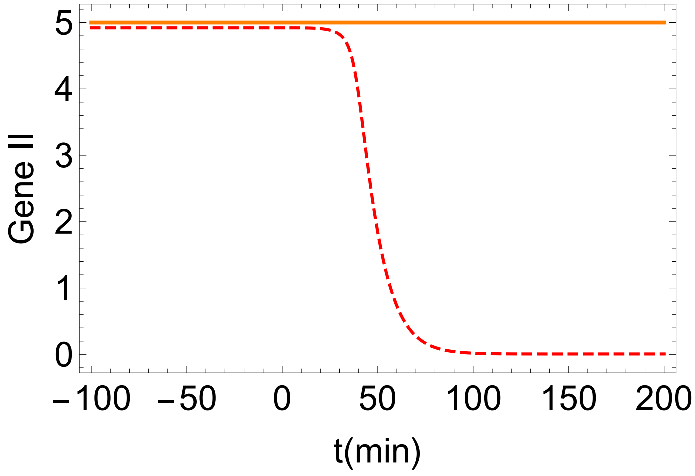

Stress gene response

We define the parameters for the stress-gene response.

```
α = 1;
β = 15;
δ = 0.005 / 4;
```

We define the new perturbation we shall apply to the system.

```
Clear[omega]
```

```
omega0 = 0.02;
omega = omega0;
alpha0 = 2;
tc0 = 100;
pert2[t_, alpha_, omega_, tc_] :=
 If[0 ≤ t ≤ tc, alpha * omega, omega];

Plot[pert2[t, alpha0, omega0, tc0], {t, -10., 2000.},
 Frame → True, FrameLabel → {"t (min)", "Sauxin (nM/min)"},
 PlotStyle → {Black, Thick}, PlotRange → All]
```

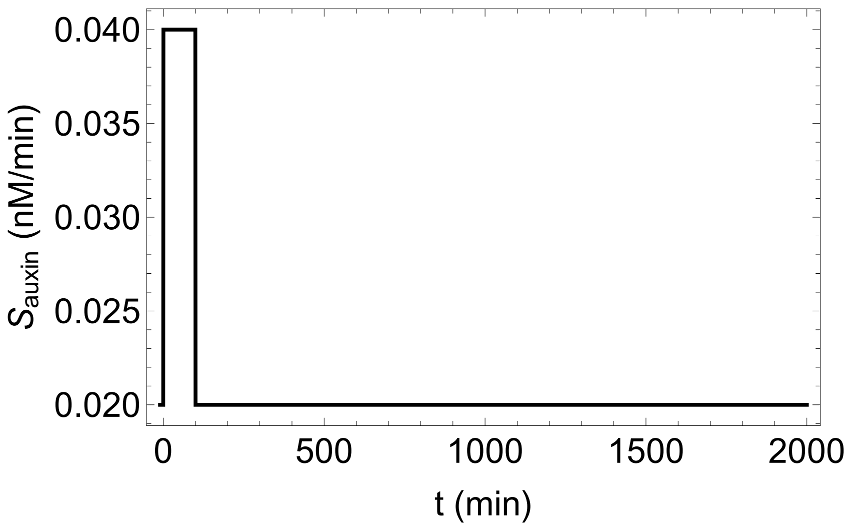

Hereby we compute the initial conditions as the steady-state values of the species for the basal auxin influx before applying the perturbation.

```
tinit = -5.;
tfin = 120 000;

Mop = M /. param;
Mopneg = M /. paramreg;
bopneg = bneg /. paramreg /. {Sauxin → omega0};
RHSneg = Mopneg.vt + bopneg;
eqneg = Table[vdt[[i]] == RHSneg[[i]], {i, 10}];
eqssysSSneg = Join[eqneg, ics];
SSneg = NDSolve[eqssysSSneg, vt, {t, tinit, tfin}];
icsneglist = Table[Evaluate[vt[[i]] /. SSneg][[1]] /. {t → tfin}, {i, 1, 10}];
icsneg = Table[
   {vt[[i]] == Table[Evaluate[vt[[i]] /. SSneg][[1]] /. {t → tfin}, {i, 1, 10}][[i]]},
   {i, 1, 10}] /. {t → tinit};
```

We redefine the perturbed system and prepare it for the final integration.

```
Clear[bpertnegop, bpertneg]
pert = pert2[t, alpha0, omega0, tc0];
bpertconst =
  {{lambda1 * F1const + lambda2 * F2}, {-la * IAAp[t] * auxTIR1[t] - pa * IAAp[t] * ARF[t]},
   {-ka * aux[t] * TIR1[t]}, {ka * aux[t] * TIR1[t] - la * auxTIR1[t] * IAAp[t]},
   {la * IAAp[t] * auxTIR1[t]}, {0}, {-2 * qa * (ARF[t] ^2) - pa * ARF[t] * IAAp[t]},
   {pa * ARF[t] * IAAp[t]}, {qa * (ARF[t]) ^2}, {pert - ka * aux[t] * TIR1[t]}};
bpertneg = {{lambda1 * F1neg + lambda2 * F2},
   {-la * IAAp[t] * auxTIR1[t] - pa * IAAp[t] * ARF[t]},
   {-ka * aux[t] * TIR1[t]}, {ka * aux[t] * TIR1[t] - la * auxTIR1[t] * IAAp[t]},
   {la * IAAp[t] * auxTIR1[t]}, {0}, {-2 * qa * (ARF[t] ^2) - pa * ARF[t] * IAAp[t]},
   {pa * ARF[t] * IAAp[t]}, {qa * (ARF[t]) ^2}, {pert - ka * aux[t] * TIR1[t]}};

Mopn = M /. paramreg;
Mopc = M /. param;
bopneg = bneg /. paramreg;
bpertnegop = bpertneg /. paramreg;
RHSneg = Mopn.vt + bopneg;
RHSpertneg = Mopn.vt + bpertnegop;

eqspertneg = Table[vdt[[i]] == RHSpertneg[[i]], {i, 10}];
eqssysneg = Join[eqspertneg, icsneg];
```

We solve the steady-state equations for the stress gene response and initial conditions for basal auxin influx before applying the perturbation. We merge these initial conditions and the dynamical equations with the entire system.

```
stressgenesol1 =
  e1 /. NSolve[0. == α icsneglist[[7]] / (β + icsneglist[[7]]) - δ e1, e1, Reals];
stressgenesol2 = e2 /. NSolve[
    0. == α icsneglist[[9]] / (β^2 + icsneglist[[9]]) - δ e2, e2, Reals];
stressgene = {e1'[t] == α (ARF[t] / (β + ARF[t])) - δ e1[t],
   e2'[t] == α (ARF2[t] / (β^2 + ARF2[t])) - δ e2[t]};
icsstressgeneneg = {e1[tinit] == stressgenesol1[[1]],
   e2[tinit] == stressgenesol2[[1]]};
eqssysneg = Join[eqspertneg, stressgene, icsneg, icsstressgeneneg];
```

We solve the final system.

```
tfin = 2000;
nsol = NDSolve[eqssysneg, Join[vt, {e1[t], e2[t]}], {t, tinit, tfin}];
```

Stress-gene response's plots

```
e1neg1 = ListLinePlot[
  Table[{t1, (Evaluate[e1[t]] /. nsol /. {t → t1}) [[1]]}, {t1, -5, tfin, 1}],
  PlotRange → {{-5, tfin}, All}, PlotStyle → {Thick, Red}]
```

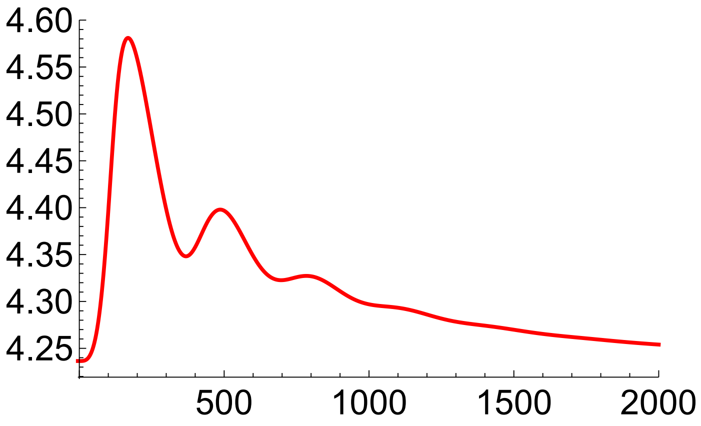


```
e2neg1 = ListLinePlot[
  Table[{t1, (Evaluate[e2[t]] /. nsol /. {t → t1}) [[1]]}, {t1, -5, tfin, 1}],
  PlotRange → {{-5, tfin}, All}, PlotStyle → {Thick, Red}]
```

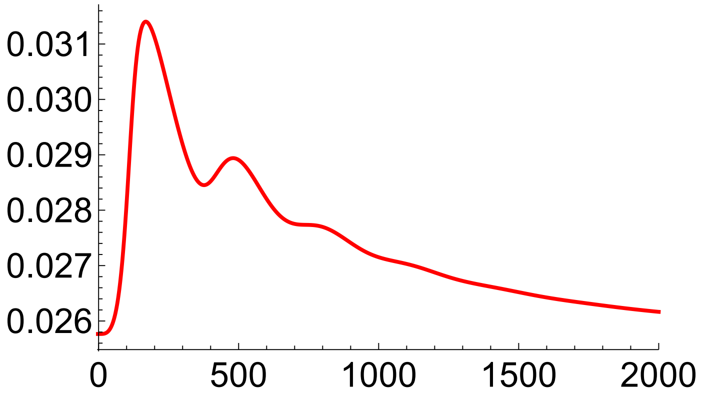

Steady State properties with varying omega

Hereby we define the lists of the steady states for IAA, ARF and auxin for the negative feedback and the unregulated case, respectively.

```
listpneg = {};
listtarfneg = {};
listaneg = {};
listpconst = {};
listaconst = {};
listtarfconst = {};
```

We define the range of variation of auxin influx.

```
lwwinit = -10.;
lwwfin = Log[0.5];
lwwstep = 0.1;
wwinit = Exp[lwwinit];
wwfin = Exp[lwwfin];
```

We initialise the steady states.

```
P = pa / pd;
Q = qa / qd;
L = la / (ld + lm);
K = ka / kd;
auxTIR1IAAss = (IAApss * K * L * TIR1ss * Sauxin) / muaux;
auxss = Sauxin / muaux;
ARFSS = 1 / (4 Q) * (- (1 + P * IAApss) + Sqrt[(1 + P * IAApss) ^ 2 + 8 * Q * ARFT]);
ARF2ss = Q * ARFSS ^ 2;
ARFIAAss = P * ARFSS * IAApss;
TIR1ss = TIR1T / (1 + K * Sauxin / muaux + K * Sauxin * L * IAApss / muaux);
IAAstarss = auxTIR1IAAss * lm / muIAAstar;
auxTIR1ss = K * TIR1ss * auxss;
IAA2ss = H * IAApss ^ 2.;
IAAmssneg = (lambda1 / muIAAm) * F1ssneg;
IAAmssconst = (lambda1 / muIAAm) * F1ssconst;
initneg =
  Block[{Sauxin = wwinit}, NSolve[{(((la - ld * L) * K * Sauxin / muaux * IAApss * TIR1ss -
          lambda1 * delta / muIAAm * F1ssneg + muIAA * IAApss) /.
        paramreg) == 0, IAApss > 0.}, IAApss]] // Quiet;
initconst = Block[{Sauxin = wwinit}, NSolve[
    {(((la - ld * L) * K * Sauxin / muaux * IAApss * TIR1ss - lambda1 * delta / muIAAm *
          F1ssconst + muIAA * IAApss) /. param) == 0, IAApss > 0.}, IAApss]] // Quiet;
```

We start varying the auxin influx and find the solution to the self-consistency equation. We use this result to recompute all the steady states at each step.

```
For[lww = lwwinit, lww ≤ lwwfin, lww += lwwstep,

  ww = Exp[lww];
  initcondsneg = IAApss /. paramreg /. initneg[[1]];
  initcondsconst = IAApss /. param /. initconst[[1]];

  newneg = Block[{Sauxin = ww}, Assuming[IAApss > 0., FindRoot[
       (((la - ld * L) * K * Sauxin / muaux * IAApss * TIR1ss - lambda1 * delta / muIAAm *
            F1ssneg + muIAA * IAApss) /. paramreg) == 0, {IAApss, initcondsneg}]]];
    newconst = Block[{Sauxin = ww}, Assuming[IAApss > 0., FindRoot[
       (((la - ld * L) * K * Sauxin / muaux * IAApss * TIR1ss - lambda1 * delta / muIAAm *
            F1ssconst + muIAA * IAApss) /. param) == 0, {IAApss, initcondsconst}]]];

  listpneg = Append[listpneg, {ww, IAApss} /. newneg];
  listtarfneg =
   Append[listtarfneg, {ww, ARFSS} /. paramreg /. newneg /. {Sauxin → ww}];
  listaneg = Append[listaneg, {ww, auxss} /. paramreg /. newneg /. {Sauxin → ww}];
  listpconst = Append[listpconst, {ww, IAApss} /. newconst];
  listaconst = Append[listaconst, {ww, auxss} /. param /. newconst /. {Sauxin → ww}];
  listtarfconst =
   Append[listtarfconst, {ww, ARFSS} /. param /. newconst /. {Sauxin → ww}];
  initneg[[1]] = newneg;
  initconst[[1]] = newconst;
 ] // Quiet

iaaplotnew = ListLinePlot[{listpneg[[All, All]], listpconst[[All, All]]},
  PlotRange → {All, {0, 150}}, Frame → True,
  FrameLabel → {"\!\(\*SubscriptBox[\(S\), \(auxin\)]\)(nM/min)", "Aux/IAA(nM)"},
  PlotStyle → {{Red, Dashed}, {Black, Thick}}, AxesOrigin → {0, 0}]
```

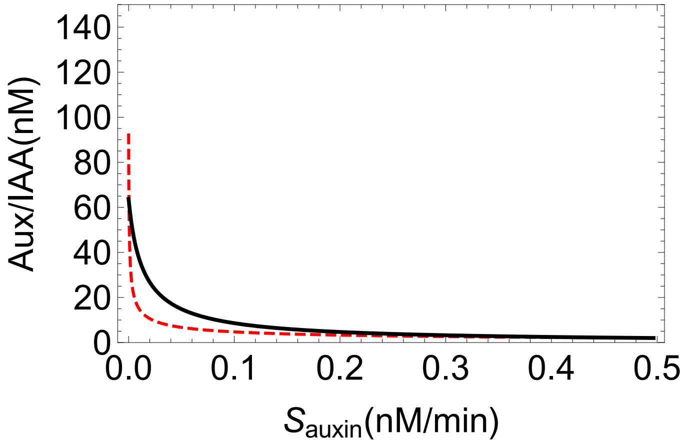

```
arfplotnew = ListLinePlot[{listtarfneg[[All, All]], listtarfconst[[All, All]]},
  PlotRange → {{0, 0.5}, All}, Frame → True,
  FrameLabel → {"\!\(\*SubscriptBox[\(S\), \(auxin\)]\)(nM/min)", "ARF(nM)"},
  PlotStyle → {{Red, Dashed}, {Black, Thick}}]
```

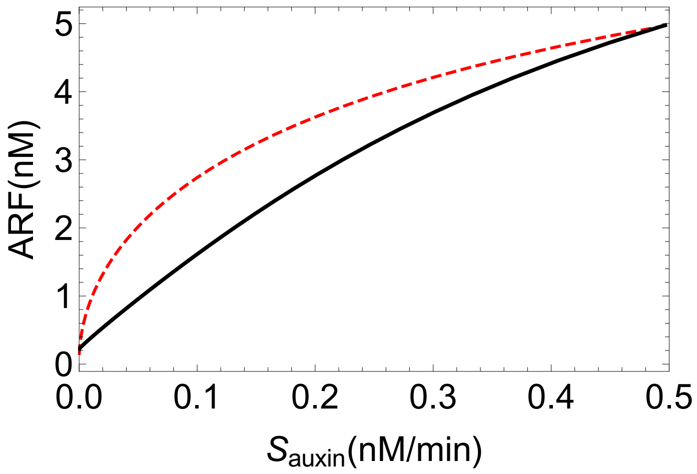


```
auxplotnew = ListLinePlot[{listaneg[[All, All]], listaconst[[All, All]]},
  PlotRange → {{0, 0.5}, All}, Frame → True,
  FrameLabel → {"\!\(\*SubscriptBox[\(S\), \(auxin\)]\)(nM/min)", "auxin(nM)"},
  PlotStyle → {{Black, Thick}, {Red, Dashed}}]
```

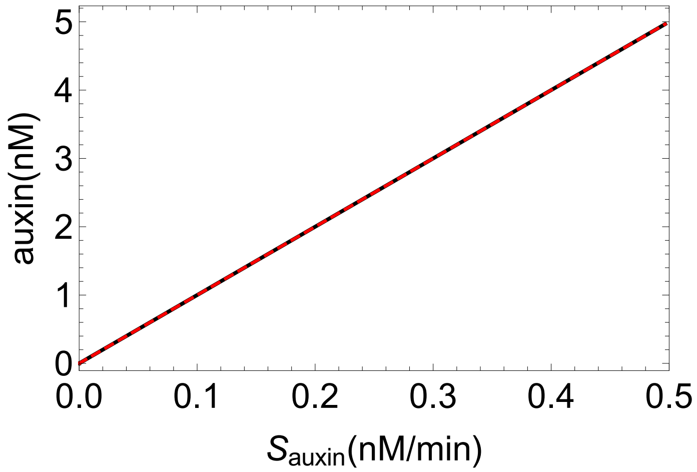

Self-consistency equations and plot

Here we define the steady-state concentrations for the species we need to plot the self-consistency equation. We then define the two sides of the equations and finally plot them for different values of auxin influx.

```
ARFSS = 1 / (4 Q) * (- (1 + P * IAApss) + Sqrt[(1 + P * IAApss) ^2 + 8 * Q * ARFT]);
ARF2ss = Q * ARFSS ^ 2;
ARFIAAss = P * ARFSS * IAApss;
```

```
ARF[x_] := (- (1 + pa / pd x) + Sqrt[(1 + pa / pd x) ^ 2 + 8 qa / qd ARFT]) / (4 (qa / qd));
ARFIAA[x_] := pa / pd ARF[x] x;
ARF2[x_] := qa / qd ARF[x] ^ 2;
fleft[x_] := delta / muIAAm lambda1 ARF[x] / thetaARF /
     (1 + ARF[x] / thetaARF + ARFIAA[x] / thetaARFIAA + ARF2[x] / thetaARF2) - muIAA x;
fright[x_] := (la - ld * L) ka / kd Sauxin / muaux x
   TIR1T / (1 + ka / kd Sauxin / muaux + la / (ld + lm) ka / kd Sauxin / muaux x);

fright100 = Plot[fright[x] /. paramreg /. {Sauxin → 0.001},
   {x, 0, 100}, PlotStyle → {Gray, Dashed}, PlotRange → All];
fright10 = Plot[fright[x] /. paramreg /. {Sauxin → 0.01}, {x, 0, 100},
   PlotStyle → {Gray, Lighter, Thickness[0.008]}, PlotRange → All];
fright1 = Plot[fright[x] /. paramreg /. {Sauxin → 0.1}, {x, 0, 100},
   PlotStyle → {Black, Dotted, Thickness[0.005]}, PlotRange → All];

fleftgeneral =
  Plot[fleft[x] /. paramreg, {x, 0, 100}, PlotStyle → {Orange, Thickness[0.008]},
   PlotRange → {All, {0, 20}}, Frame → True, FrameLabel → {"IAA", ""}];

Show[fleftgeneral, fright100, fright10, fright1]
```

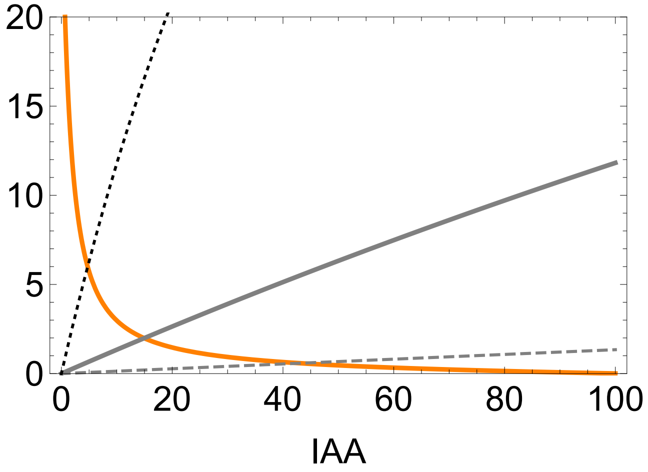

Supplement: Supplementary Text [file rsos172098supp1.pdf]
